# P38 kinases mediate NLRP1 inflammasome activation after ribotoxic stress response and virus infection

**DOI:** 10.1084/jem.20220837

**Published:** 2022-10-31

**Authors:** Lea-Marie Jenster, Karl-Elmar Lange, Sabine Normann, Anja vom Hemdt, Jennifer D. Wuerth, Lisa D.J. Schiffelers, Yonas M. Tesfamariam, Florian N. Gohr, Laura Klein, Ines H. Kaltheuner, Stefan Ebner, Dorothee J. Lapp, Jacob Mayer, Jonas Moecking, Hidde L. Ploegh, Eicke Latz, Felix Meissner, Matthias Geyer, Beate M. Kümmerer, Florian I. Schmidt

**Affiliations:** 1 Institute of Innate Immunity, Medical Faculty, University of Bonn, Bonn, Germany; 2 Institute of Virology, Medical Faculty, University of Bonn, Bonn, Germany; 3 Department of Microbiology and Immunology, The University of Melbourne, Parkville, Victoria, Australia; 4 Institute of Structural Biology, Medical Faculty, University of Bonn, Bonn, Germany; 5 Program in Cellular and Molecular Medicine, Boston Children’s Hospital, Boston, MA; 6 German Centre for Infection Research, Partner Site Bonn-Cologne, Bonn, Germany; 7 Core Facility Nanobodies, Medical Faculty, University of Bonn, Bonn, Germany

## Abstract

Inflammasomes integrate cytosolic evidence of infection or damage to mount inflammatory responses. The inflammasome sensor NLRP1 is expressed in human keratinocytes and coordinates inflammation in the skin. We found that diverse stress signals induce human NLRP1 inflammasome assembly by activating MAP kinase p38: While the ribotoxic stress response to UV and microbial molecules exclusively activates p38 through MAP3K ZAKα, infection with arthropod-borne alphaviruses, including Semliki Forest and Chikungunya virus, activates p38 through ZAKα and potentially other MAP3K. We demonstrate that p38 directly phosphorylates NLRP1 and that serine 107 in the linker region is critical for activation. NLRP1 phosphorylation is followed by ubiquitination of NLRP1^PYD^, N-terminal degradation of NLRP1, and nucleation of inflammasomes by NLRP1^UPA-CARD^. In contrast, activation of NLRP1 by nanobody-mediated ubiquitination, viral proteases, or inhibition of DPP9 was independent of p38 activity. Taken together, we define p38 activation as a unifying signaling hub that controls NLRP1 inflammasome activation by integrating a variety of cellular stress signals relevant to the skin.

## Teaser

Jenster et al. describe how p38 signaling integrates cues of cellular stress to activate human NLRP1 by direct phosphorylation. This novel pathway is activated by physiologically relevant threats to the skin, including the UVB-triggered ribotoxic stress response or infection with alphaviruses, such as Chikungunya virus.

## Graphical Abstract

**Figure:**
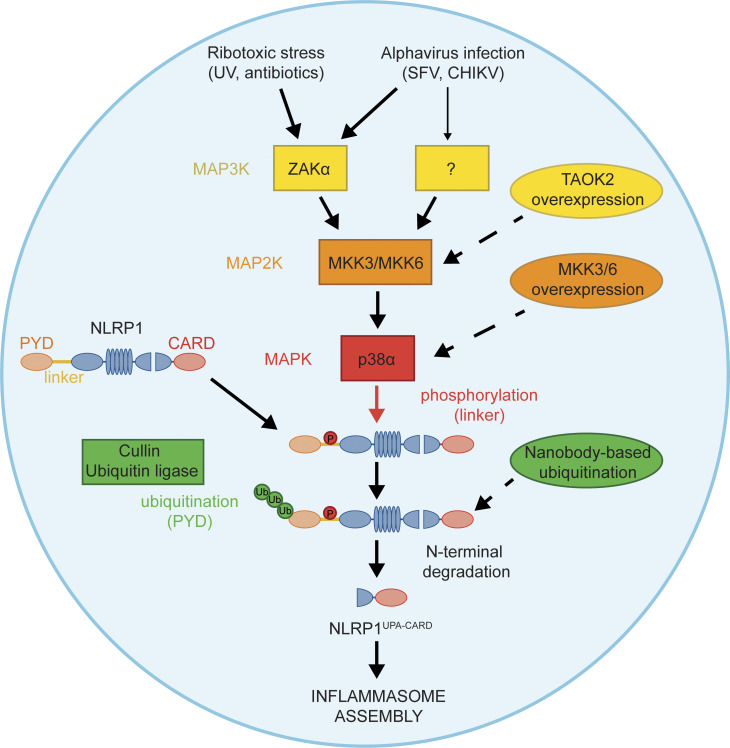

## Introduction

Inflammatory responses to counteract infection or tissue damage are orchestrated by a multi-layered network of locally and systemically acting signaling cascades. Nucleotide-binding domain and leucine-rich repeat (LRR) containing proteins (NLRs) are critical for the detection and regulation of the early innate immune response in mammalian cells. NLRP1, NLRP3, and other family members initiate the formation of cytosolic multi-protein complexes described as inflammasomes (Broz and Dixit, 2016; Tschopp et al., 2003; Mitchell et al., 2019). In response to sensor-specific stimulation, they recruit and nucleate polymerization of the adaptor protein ASC, resulting in micrometer-sized ASC specks within the cell that represents potent caspase-1–activating platforms (Broz et al., 2010). Mature caspase-1 processes the proinflammatory cytokines IL-1β, IL-18, and the pore-forming protein gasdermin D, which executes pyroptotic cell death (Broz and Dixit, 2016).

Human NLRP1 exhibits a unique domain structure and contains both an N-terminal pyrin domain (PYD) and a C-terminal caspase recruitment domain (CARD; Yu et al., 2018; Martinon et al., 2002). Autoproteolytic processing of NLRP1 in the “function to find domain” (FIIND) is required for inflammasome formation as it generates the FIIND^UPA^-CARD fragment, which can oligomerize and recruit ASC to initiate the inflammasome response (Finger et al., 2012; Sandstrom et al., 2019; Hollingsworth et al., 2021b; Gong et al., 2021; D’Osualdo et al., 2011). In steady state, FIIND^UPA^-CARD remains associated with the N-terminus; FIIND^UPA^-CARD released from the N-terminus can also be sequestered by association with the dipeptidyl peptidase 9 (DPP9; Hollingsworth et al., 2021a; Zhong et al., 2018; Okondo et al., 2018; Huang et al., 2021). FIIND^UPA^-CARD is released by the inhibition of DPP9 (Okondo et al., 2018; Zhong et al., 2018) or by degradation of the NLRP1 N-terminus. The latter activation mechanism enables NLRP1 to detect protease activity of pathogens, as shown for enteroviral proteases (Robinson et al., 2020; Tsu et al., 2021). Other reported stimuli of NLRP1 are ATP depletion (Liao and Mogridge, 2013), UV irradiation (Feldmeyer et al., 2007; Fenini et al., 2018a), Semliki Forest virus (SFV) infection, and double-stranded RNA (dsRNA; Bauernfried et al., 2021). How signaling by these diverse triggers converge, and how they activate NLRP1, is not understood.

In rodent Nlrp1b, the N-terminal PYD is replaced by unrelated sequences (Yu et al., 2018). While activation by Dpp9 inhibition is shared with human NLRP1 (Gai et al., 2019; de Vasconcelos et al., 2019), Nlrp1b can be activated by unique triggers such as anthrax toxin lethal factor (Levinsohn et al., 2012), but does not respond to SFV or dsRNA (Bauernfried et al., 2021). The human inflammasome sensor CARD8 exhibits an autoproteolytically processed FIIND^UPA^-CARD fragment similar to NLRP1 and represents the dominant sensor of DPP9 inhibition in a variety of leukocyte cell types and cell lines (Johnson et al., 2018). Importantly, CARD8 FIIND^UPA^-CARD directly engages pro-caspase-1 and is not able to assemble ASC specks (Gong et al., 2021).

In human keratinocytes, NLRP1, but not CARD8, assembles functional inflammasomes (Burian and Yazdi, 2018; Fenini et al., 2018a; Bauernfried et al., 2021). The prominent role of NLRP1 in the skin is underlined by strong pathological manifestations in the skin observed in patients bearing gain-of-function mutants of NLRP1 (Zhong et al., 2016; Herlin et al., 2020). In this study, we analyzed the signaling cascades upstream of NLRP1 and revealed a unified signaling pathway that activates NLRP1 in response to diverse cell stress signals: Both activation of the ribotoxic stress response, as well as viral infections, trigger activation of p38 MAPKs, which then phosphorylate the linker region of NLRP1 and thus initiate the ubiquitination of the NLRP1^PYD^, followed by N-terminal degradation and nucleation of inflammasomes by the C-terminal NLRP1^UPA-CARD^.

## Results

### Robust quantification of human NLRP1 inflammasome assembly by flow cytometry

**Figure 1.**
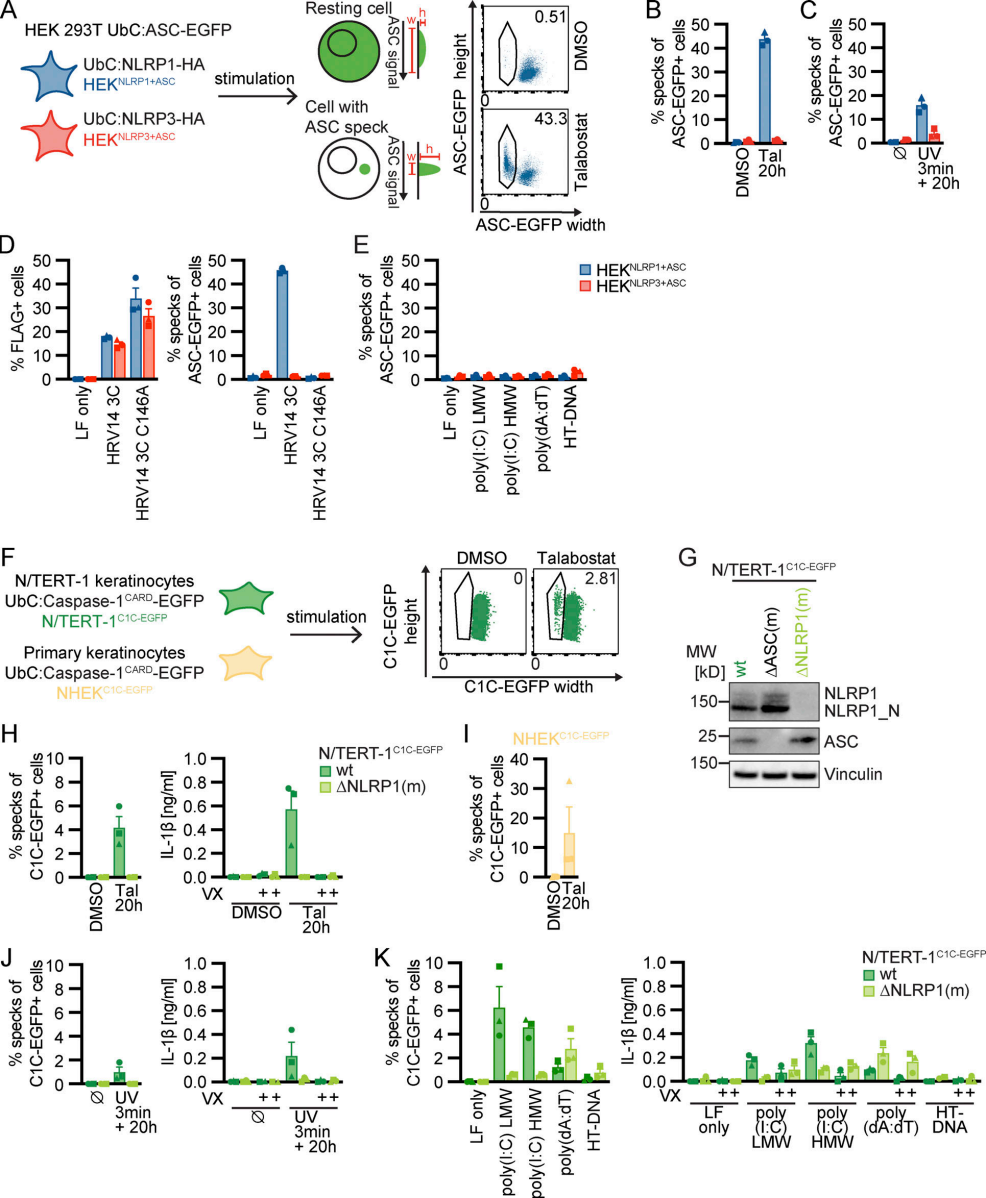
**Reporter cell lines recapitulate NLRP1 inflammasome assembly. (A)** Scheme of generated HEK 293T reporter cell lines and detection of ASC specks by flow cytometry. **(B–E)** HEK^NLRP1+ASC^ or HEK^NLRP3+ASC^ cells were treated with 30 µM Tal (B) or UV for 3 min (C), or were transfected with expression vectors for FLAG-tagged HRV 14 protease 3C (D), or 1 µg/ml of the indicated nucleic acid species (E). 20 h after treatment, ASC-EGFP–positive cells were analyzed by flow cytometry and the fraction of cells with ASC specks was determined with the gating strategy described in A. In D, cells were additionally stained for FLAG and ASC specks were only quantified in FLAG-positive cells for all samples transfected with plasmids, i.e., not in LF only controls. **(F)** Scheme of generated N/TERT-1 reporter cell lines and detection of C1C specks by flow cytometry. **(G)** N/TERT-1^C1C-EGFP^ cells and monoclonal ASC and NLRP1 knockout derivatives were analyzed by immunoblot with the indicated antibodies. **(H–K)** N/TERT-1^C1C-EGFP^ cells and their monoclonal NLRP1 knockout derivatives (H, J, and K) or NHEK^C1C-EGFP^ (I) were treated with the indicated stimuli for 20 h as described above. For flow cytometry experiments, cells were additionally treated with 100 µM VX. To quantify inflammasome assembly, C1C-EGFP–positive cells were analyzed by flow cytometry. The fraction of cells with C1C-EGFP specks was determined with the gating strategy described in F. IL-1β from the supernatants of cells stimulated in the absence or presence of 100 μM VX was quantified by HTRF (H, J, and K). Data represents average values (with individual data points) from three independent experiments ± SEM. UbC indicates ubiquitin C (promoter). MW, molecular weight. Source data are available for this figure: SourceData F1.

To identify and evaluate (patho)physiological triggers of NLRP1, we used two complementary cellular systems: In a bottom-up approach, we reconstituted NLRP1 inflammasome components in human embryonic kidney (HEK) 293T cells. To study human NLRP1 at endogenous levels in a physiologically relevant cell type, we used immortalized N/TERT-1 keratinocytes (Dickson et al., 2000; Fig. 1). As a readout for activation, we decided to evaluate the assembly of ASC specks, as all downstream effects of NLRP1 rely on the formation of these large macromolecular assemblies, and as CARD8 is not able to initiate ASC specks.

**Figure S1.**
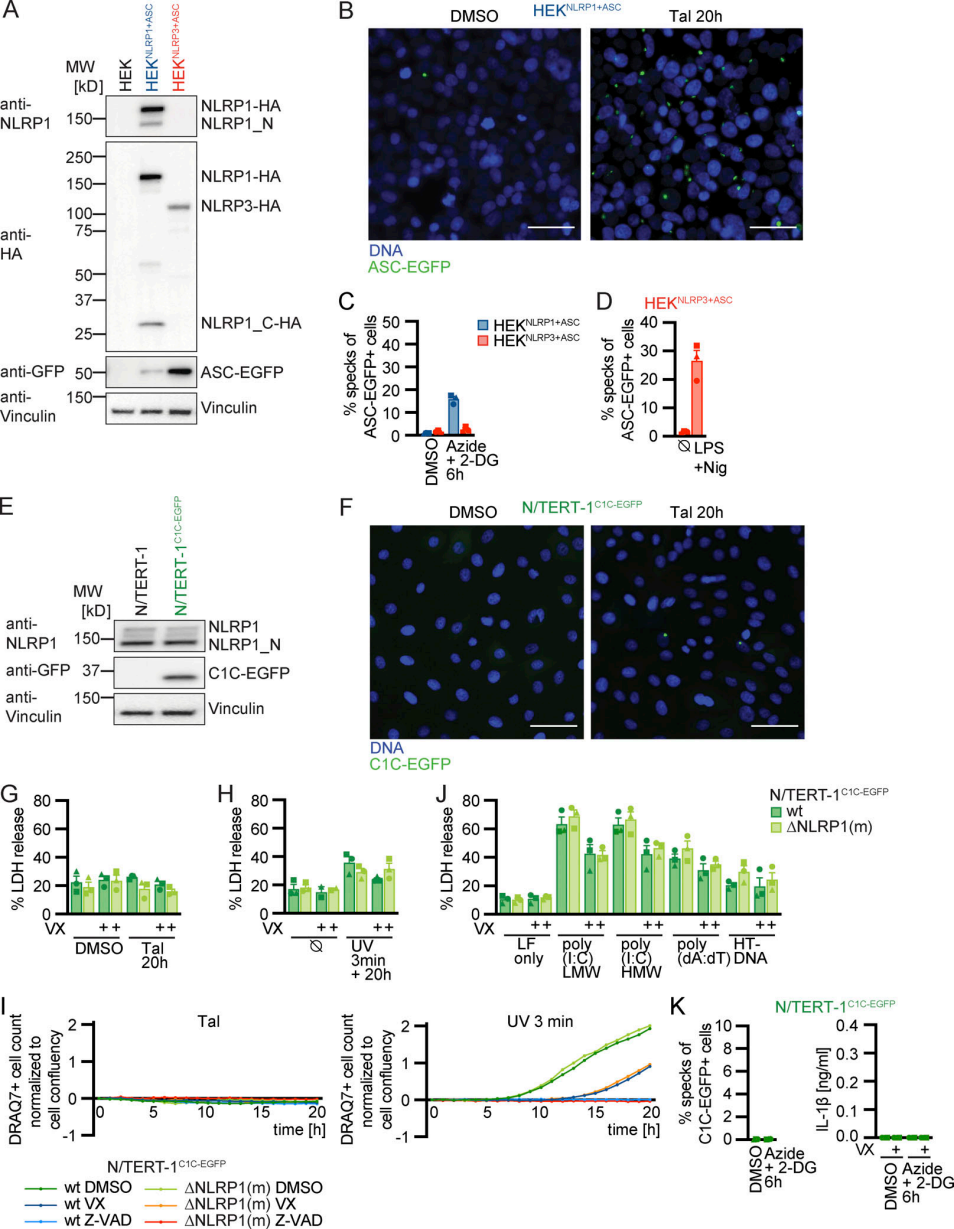
**Reporter cell lines recapitulate NLRP1 inflammasome assembly. (A)** Lysates of HEK 293T (HEK), HEK^NLRP1+ASC^, and HEK^NLRP3+ASC^ cells were analyzed by immunoblot with the indicated antibodies to confirm expression of NLRP1-HA, NLRP3-HA, and ASC-EGFP. **(B and F)** HEK^NLRP1+ASC^ or N/TERT-1^C1C-EGFP^ cells were seeded on cover slips and stimulated with DMSO or 30 µM Tal for 20 h as in (Fig. 1, B and H). Fixed cells were stained for DNA and representative images were recorded by wide-field fluorescence microscopy. Scale bars represent 50 μm. **(C and K)** HEK^NLRP1+ASC^, HEK^NLRP3+ASC^, or N/TERT-1^C1C-EGFP^ cells were treated with DMSO or 10 mM sodium azide and 50 mM 2-DG for 6 h. ASC-EGFP or C1C-EGFP speck formation was quantified by flow cytometry as described in Fig. 1, A and F. N/TERT-1^C1C-EGFP^ cells were stimulated in the presence of 100 µM VX for flow cytometry experiments. IL-1β from the supernatants of cells stimulated in the absence or presence of 100 μM VX was quantified by HTRF. **(D)** HEK^NLRP3+ASC^ cells were treated with 200 ng/ml LPS for 3 h and 10 µM Nig for 1 h, followed by quantification of ASC-EGFP specks by flow cytometry as in C. **(E)** Cell lysates of N/TERT-1 and N/TERT-1^C1C-EGFP^ cells were analyzed by immunoblot with the indicated antibodies to confirm expression of NLRP1 and C1C-EGFP. **(G–J)** N/TERT-1^C1C-EGFP^ cells and their monoclonal NLRP1 knockout derivatives were stimulated with 30 µM Tal (G and I), UV for 3 min (H and I), or transfected with 1 µg/ml of the indicated nucleic acid species (J) for 20 h as in Fig. 1, H, J, and K, where indicated in the presence of DMSO, 100 μM VX, or 50 μM Z-VAD. Cell death was quantified by detection of LDH release into the supernatant (G–J), or by uptake of non-cell permeable DNA dye DRAQ7 over 20 h (I). For LDH detection, the same supernatants as for IL-1β detection in Fig. 1, H, J, and K, were used. Data from all experiments quantifying specks, LDH release, or IL-1β release represents average values (with individual data points) from three independent experiments ± SEM. Immunoblots in A and E, microscopy images in B and F and quantifications of DRAQ7 uptake over time in I display experiment representatives of two or three independent experiments. MW, molecular weight. Source data are available for this figure: SourceData FS1.

We equipped a monoclonal HEK 293T cell line expressing ASC-EGFP from the weak ubiquitin C promoter (pUbC) with either HA-tagged NLRP1 or NLRP3, which was likewise under the control of pUbC (Fig. 1 A and Fig. S1 A). We next selected clones with optimal signal-to-noise ratio (HEK^NLRP1+ASC^ and HEK^NLRP3+ASC^), and quantified assembly of ASC specks by flow cytometry, exploiting the characteristic redistribution of ASC-EGFP into a single speck per cell. This resulted in cells with decreased width and increased height of the EGFP signal when compared to untreated cells with diffuse ASC-EGFP (Sester et al., 2015; Fig. 1 A). In HEK^NLRP1+ASC^ cells, we were able to recapitulate NLRP1 activation by the DPP9 inhibitor talabostat (Tal; Fig. 1, A and B; and Fig. S1 B; Zhong et al., 2018), by UV irradiation (Fig. 1 C; Fenini et al., 2018a), by transient expression of 3C protease of human rhinovirus (HRV) 14 (Fig. 1 D; Robinson et al., 2020; Tsu et al., 2021), and by ATP depletion with azide and 2-deoxyglucose (2-DG; Fig. S1 C; Liao and Mogridge, 2013). Although proposed as a direct NLRP1 ligand (Bauernfried et al., 2021), transfection of synthetic dsRNA poly(I:C) did not induce inflammasome activation above background levels, and neither did synthetic DNA poly(dA:dT) nor herring testis DNA (HT-DNA; Fig. 1 E). HEK^NLRP3+ASC^ cells did not respond to any of these stimuli, but they assembled ASC specks after treatment with LPS and nigericin (Nig), validating functional NLRP3 inflammasomes (Fig. S1 D).

To assess human NLRP1 inflammasome assembly in keratinocytes at endogenous levels of NLRP1 and ASC, we equipped N/TERT-1 keratinocytes known to express NLRP1 (Zhong et al., 2016) and primary normal human epidermal keratinocytes (NHEK) with a reporter construct composed of the caspase-1^CARD^ (C1C) fused to EGFP (Fig 1 F and Fig. S1 E). C1C-EGFP is efficiently recruited to ASC specks, allowing visualization and quantification of inflammasome assembly by fluorescence microscopy and flow cytometry, exploiting the characteristic redistribution of fluorescence into a single speck per cell as for ASC-EGFP (Fig. 1 F and Fig. S1 F). Unlike fluorescent fusions of ASC, however, this reporter does not alter the endogenous levels of ASC, cannot assemble specks by mere overexpression, and lastly also recapitulates the recruitment of caspase-1. All experiments for the quantification of specks in keratinocytes were performed in the presence of the caspase-1 inhibitor Vx-765 (VX) to avoid loss of responding cells by pyroptosis. The novel reporter will be described and characterized in greater detail in an independent study (in preparation). Unlike HEK 293T cells, N/TERT-1 keratinocytes express pro-IL-1β and undergo inflammasome-induced pyroptosis. We are thus able to validate the ASC speck readout by quantifying the secretion of caspase-1–matured IL-1β and (pyroptotic) cell death.

N/TERT-1^C1C-EGFP^ and NHEK^C1C-EGFP^ cells assembled inflammasomes after DPP9 inhibition (Fig. 1, F, H and I; and Fig. S1 F) and UV irradiation (Fig. 1 J), even though response rates were low compared to the engineered HEK 293T reporter system. This may be attributed to endogenous protein levels or a higher threshold of activation. Importantly, no specks whatsoever were detected in the absence of activation, which allowed us to confidently quantify inflammasome triggers with even low response rates. ASC specks were not observed in monoclonal NLRP1 knockout cells (Fig. 1, G, H and J), and secretion of mature IL-1β relied on caspase-1 and correlated well with the observed speck response (Fig. 1, H and J).

While Tal did not trigger any cell death, UV caused substantial cell death as quantified by lactate dehydrogenase (LDH) release or uptake of the non-cell permeable DNA dye DRAQ7 (Fig. S1, G, H and I). In line with the relatively weak inflammasome responses to UV, knockout of NLRP1 did not alter cell death. Yet, UV-induced cell death was completely blocked by pan-caspase inhibitor Z-VAD(OMe)-FMK (Z-VAD; Fig. S1 I). We further observed apoptosis-related cleavage of caspase-3 and poly (ADP-ribose) polymerase-1 (PARP) in immunoblots (Fig. S4, A and B), which was also independent of NLRP1. We thus concluded that NLRP1 activation by UV irradiation did not dominate the cell death response, but strongly promotes inflammation by inflammasome-mediated IL-1β release.

In contrast to HEK^NLRP1+ASC^ cells, N/TERT-1^C1C-EGFP^ assembled inflammasomes and secreted IL-1β in a caspase-1–dependent fashion when transfected with dsRNA, which was not observed in NLRP1 knockout cells (Fig. 1 K). This suggests that additional factors absent in HEK 293T cells contributed to NLRP1 activation. Transfection of poly(dA:dT), but not HT-DNA, induced minor inflammasome activation and IL-1β release, but this was independent of NLRP1. As for UV, we observed LDH release after transfection of dsRNA and DNA, but found cell death to be independent of NLRP1 (Fig. S1 J). No ASC specks or IL-1β secretion were detected in response to ATP depletion in keratinocytes, although it cannot be ruled out that this is caused by the rapid cell death or cell-type-specific differences (Fig. S1 K).

In summary, the established HEK^NLRP1+ASC^ and N/TERT-1^C1C-EGFP^ reporter cells allow robust detection of NLRP1 inflammasome assembly combined with simple quantification of ASC specks by flow cytometry. This makes them a useful tool to screen for potential NLRP1 activators and reveal the mechanisms of NLRP1 activation.

### UV treatment activates NLRP1 inflammasomes through p38 kinase activity

**Figure 2.**
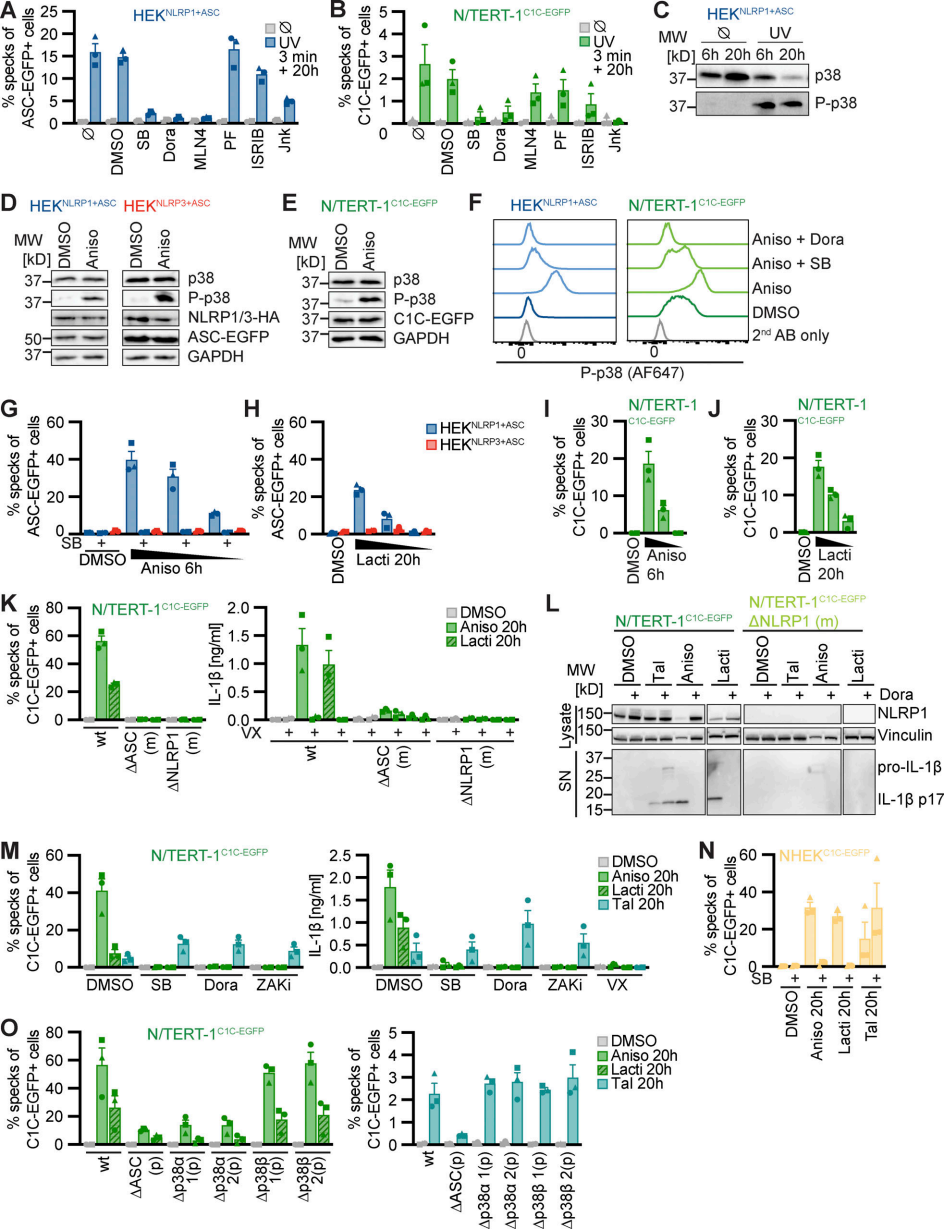
**Human NLRP1 is activated by the ribotoxic stress response. (A–C)** HEK^NLRP1+ASC^ (A and C) or N/TERT-1^C1C-EGFP^ (B) cells were treated with UV for 3 min and cultivated for 20 h in the presence of 20 μM SB, 10 µM Dora, 1 μM MLN4, 1 μM PF, 200 nM ISRIB, 3 µM Jnk, or DMSO. ASC speck formation was quantified by flow cytometry as described in Fig. 1 (A and B), or lysates analyzed by immunoblot with antibodies for p38 and phospho-p38 (P-p38; C). **(D–F)** HEK^NLRP1+ASC^ (D and F), HEK^NLRP3+ASC^ (D), or N/TERT-1^C1C-EGFP^ (E and F) were treated with DMSO or 15 µM Aniso for 60 min, where indicated in the presence of 20 µM SB or 10 µM Dora. Lysates were analyzed by immunoblot with antibodies for p38, P-p38, HA, EGFP, or GAPDH (D and E). Fixed cells were stained for P-p38 and analyzed by flow cytometry (F). **(G–J)** HEK^NLRP1+ASC^, HEK^NLRP3+ASC^ (G and H) or N/TERT-1^C1C-EGFP^ (I and J) cells were treated with DMSO, 15/1.5/0.15 µM Aniso for 6 h, or 2.5/0.5/0.1 µM Lacti for 20 h, where indicated in the presence of 20 µM SB. Specks were quantified as in A and B. **(K–O)** N/TERT-1^C1C-EGFP^ cells (K, L, M, and O), their monoclonal NLRP1 or ASC knockout derivatives (K and L), their polyclonal p38α or p38β knockout derivatives (O), or NHEK^C1C-EGFP^ (N) were treated with DMSO, 15 µM Aniso, 2 µM Lacti, or 30 µM Tal for 20 h, where indicated in the presence of 20 µM SB, 10 µM Dora, or 100 nM ZAKα inhibitor 6p (ZAKi). Specks were quantified as in A and B. IL-1β from the supernatants of cells stimulated in the absence or presence of 100 μM VX was quantified by HTRF (K and M). NLRP1 and Vinculin in the lysates, as well as IL-1β in precipitated supernatants were analyzed by immunoblot (L). The immunblot data from the complete experiment with additional samples and antibodies is shown in Fig. S4. N/TERT-1^C1C-EGFP^ and NHEK^C1C-EGFP^ cells were stimulated in the presence of 100 µM VX for all flow cytometry experiments. Data from all experiments quantifying specks or IL-1β release represents average values (with individual data points) from three independent experiments ± SEM. Immunoblots in C–E and L, as well as flow cytometry data in F display results representative of two (L) or three (C–F) independent experiments. MW, molecular weight. Source data are available for this figure: SourceData F2.

**Figure S2.**
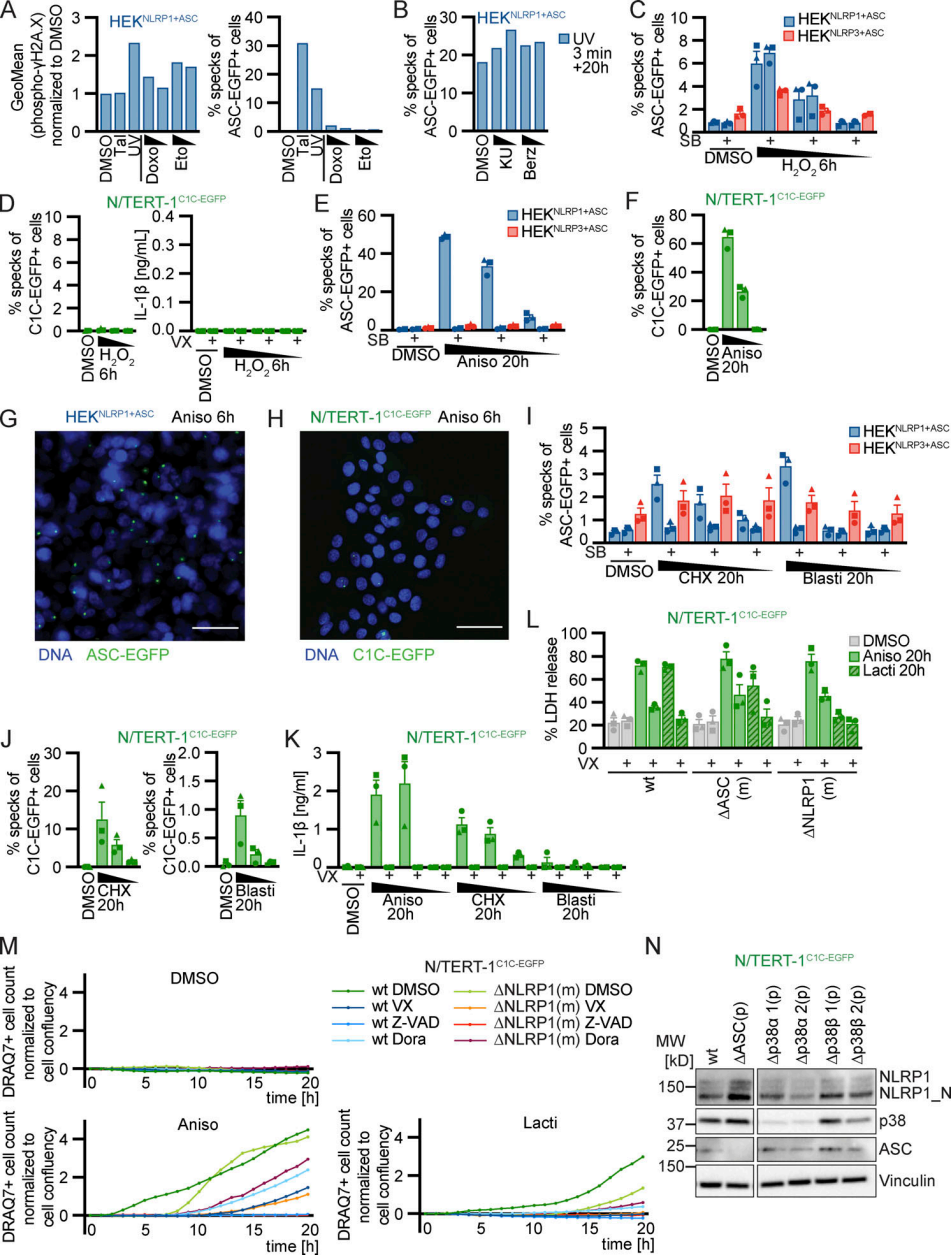
**Human NLRP1 is activated by the ribotoxic stress response. (A)** HEK^NLRP1+ASC^ cells were stimulated with 30 µM Tal, UV for 3 min, 20/2 µM Doxo, or 100/25 µM Eto and harvested after 20 h. Cells were stained for phospho-γH2A.X and analyzed for DNA damage markers (left) or ASC-EGFP specking (right) by flow cytometry as in Fig. 1. **(B)** HEK^NLRP1+ASC^ cells were stimulated with UV for 3 min and subsequently cultivated in the presence of 10/1 µM KU-60019 (KU), or 1/0.1 µM Berzosertib (Berz) for 20 h, followed by quantification of ASC specks as in A. **(C and D)** HEK^NLRP1+ASC^, HEK^NLRP3+ASC^ (C), or N/TERT-1^C1C-EGFP^ (D) cells were treated with 1.5/0.3/0.06 mM H_2_O_2_ for 6 h, where indicated in the presence of 20 μM SB or 100 μM VX. Specks and secreted IL-1β were quantified by flow cytometry and HTRF, respectively. **(E and F)** HEK^NLRP1+ASC^, HEK^NLRP3+ASC^ (E), or N/TERT-1^C1C-EGFP^ (F) cells were treated with DMSO or 15/1.5/0.15 µM Aniso for 20 h, where indicated in the presence of 20 µM SB. Specks were quantified as in A. **(G and H)** HEK^NLRP1+ASC^ (G) or N/TERT-1^C1C-EGFP^ (H) cells were seeded on cover slips and stimulated with DMSO or 15 µM Aniso for 6 h as in (Fig. 2, G and I). Fixed cells were stained for DNA and representative images were recorded by wide-field fluorescence microscopy. Scale bars represent 50 μm. **(I–K)** HEK^NLRP1+ASC^, HEK^NLRP3+ASC^ (I), or N/TERT-1^C1C-EGFP^ (J and K) cells were treated with DMSO, 15/1.5/0.15 µM Aniso (K), 1,000/200/40 µM CHX (I–K), or 40/4/0.8 µg/ml Blasti (I–K) for 20 h where indicated in the presence of 20 μM SB or 100 μM VX. Specks and secreted IL-1β were quantified by flow cytometry and HTRF. ASC speck assembly after Aniso treatment shown in F was done in the same experiment. **(L and M)** N/TERT-1^C1C-EGFP^ cells and their monoclonal ASC or NLRP1 knockout derivatives were stimulated with DMSO, 15 µM Aniso, or 2 µM Lacti for 20 h as in Fig. 2 K, where indicated in the presence of DMSO, 100 μM VX, 50 μM Z-VAD, or 10 μM Dora. Cell death was quantified by detection of LDH release (L) or uptake of non-cell permeable DNA dye DRAQ7 over 20 h (M). For LDH detection, the same supernatants as for IL-1β detection in Fig. 2 K were used. **(N)** Lysates of N/TERT-1^C1C-EGFP^ or its polyclonal ASC, p38α, or p38β knockout derivatives were analyzed by immunoblot with the indicated antibodies. N/TERT-1^C1C-EGFP^ cells were stimulated in the presence of 100 µM VX for all flow cytometry experiments. Data from all experiments quantifying specks, LDH release, or IL-1β release represents average values (with individual data points) from one or three independent experiments ± SEM. Microscopy images in G and H, quantifications of DRAQ7 uptake over time in M and immunoblots in N display experiment representatives of two or three independent experiments. MW, molecular weight. Source data are available for this figure: SourceData FS2.

We next followed up on NLRP1 activation by UV, as this is a relevant environmental trigger of inflammation. Human keratinocytes are regularly exposed to UVB, and UV-triggered activation of NLRP1 was well recapitulated in HEK 293T and N/TERT-1 keratinocyte-based reporter cells (Fig. 1 C and Fig. 2 E). UV irradiation damages multiple molecules in the cell directly or indirectly. This includes (1) DNA damage and activation of distinct signaling pathways including kinases ataxia telangiectasia-mutated (ATM) and ATM and Rad3-related (ATR), (2) RNA damage inducing the ribotoxic stress response, as well as (3) the generation of ROS modifying multiple molecule classes. Induction of DNA damage in HEK^NLRP1+ASC^ reporter cells with doxorubicin (Doxo) and etoposide (Eto) did not initiate inflammasome assembly beyond background levels, although phosphorylation of γH2AX as a readout for ATM activation could be verified by flow cytometry (Fig. S2 A). Likewise, inhibition of ATM or ATR did not impair UV-induced NLRP1 activation (Fig. S2 B). While H_2_O_2_ treatment caused NLRP1 inflammasome activation in HEK^NLRP1+ASC^ cells, and even some NLRP3-dependent specks in HEK^NLRP3+ASC^ (Fig. S2 C), no H_2_O_2_-mediated inflammasome activation or IL-1β secretion was observed in N/TERT-1^C1C-EGFP^ (Fig. S2 D). This suggested that ROS were not sufficient for the NLRP1 inflammasome activation observed in both cell types.

UV treatment activates the ribotoxic stress response by damaging cellular RNA. This includes damage of ribosomal RNAs and mRNAs that results in stalled or clashing ribosomes. This response initiates MAPK signaling by activating ZAKα, a splice variant of MAP kinase kinase kinase (MAP3K) ZAK, also known as MAP3K20 (Vind et al., 2020b; Wang et al., 2005; Wu et al., 2020). ZAKα phosphorylates MAP kinase kinases (MAP2K) MKK3 (MAP2K3) as well as MKK6 (MAP2K6; Vind et al., 2020a). These, in turn, activate the four p38 isoforms p38α (MAPK14), p38β (MAPK11), p38γ (MAPK12), and p38δ (MAPK13) by phosphorylation (Canovas and Nebreda, 2021). To test whether UV-induced inflammasome assembly relied on the ribotoxic stress response and p38 signaling, we quantified speck formation in HEK^NLRP1+ASC^ and N/TERT-1^C1C-EGFP^ cells treated with the p38α/β inhibitor SB202190 (SB), or the pan-p38 inhibitor doramapimod (Dora; Fig. 2, A and B). Assembly of ASC specks was completely abrogated by both inhibitors, suggesting that UV-induced NLRP1 activation was indeed critically dependent on p38 kinase activity. In line with that, we observed robust phosphorylation of p38 after UV irradiation (Fig. 2 C). Inflammasome assembly was not sensitive to ISRIB, an inhibitor of the integrated stress response, and only partially affected by Jnk-In-8 (Jnk), a reversible inhibitor of JNK1, JNK2, and JNK3. No changes in NLRP1 activation were observed when we used PF3644022 (PF) to inhibit MAPKAPK2 (MK2), an effector kinase directly phosphorylated by p38. Among other activities, MK2 regulates the stability of mRNA transcripts. Our data thus indicate that changes in mRNA stability by MK2 were not involved in NLRP1 inflammasome activation, although p38-dependent gene regulation could not be ruled out.

### The ribotoxic stress response triggers NLRP1 activation

We next evaluated whether other activators of the ribotoxic stress response were also able to activate NLRP1 inflammasomes. We first tested anisomycin (Aniso), an antibiotic produced by *Streptomyces griseolus*, which inhibits protein synthesis by interfering with peptide bond formation. Aniso is one of the strongest activators of ZAKα phosphorylation and the ribotoxic stress response, resulting in rapid p38 phosphorylation (Vind et al., 2020b). We confirmed robust phosphorylation and thus activation of p38 in HEK 293T cells and keratinocytes after Aniso treatment by immunoblot and flow cytometry with phospho-p38-specific antibodies, suggesting that the ribotoxic stress response is functional in HEK 293T cells and N/TERT-1 keratinocytes (Fig. 2, D–F). Aniso treatment activated NLRP1 inflammasomes in HEK^NLRP1+ASC^ and N/TERT-1^C1C-EGFP^ cells in a dose-dependent manner (Fig. 2, G and I; and Fig. S2, E and F). The fraction of responding cells was comparable to Tal in HEK-based systems and substantially stronger than Tal in keratinocytes, with more than 15% of the cells assembling ASC specks already after 6 h (Videos 1, 2, and 3; and Fig. S2, G and H).

**Video 1.** **Human NLRP1 is activated by the ribotoxic stress response.** N/TERT-1^C1C-EGFP^ cells in the presence of PI were treated with DMSO and recorded by wide-field microscopy over time. C1C-EGFP is displayed in green, PI signal in red, and phase contrast in grayscale. The time after stimulation is shown as hh:mm; the frame rate is 7 fps.

**Video 2.** **Human NLRP1 is activated by the ribotoxic stress response.** N/TERT-1^C1C-EGFP^ cells in the presence of PI were treated with 30 µM Tal and recorded by wide-field microscopy over time; data is displayed as described in Video 1.

**Video 3.** **Human NLRP1 is activated by the ribotoxic stress response.** N/TERT-1^C1C-EGFP^ cells in the presence of PI were treated with 15 µM Aniso and recorded by wide-field microscopy over time; data is displayed as described in Video 1.

Next, we tested lactimidomycin (Lacti) from *Streptomyces amphibiosporus* and cycloheximide (CHX), which both inhibit ribosomes by interfering with polypeptide translocation. Lacti was found to be a stronger activator of the ribotoxic stress response than CHX (Vind et al., 2020b). Both Lacti and CHX activated NLRP1 inflammasomes in a dose-dependent manner in HEK cells and keratinocytes (Video 4; and Fig. 2, H and J; and Fig. S2, I and J), with a stronger response to Lacti. In comparison with Aniso, the Lacti-induced NLRP1 response was weaker and started later in both reporter cells.

**Video 4.** **Human NLRP1 is activated by the ribotoxic stress response.** N/TERT-1^C1C-EGFP^ cells in the presence of PI were treated with 2 µM Lacti and recorded by wide-field microscopy over time; data is displayed as described in Video 1.

In the course of our efforts to generate NLRP1 reporter cell lines, we had observed that Blasticidin S (Blasti) also induces a low level of NLRP1 inflammasome assembly. Blasti impairs termination of translation at the ribosome. We found that Blasti induces weak NLRP1 inflammasome assembly at the highest dose in HEK cells and keratinocytes (Fig. S2, I and J). All ribotoxic stress stimuli that induced a robust ASC speck response in the N/TERT-1^C1C-EGFP^ cells (Aniso, Lacti, CHX) also triggered caspase-1–dependent IL-1β secretion (Fig. 2 K and Fig. S2 K).

Further analyses were conducted with the stronger NLRP1 activators Aniso and Lacti. For both, ASC speck assembly depended on NLRP1 and ASC, as neither HEK^NLRP3+ASC^ cells (Fig. 2, G and H) nor NLRP1 or ASC knockout cell lines of N/TERT-1^C1C-EGFP^ assembled inflammasomes in response to both stimuli (Fig. 2 K). Both triggers initiated robust IL-1β secretion by keratinocytes. In agreement with ASC speck response, Aniso- and Lacti-treated NLRP1 and ASC knockout keratinocytes did not secrete any IL-1β (Fig. 2 K). Processing of caspase-1 and maturation of IL-1β by both triggers were confirmed by immunoblot (Fig. 2 L; and Fig. S4, A and B).

Both antibiotics induced strong release of LDH (Fig. S2 L) and uptake of DNA dye DRAQ7 (Fig. S2 M). In case of Lacti, cell death was largely abrogated in the absence of NLRP1, while cell death initiated by Aniso was only delayed. DRAQ7 uptake in response to both triggers was completely blocked by the pan-caspase inhibitor Z-VAD (Fig. S2 M). Both stimuli further led to processing of caspase-3 and cleavage of the caspase-3 substrate PARP as revealed by immunoblot (Fig. S4, A and B), indicating the initiation of apoptosis as described before (Liu et al., 2013; Seo et al., 2013), possibly followed by secondary necrosis that compromised integrity of the plasma membrane. Cell death and apoptosis were largely prevented by p38 inhibitor Dora, suggesting that apoptosis and cell death were additional consequences of p38 MAPK signaling.

To test whether NLRP1 activation by Aniso and Lacti was indeed initiated by the ribotoxic stress response, we quantified inflammasome assembly in the presence of p38 inhibitors SB and Dora (Fig. 2 M). We also tested compound 6p (ZAKi), an inhibitor of the upstream MAP3K ZAKα (Yang et al., 2020). Inflammasome assembly and IL-1β secretion were completely blocked by both p38 inhibitors as well as by ZAKi. This was not the case for Tal-induced NLRP1 responses, which were even slightly boosted by some of the inhibitors. We also confirmed that NLRP1 activation by Aniso and Lacti in primary keratinocytes expressing C1C-EGFP was sensitive to p38 inhibitors (Fig. 2 N).

To evaluate the role of the different p38 isoforms in the activation of NLRP1 inflammasomes, we generated polyclonal knockout cell lines of p38α and p38β in the background of N/TERT-1^C1C-EGFP^ and compared these to polyclonal ASC knockouts (expecting similar knockout efficiencies; Fig. S2 N). Knockout of p38α inhibited Aniso- and Lacti-induced NLRP1 speck assembly to similar levels as ASC knockouts, while knockouts of p38β did not impair inflammasome assembly (Fig. 2 O). We next evaluated p38 levels by immunoblot using a p38 antibody recognizing p38α, β, and γ isoforms. P38 signals were substantially diminished in p38α knockout keratinocytes, suggesting that p38α is the most abundant isoform in keratinocytes (Fig. S2 N). NLRP1 inflammasome assembly triggered by Tal treatment was not impaired in p38α knockouts, coherent with the p38 inhibitor experiments. This suggests that activation of NLRP1 by the ribotoxic stress response follows a mechanism independent of Tal-mediated release of DPP9 inhibition.

**Figure 3.**
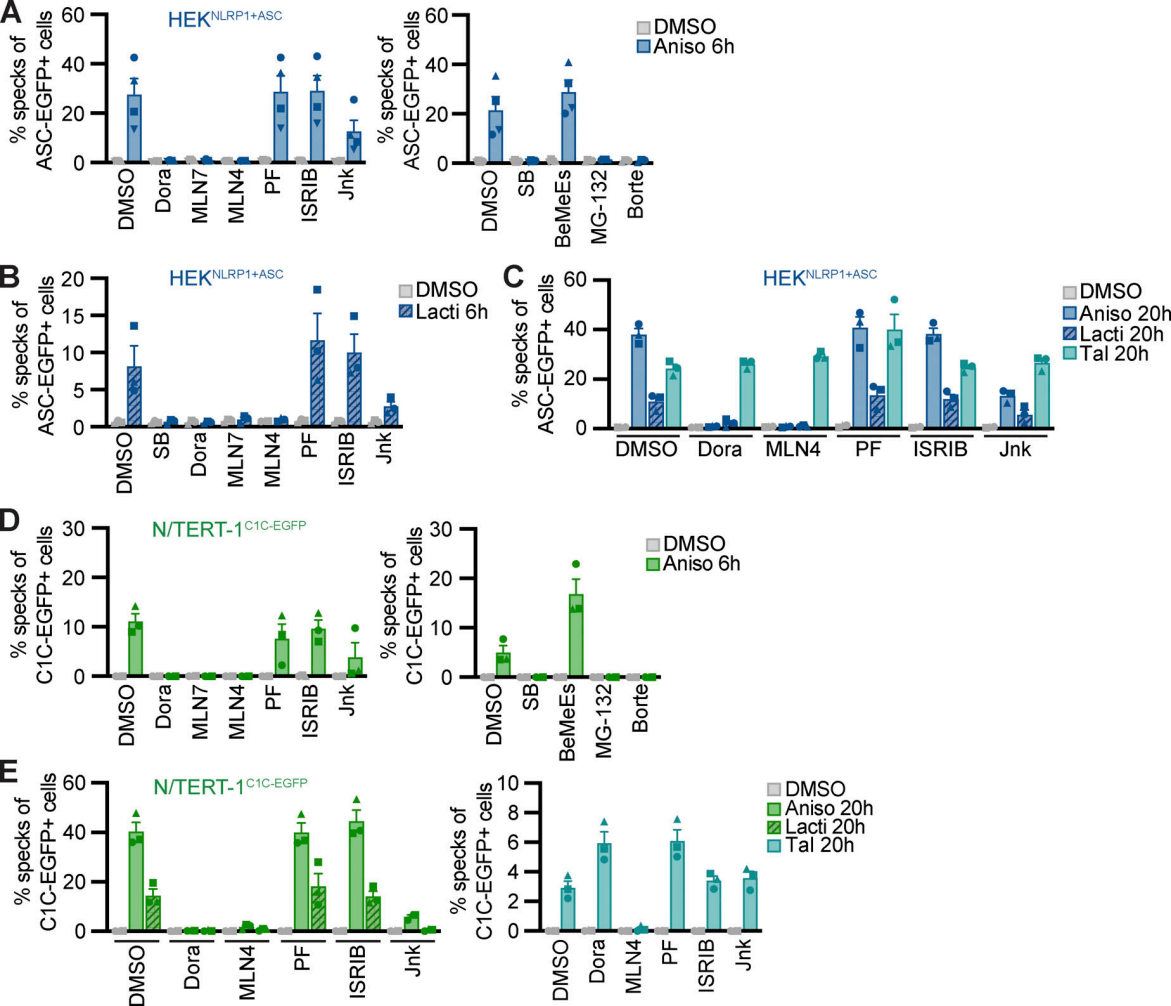
**NLRP1 activation by ribotoxic stress response relies on the ubiquitination machinery and proteasomes. (A–E)** HEK^NLRP1+ASC^ (A–C) or N/TERT-1^C1C-EGFP^ cells (D and E) were stimulated with DMSO, 15 µM Aniso (A, C, D, and E), 2 µM Lacti (B, C, and E), or 30 µM Tal (C and E) for the indicated time. Stimulation was performed in the presence of 10 µM Dora, 1 μM MLN7, 1 μM MLN4, 1 μM PF, 200 nM ISRIB, 3 µM Jnk, 20 µM SB, 20 µM BeMeEs, 1 µM MG-132, 1 µM Borte, or DMSO as indicated. N/TERT-1 cells were always stimulated in the presence of 100 µM VX. Specking cells were quantified by flow cytometry as described in Fig. 1. Data represents average values (with individual data points) from three or four independent experiments ± SEM.

**Figure S3.**
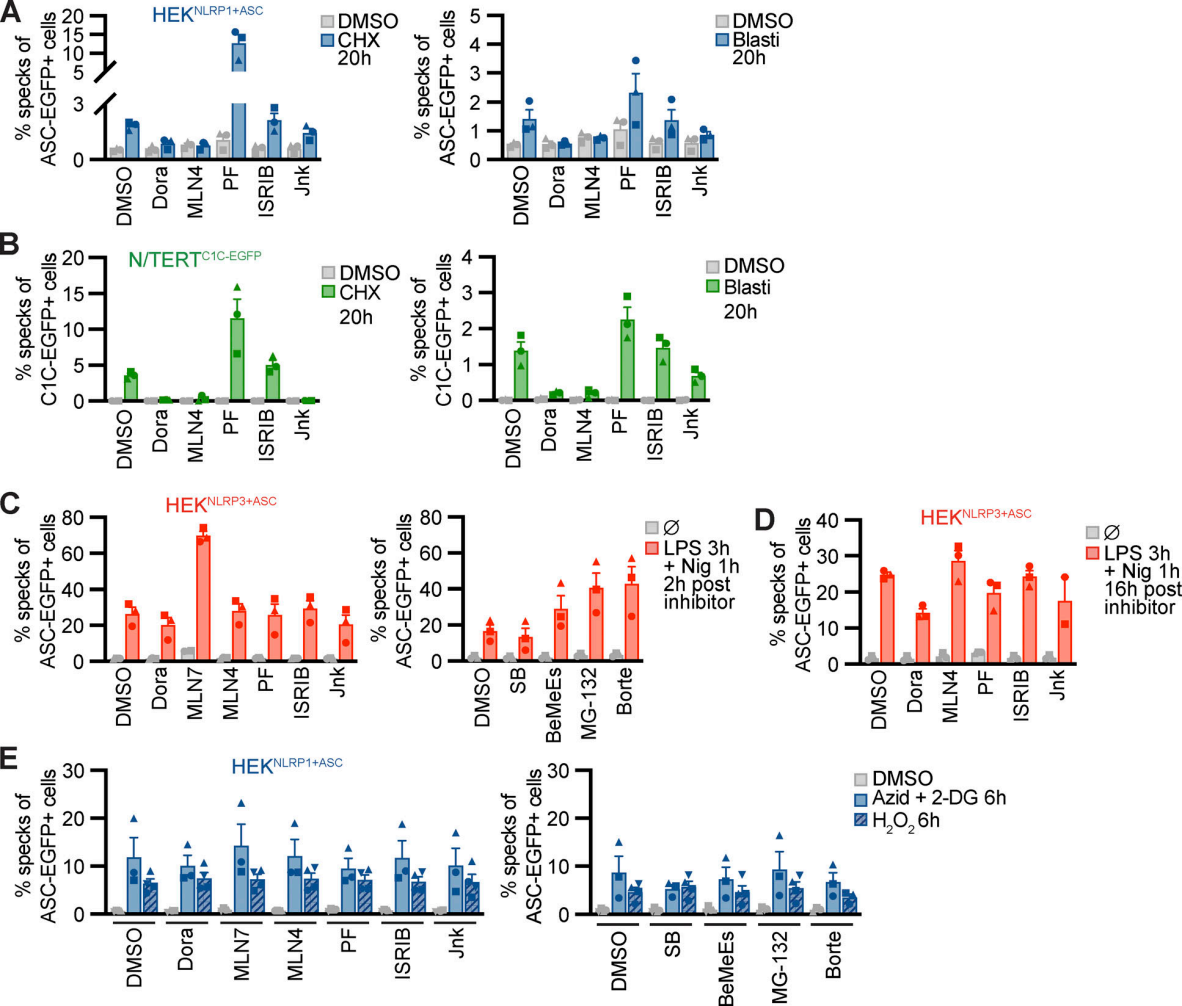
**NLRP1 activation by ribotoxic stress response relies on the ubiquitination machinery and proteasomes. (A–E)** HEK^NLRP1+ASC^ (A and E), N/TERT-1^C1C-EGFP^ (B), or HEK^NLRP3+ASC^ (C and D) were stimulated with DMSO, 1 mM CHX (A and B), 20 µg/ml Blasti (A and B), 200 ng/ml LPS followed by 10 µM Nig (C and D), 10 mM azide and 50 mM 2-DG (E), or 1.5 mM H_2_O_2_ (E) for the indicated times. Cells were stimulated in the presence of 10 µM Dora, 1 μM MLN4, 1 μM PF, 200 nM ISRIB, 3 µM Jnk, 1 μM MLN7, 20 μM SB, 20 µM BeMeEs, 1 µM MG-132, 1 µM Borte, or DMSO as indicated. Note that LPS + Nig stimulation was performed towards the end of inhibitor treatment to evaluate NLRP3 responses after 6 h (C) or 20 h (D) in the presence of the indicated inhibitors. Experiments were done in parallel to data shown in Fig. 3, A and C. Specking cells were quantified by flow cytometry as in Fig. 1, and N/TERT-1 cells were always stimulated in the presence of 100 µM VX. Data represents average values (with individual data points) from three or four independent experiments ± SEM.

Many known activators of human NLRP1 and murine Nlrp1b require activity of the proteasome (Gai et al., 2019; Johnson et al., 2018; Sandstrom et al., 2019; Bauernfried et al., 2021). Other cellular factors such as components of the N-end rule pathway are only required for distinct triggers, such as anthrax toxin lethal factor as an activator of Nlrp1b, but not for activation by Tal (Chui et al., 2019). To define the cellular activities required for NLRP1 activation by the ribotoxic stress response, we quantified inflammasome responses in the presence of different inhibitors in HEK^NLRP1+ASC^ and N/TERT-1^C1C-EGFP^ cells (Figs. 3 and S3). As before, activation of NLRP1 with Aniso, Lacti, CHX, and Blasti was consistently inhibited by p38 inhibitors SB and Dora (Figs. 2, G and L, S2, E and I, 3, A–E; and Fig. S3, A and B). To analyze the role of proteasomal degradation, we utilized the proteasome inhibitors MG-132 and bortezomib (Borte), as well as the E1 ubiquitin activating enzyme inhibitor MLN7243 (MLN7) for 6 h activation experiments, as these inhibitors are toxic during prolonged incubations. Cullin-based ubiquitin ligase complexes are critically dependent on modification with the ubiquitin-like molecule NEDD8 (Soucy et al., 2009). For 20 h treatments, we thus used the less toxic NEDD8 activating enzyme inhibitor MLN4924 (MLN4), allowing us to study the influence of cullin ubiquitin ligase activity. NLRP1 activation by the ribotoxic stress response was consistently shut down by all mentioned inhibitors of proteasomal degradation (Fig. 3, A–E; and Fig. S3, A and B), implying that NLRP1 degradation, involving E1 enzymes, cullin ubiquitin ligases, and the proteasome, is necessary to activate NLRP1 after ribotoxic stress. Inhibition of MAPKAP2 (PF), the integrated stress response (ISRIB), or the N-end rule pathway (bestatin methyl ester [BeMeEs]) did not inhibit NLRP1 activation by either activator. Inhibition of JNK kinases (Jnk) only partially affected NLRP1 activation. Importantly, inhibition of p38, neddylation, E1, or the proteasome for the same duration of time did not inhibit NLRP3 activation by LPS and Nig in HEK^NLRP3+ASC^ (Fig. S3, C and D); E1 and proteasome inhibitors even enhanced NLRP3 responses. This indicates that the requirement for these cellular activities is specific for NLRP1, and not inflammasomes in general. As before, activation of NLRP1 by Tal (Fig. 3, C and E) did not require p38 activity. Thus, p38 is not required to render NLRP1 functional per se.

Activation of NLRP1 by ATP depletion or H_2_O_2_ in HEK^NLRP1+ASC^ was not sensitive to inhibitors of p38 activity, E1 enzymes, neddylation, or the proteasome, suggesting yet another mechanism of activation independent of the ribotoxic stress response (Fig. S3 E).

Taken together, we find that diverse activators of the ribotoxic stress response activate human NLRP1. This response is distinct from NLRP1 activation by inhibition of DPP9 as it depends on p38 kinase activity. As several of the described NLRP1 activators efficiently inhibit translation, NLRP1 activation does not depend on p38-dependent gene expression, but rather on the kinase activity of p38 directly. As described for other NLRP1 stimuli, NLRP1 activation through the ribotoxic stress response required E1 activity, neddylation, and proteasome activity, indicating that these cellular functions are required for most mechanisms of NLRP1 inflammasome activation (with the notable exception of ATP depletion and ROS). The ubiquitin-proteasome pathway likely contributes to the N-terminal degradation of NLRP1 itself, followed by the release of the C-terminal NLRP1^UPA-CARD^.

### Alphavirus-induced NLRP1 activation is also dependent on p38 kinase activity

**Figure 4.**
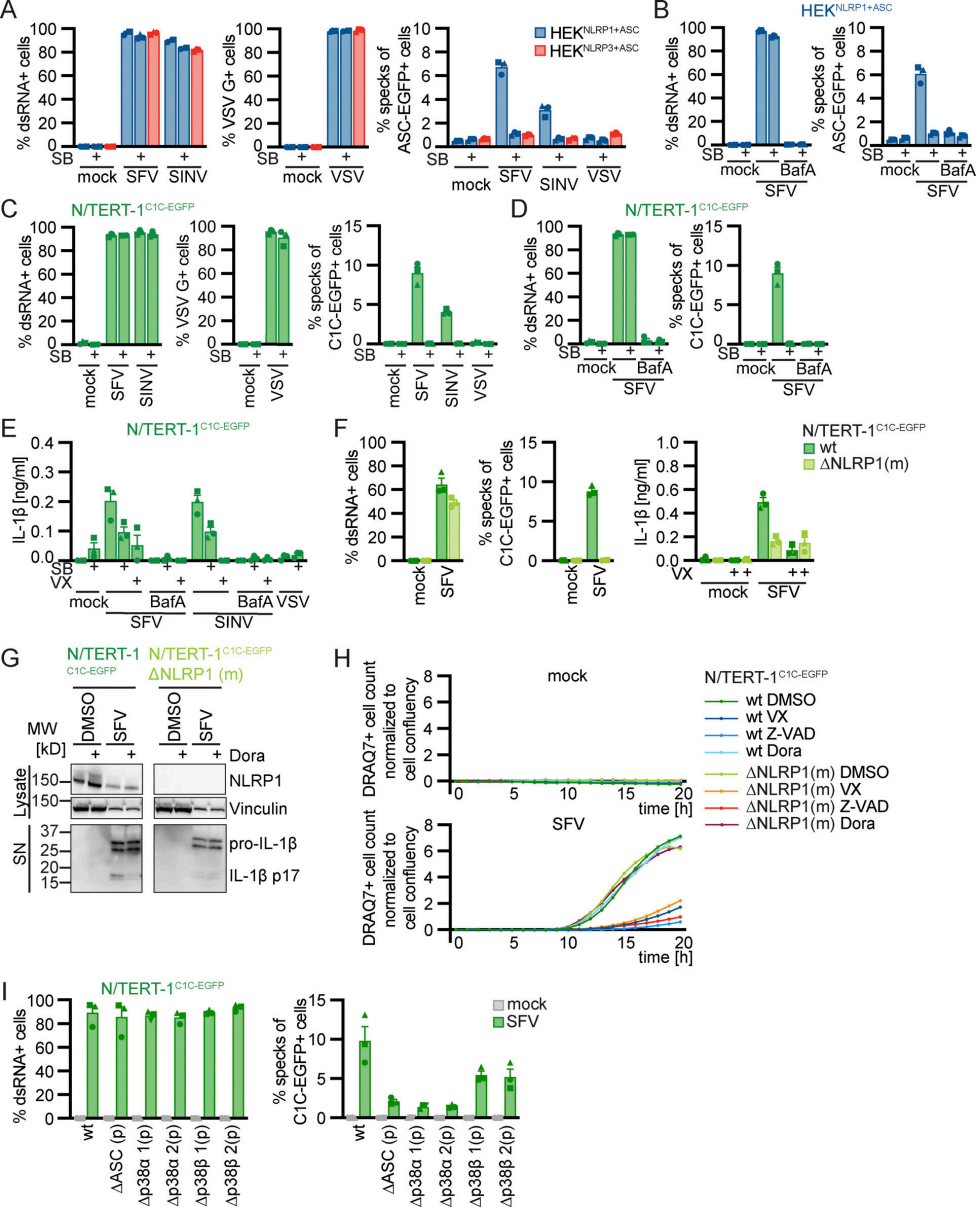
**Alphavirus infection activates human NLRP1 in a p38-dependent manner. (A–E)** HEK^NLRP1+ASC^ and HEK^NLRP3+ASC^ (A and B) or N/TERT-1^C1C-EGFP^ (C–E) cells were infected with SFV, SINV, or VSV at an MOI of 1 (HEK) or 5 (N/TERT-1) for 20 h in the presence or absence of 20 µM SB, where indicated in the presence of 100 nM BafA. Infected cells were stained with antibodies for dsRNA (SFV, SINV) or VSV G (VSV), and cells analyzed by flow cytometry. Speck assembly was quantified by flow cytometry as described in Fig. 1 (A–D). Specks were only quantified in infected cells for all samples treated with virus, i.e., not in mock controls. IL-1β from the supernatants of cells stimulated in the absence or presence of 100 μM VX was quantified by HTRF (E). **(F–I)** N/TERT-1^C1C-EGFP^ cells (F–I), their monoclonal NLRP1 knockout derivative (F–H), or their polyclonal p38α or p38β knockout derivatives (I) were infected with SFV as in C–E. Infection, speck assembly (F, I) and IL-1β release (F) were quantified as in C–E. NLRP1 and Vinculin in the lysates, as well as IL-1β in precipitated supernatants were analyzed by immunoblot (G). The immunblot data from the complete experiment with additional samples and antibodies is shown in Fig. S4. Uptake of non-cell permeable DNA dye DRAQ7 was detected every hour for a total of 20 h, in the presence of DMSO, 100 μM VX, 50 μM Z-VAD, or 10 µM Dora (H). N/TERT-1^C1C-EGFP^ cells were stimulated in the presence of 100 µM VX for all flow cytometry experiments. Data from all experiments quantifying specks or IL-1β release represents average values (with individual data points) from three independent experiments ± SEM. Immunoblots (G) and quantifications of DRAQ7 uptake over time (H) display data representative of two (G) or three (H) independent experiments. MW, molecular weight. Source data are available for this figure: SourceData F4.

**Figure S4.**
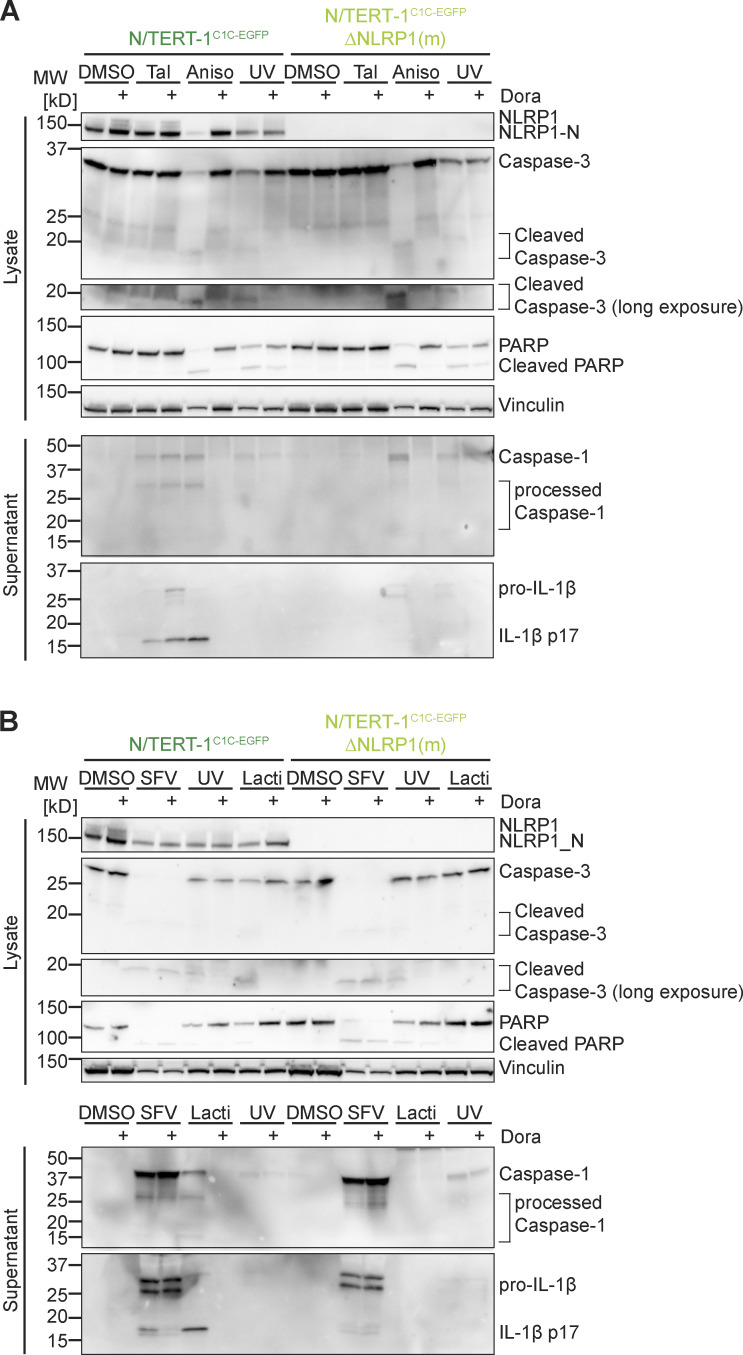
**Supplementary immunoblots for Figs. 1, 2, and 4. (A and B)** N/TERT-1^C1C-EGFP^ or monoclonal NLRP1 knockout N/TERT-1^C1C-^EGFP cells were stimulated with DMSO, 30 μM Tal (A), 15 µM Aniso (A), UV for 3 min (A, B), 2 µM Lacti (B), or infected with SFV at an MOI of 5 (B) for 20 h. Stimulation was performed in the presence or absence of 10 µM Dora. Lysates and precipitated supernatants were analyzed by immunoblot with antibodies for NLRP1, caspase-3, PARP, caspase-1, IL-1β, and Vinculin. Please note that the order of samples is different for lysates and supernatants in B. Excerpts of these immunoblots are displayed in Figs. 2 and 4. Data displays experiment representatives of two independent experiments. MW, molecular weight. Source data are available for this figure: SourceData FS4.

Infection of human keratinocytes with the model alphavirus SFV as well as cytosolic delivery of dsRNA was reported to activate NLRP1 inflammasomes by direct binding of dsRNA to NLRP1 (Bauernfried et al., 2021). To study NLRP1 activation in response to viral infection, we thus infected HEK^NLRP1+ASC^, HEK^NLRP3+ASC^, and N/TERT-1^C1C-EGFP^ cells with SFV, the closely related Sindbis virus (SINV), as well as vesicular stomatitis virus (VSV), a negative-sense single-stranded RNA virus. We quantified infection by staining for dsRNA (SFV, SINV) or the VSV G protein, and only included infected cells in the flow cytometry analysis of inflammasome activation. Nearly all of the cells were infected with the respective viruses, but only SFV and SINV induced a robust assembly of ASC specks in HEK^NLRP1+ASC^ and N/TERT-1^C1C-EGFP^ cells 20 h after infection (Fig. 4, A and C). The response in the HEK-based system was weak compared to Tal, while keratinocytes exhibited robust speck formation. SFV was the stronger inflammasome activator in both reporter cell lines and we thus focused further analyses on this alphavirus. Treatment of the cells with the V-ATPase inhibitor bafilomycin A1 (BafA) completely abolished SFV infection, as alphaviruses require endosomal acidification to fuse with the limiting membrane of endosomes (Marsh et al., 1983; Fig. 4, B and D). The treatment also abrogated ASC speck formation, demonstrating that cytosolic delivery of viral genomes was critical to activate NLRP1 in the reporter cells. Alphavirus-infected N/TERT-1^C1C-EGFP^ cells released IL-1β in a caspase-1–dependent manner, which was also inhibited by BafA (Fig. 4 E). ASC speck formation and release of IL-1β after SFV infection was abrogated in NLRP1 knockout N/TERT-1^C1C-EGFP^ cells (Fig. 4 F and Fig. S4 B). Processing of caspase-1 and maturation of IL-1β were confirmed by immunoblot (Fig. 4 G and Fig. S4 B). SFV infection also led to caspase-3 and PARP cleavage as well as DRAQ7 uptake, and thus apoptosis with secondary necrosis (Fig. 4 H and Fig. S4 B).

Since we had found that activators of the ribotoxic stress response activate human NLRP1 in a p38-dependent manner, we wondered whether the activation by alphaviruses also depends on p38 kinase activity. ASC speck formation in HEK^NLRP1+ASC^ and N/TERT-1^C1C-EGFP^ cells infected with SFV and SINV was completely inhibited by the p38α/β inhibitor SB (Fig. 4, A and C). In line with that, IL-1β release by alphavirus-infected keratinocytes was substantially reduced by SB and Dora (Fig. 4, E and G). In contrast, inhibition of p38 did not rescue N/TERT-1^C1C-EGFP^ from SFV-induced cell death, and neither were NLRP1 knockout cells (Fig. 4 H). Yet, SFV-induced cell death was sensitive to pan-caspase inhibitors (Fig. 4 H) and cleavage of caspase-3 and PARP detected by immunoblot were unaltered by p38 inhibition (Fig. S4 B). NLRP1-dependent pyroptosis was probably too weak to substantially contribute to the observed cell death after infection with cytotoxic SFV. As for the ribotoxic stress response initiated by UV and Aniso, however, activation of NLRP1 inflammasomes substantially alters the physiological response as it mediates the release of the strong pro-inflammatory cytokine IL-1β.

**Figure 5.**
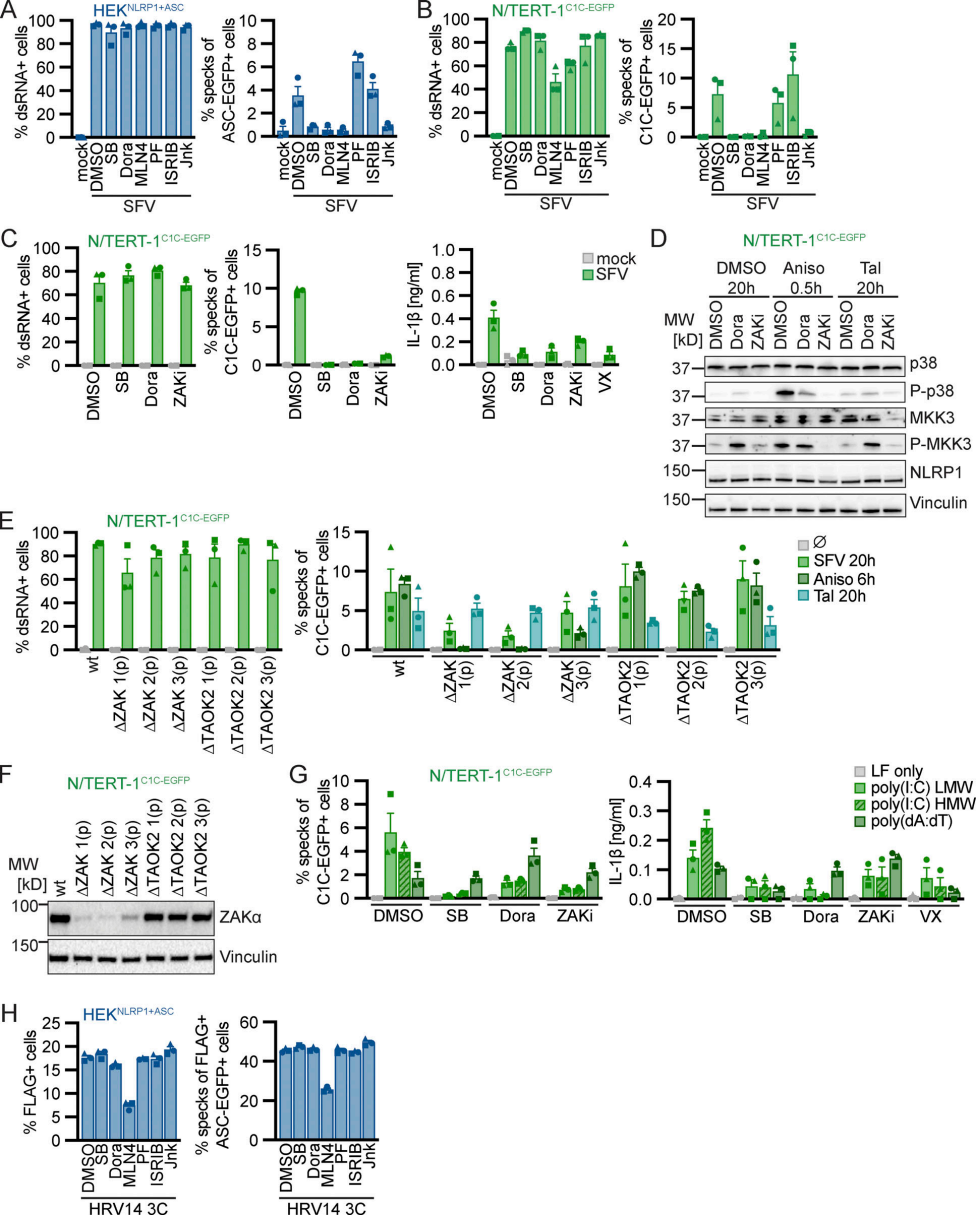
**Alphavirus infection and cytosolic dsRNA activate human NLRP1 in a p38 and ZAKα-dependent manner. (A–C)** HEK^NLRP1+ASC^ (A) or N/TERT-1^C1C-EGFP^ (B and C) cells were infected with SFV at an MOI of 5 for 20 h in the presence of DMSO, 20 µM SB, 10 µM Dora, 1 μM MLN4, 1 μM PF, 200 nM ISRIB, 3 µM Jnk, 100 nM ZAKα inhibitor 6p (ZAKi), or 100 μM VX. Infected cells were stained with antibodies for dsRNA. Infection and speck assembly in infected cells were quantified by flow cytometry as described in Fig. 4. IL-1β from the supernatants of cells was quantified by HTRF (C). **(D)** N/TERT-1^C1C-EGFP^ were treated with DMSO, 15 µM Aniso, or 30 μM Tal for the indicated times in the presence of DMSO, 10 µM Dora, or 100 nM ZAKi. Lysates were analyzed by immunoblot with antibodies for p38, P-p38, MKK3, P-MKK3, NLRP1, and Vinculin. **(E)** N/TERT-1^C1C-EGFP^ cells and their polyclonal ZAKα or TAOK2 knockout derivatives were infected with SFV as in A, or stimulated with DMSO, Aniso, or Tal as in D for the indicated times. Infection and speck assembly in infected, C1C-EGFP–positive cells (SFV), or speck assembly in C1C-EGFP–positive cells (untreated and other triggers) were quantified as before. **(F)** Polyclonal ZAKα or TAOK2 knockout derivatives of N/TERT-1^C1C-EGFP^ cells were analyzed by immunoblot with the indicated antibodies. **(G)** N/TERT-1^C1C-EGFP^ cells were transfected with 1 µg/ml of the indicated nucleic acid species in presence of DMSO, 20 µM SB, 10 µM Dora, 100 nM ZAKi, or 100 μM VX for 20 h. Speck assembly and IL-1β release were quantified as in Fig. 1. **(H)** HEK^NLRP1+ASC^ cells transiently expressing FLAG-tagged HRV14 protease 3C in the presence of 20 μM SB, 10 µM Dora, 1 μM MLN4, 1 μM PF, 200 nM ISRIB, 3 µM Jnk, or DMSO for 20 h were analyzed for FLAG expression and specks as described in Fig. 1. N/TERT-1^C1C-EGFP^ cells were stimulated in the presence of 100 µM VX for all flow cytometry experiments. Data from all experiments quantifying specks or IL-1β release represents average values (with individual data points) from three independent experiments ± SEM. Immunoblots in D and F display data representative of three independent experiments. MW, molecular weight. Source data are available for this figure: SourceData F5.

Inflammasome assembly after SFV infection was inhibited to similar extends in polyclonal knockouts of p38α and ASC in N/TERT-1^C1C-EGFP^ cells (Fig. 4 I). The pan-p38 inhibitor Dora, neddylation inhibitor MLN4, and JNK inhibitor Jnk completely inhibited inflammasome assembly in HEK^NLRP1+ASC^ and N/TERT-1^C1C-EGFP^ cells after infection with SFV (Fig. 5, A and B). As NLRP1 inflammasome activation by alphavirus infection depends on p38 kinase activity and neddylation, virus infection likely activates NLRP1 by a mechanism that resembles activation by the ribotoxic stress response. To test whether NLRP1 activation by alphaviruses engages a similar signaling cascade relying on MAP3K ZAKα, we infected N/TERT-1^C1C-EGFP^ cells with SFV in the presence of p38 or ZAKα inhibitors (Fig. 5 C). While inflammasome assembly was completely abrogated by both p38 inhibitors, ZAK inhibition blocked the formation of ASC specks drastically, but not completely. The same trend was observed for the release of mature IL-1β. To verify the specificity of the employed kinase inhibitors, we next sought to confirm that the pan-p38 inhibitor Dora does not interfere with the Aniso-induced phosphorylation of MAP2K MKK3 (Fig. 5 D). Indeed, immunoblots with phospho-specific antibodies revealed that Dora did not inhibit phosphorylation of MKK3, suggesting that the MAP3K ZAKα is still active in presence of the p38 inhibitor. As expected, inhibition of ZAKα completely blocked Aniso-induced phosphorylation of MKK3 and the downstream kinase p38. We can thus be confident that the complete loss of SFV- and Aniso-induced NLRP1 activation in presence of p38 inhibitors is not caused by indirect interference with ZAKα activity. To further study the contribution of ZAKα, we generated knockouts of ZAK as well as a control MAP3K, TAO kinase 2 (MAP3K17, TAOK2), in the background of N/TERT-1^C1C-EGFP^ cells (Fig. 5, E and F). As expected, NLRP1 activation by Tal was not affected by either of the knockouts (Fig. 5 E). Aniso-induced ASC speck assembly was completely abrogated in ZAK-knockout cells, validating the critical role of ZAKα for the ribotoxic stress response and the resulting p38-dependent NLRP1 activation. In comparison, SFV-induced NLRP1 inflammasome activation was reduced by around 70% in ZAK-knockout cells, but the response was not completely abrogated, resembling the ZAK inhibitor experiments. This suggests that other MAP3K can contribute to p38 activation in the context of alphavirus infections, even though ZAKα seems to play a substantial role in this inflammasome activation pathway.

Double-stranded RNA intermediates of SFV have been proposed to be the relevant trigger for NLRP1 (Bauernfried et al., 2021), and we also observed that N/TERT-1 keratinocytes assemble NLRP1 inflammasomes upon transfection with poly(I:C) (Fig. 1 K). We thus tested whether activation of NLRP1 inflammasome in N/TERT-1^C1C-EGFP^ cells by dsRNA was similarly dependent on p38. We found that poly(I:C)-induced formation of ASC specks and release of IL-1β was strongly decreased by p38 inhibitors as well as the ZAKα inhibitor (Fig. 5 G). In contrast, the NLRP1-independent inflammasome response to poly(dA:dT) was mostly unaffected by the inhibitors. This indicates that cytosolic dsRNA, accumulated during both alphavirus infection and poly(I:C) transfection, initiates NLRP1 activation through a ribotoxic stress response–like pathway involving ZAKα and p38. Further experiments will be required to dissect if alphavirus infection induces bona fide ribotoxic stress, or activates ZAKα differently.

Enteroviral 3C proteases cleave NLRP1 in the linker region between PYD and NACHT domain, and thus induce its N-terminal degradation and inflammasome assembly by NLRP1^UPA-CARD^ (Robinson et al., 2020; Tsu et al., 2021). To test whether NLRP1 activation by enterovirus proteases resembled alphavirus-mediated NLRP1 activation, we tested whether activation by HRV14 3C required p38 activity. Transient overexpression of HRV14 3C protease from a strong promoter in HEK^NLRP1+ASC^ cells triggered inflammasome formation in >40% of transfected cells (Fig. 1 D). Inflammasome assembly was not inhibited by p38 inhibitors (Fig. 5 H). Only neddylation inhibitors reduced ASC speck formation, but also affected expression of the protease. The mechanism of NLRP1 activation by enteroviral proteases was thus clearly distinct from the p38-mediated activation of NRLP1 by alphaviruses.

**Figure 6.**
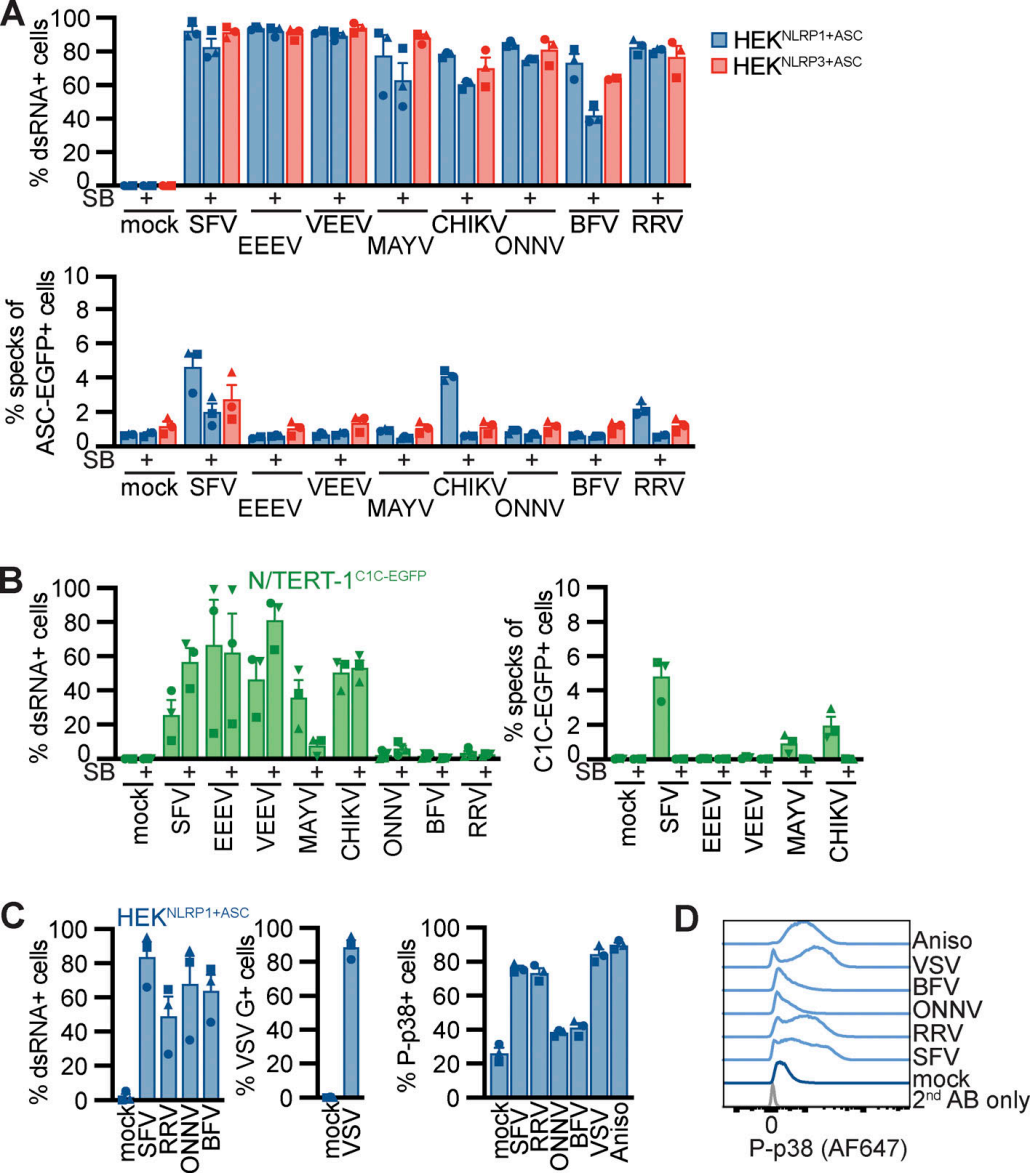
**Alphavirus-induced p38 activity correlates with NLRP1 activation. (A and B)** HEK^NLRP1+ASC^, HEK^NLRP3+ASC^ (A), or N/TERT-1^C1C-EGFP^ (B) cells were infected with SFV (A: MOI 1/B: MOI 5), EEEV (MOI 1/5), VEEV (MOI 1/50), CHIKV (MOI 1/50), MAYV (MOI 25/50), ONNV (MOI 25/50), BFV (MOI 25/50), or RRV (MOI 25/50) for 20 h in the absence or presence of 20 µM SB. Infected cells were stained with antibodies for dsRNA and infection and speck assembly in infected cells were quantified by flow cytometry as in Fig. 4. **(C and D)** HEK^NLRP1+ASC^ cells were infected with the indicated viruses as in A. Control cells were infected with VSV at an MOI of five or treated with 15 µM Aniso. Infected cells were stained with antibodies for dsRNA or VSV G, and P-p38 in infected cells (total cells in case of uninfected samples) was quantified by flow cytometry. Average fractions of P-p38–positive cells from three independent experiments (C) and representative histograms (D) are displayed. N/TERT-1 cells were infected in the presence of 100 µM VX for all flow cytometry experiments. Data represents average values (with individual data points) from three independent experiments ± SEM.

We next tested whether NLRP1 is also important in the detection of alphaviruses that are clinically relevant as human pathogens. We thus infected the HEK and keratinocyte reporter cells with a panel of human pathogenic alphaviruses including Eastern equine encephalitis virus (EEEV), Venezuelan equine encephalitis virus (VEEV), Mayaro virus (MAYV), Chikungunya virus (CHIKV), o’nyong-nyong virus (ONNV), Barmah Forest virus (BFV), and Ross River virus (RRV) in the presence and absence of SB. All tested viruses were able to infect both HEK^NLRP1+ASC^ and HEK^NLRP3+ASC^ cells, as confirmed by staining of dsRNA (Fig. 6 A). In addition to the positive control SFV, we found that CHIKV and RRV activated NLRP1-dependent inflammasomes. ASC assembly in response to all three alphaviruses was inhibited by the p38α/β inhibitor SB. We were not able to infect the N/TERT-1^C1C-EGFP^ cells with ONNV, BFV, and RRV (Fig. 6 B) and the fraction of infected cells was generally more variable for keratinocytes. However, analysis of inflammasome assembly in infected cells revealed clear ASC specking responses in keratinocytes infected with SFV, MAYV, and CHIKV. We cannot rule out that the weak NLRP1 activation in response to MAYV infection was not detected in HEK^NLRP1+ASC^ cells due to the higher background caused by ectopically expressed ASC-EGFP. CHIKV-induced ASC speck assembly in the reporter keratinocytes was also dependent on p38 activity. Since SB strongly decreased MAYV infection, we could not assess whether the weak inflammasome response depends on p38.

It is likely that related alphaviruses expose similar viral molecules in the cytosol and that they hijack similar cellular functions. Yet, only distinct alphaviruses triggered NLRP1 inflammasome assembly. We thus wondered if differential NLRP1 activation correlated to differences in p38 activation. We infected HEK^NLRP1+ASC^ cells with two alphaviruses shown to trigger ASC specks (SFV and RRV) as well as two viruses that did not initiate inflammasome assembly (ONNV and BFV). We detected robust phosphorylation of p38 by flow cytometry in cells infected with SFV and RRV that was comparable to p38 phosphorylation in Aniso-treated cells (Fig. 6 C). Cells infected with ONNV and BFV, however, did not trigger p38 phosphorylation. The ability to activate p38 thus correlated with NLRP1 activation, indicating that MAPK signaling is the critical determinant of NLRP1 activation by alphaviruses. Surprisingly, infection with the control virus VSV induced p38 phosphorylation as well, indicating that either p38 activation by alphaviruses differs from activation by VSV, or that VSV counteracts p38-dependent NLRP1 inflammasome stimulation.

Taken together, we confirmed NLRP1 activation by SFV infection in NLRP1 HEK and keratinocyte reporter cells, but found that inflammasome activation is critically dependent on p38 and mostly dependent on ZAKα activation. It is thus unlikely that direct RNA binding to NLRP1 alone is sufficient for NLRP1 activation. We identified four additional alphaviruses, SINV, RRV, MAYV, and CHIKV, that activate NLRP1, supporting the relevance of NLRP1 as a sensor for the detection of alphavirus in the first organ that these viruses encounter after transmission by insect vectors.

### Activation of NLRP1 by directed ubiquitination does not require p38 kinase activity

**Figure 7.**
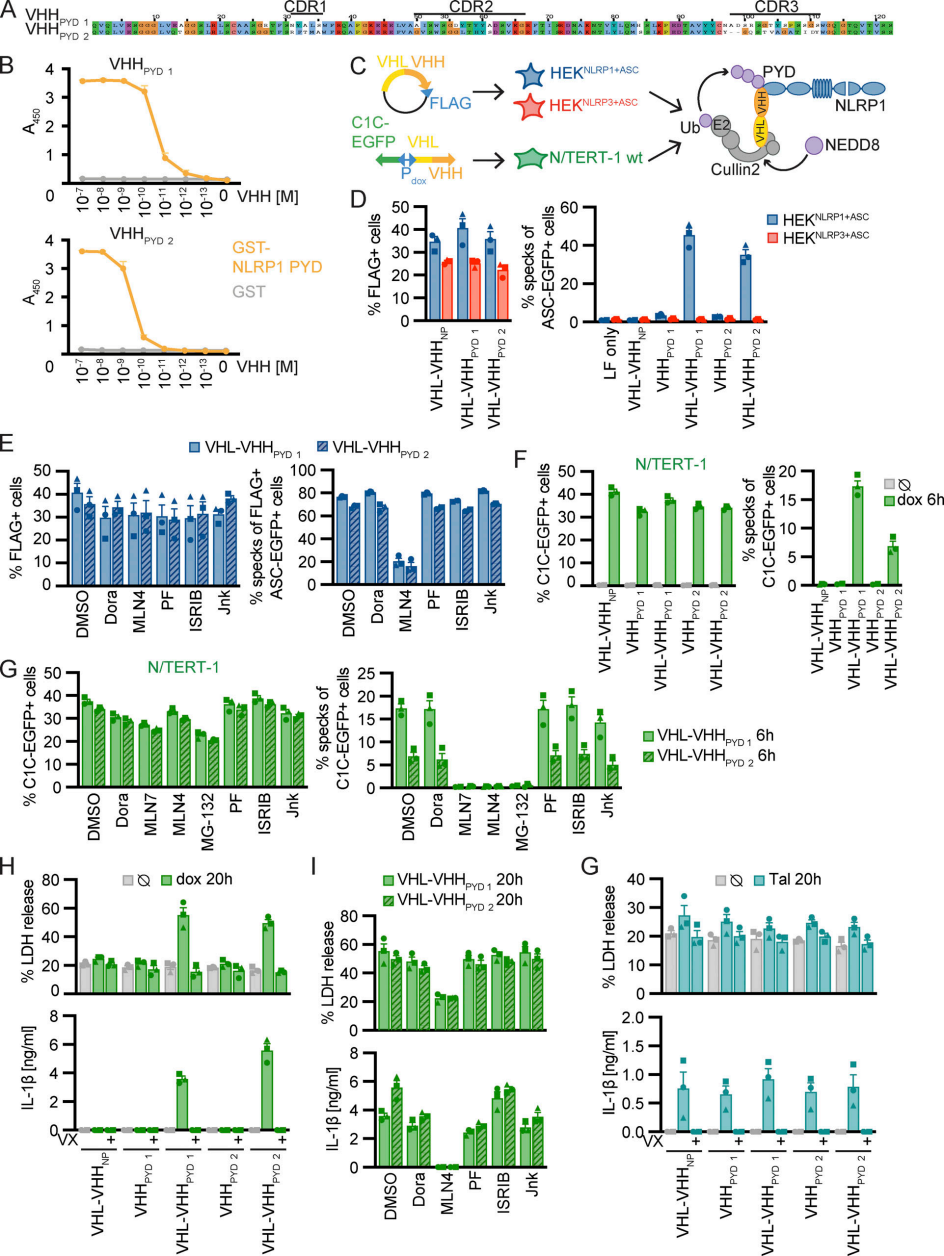
**Activation of NLRP1 by ubiquitination of NLRP1**^**PYD**^ **is independent of p38. (A)** Alignment of NLRP1^PYD^ nanobodies (VHHs) with indicated complementarity determining regions (CDRs). **(B)** Binding of indicated concentration of HA-tagged VHHs to immobilized GST-NLRP1^PYD^ or control protein GST was quantified by ELISA. **(C)** Experimental setup and scheme of VHH-mediated ubiquitination of NLRP1. HEK^NLRP1+ASC^ and HEK^NLRP3+ASC^ cells were transiently transfected with expression vectors for VHL-VHH fusions; N/TERT-1 cells were transduced with lentiviral vectors encoding C1C-EGFP and (VHL-)VHH controlled by a bidirectional dox-inducible promoter. **(D and E)** HEK^NLRP1+ASC^ and HEK^NLRP3+ASC^ cells expressing the indicated FLAG-tagged VHL-VHH fusions or HA-tagged VHHs alone, where indicated in the presence of 10 µM Dora, 1 μM MLN4, 1 μM PF, 200 nM ISRIB, 3 µM Jnk, or DMSO, were analyzed for FLAG expression and ASC-EGFP specks 20 h after transfection as described in Fig. 1. Quantification of specks was limited to FLAG-positive cells in E. **(F–I)** N/TERT-1 cells inducibly expressing C1C-EGFP as well as VHL-VHH or VHH alone were treated with 1 µg/ml dox for the indicated time, where indicated in the presence of 10 µM Dora, 1 μM MLN7, 1 μM MLN4, 1 µM MG-132, 1 μM PF, 200 nM ISRIB, 3 µM Jnk, or DMSO. Expression of C1C-EGFP and (VHL-)VHH was induced in presence of 100 µM VX for analysis of speck assembly in C1C-EGFP–positive cells by flow cytometry (F and G), or in the absence or presence of VX as indicated for analysis of cell death by LDH release or IL-β secretion by HTRF (H and I). **(G)** The indicated N/TERT-1 keratinocytes cell lines described above were stimulated with 30 µM Tal in the absence of dox and analyzed for LDH release and IL-1β secretion as above. Data represents average values (with individual data points) from three independent experiments ± SEM.

We found that NLRP1 inflammasome activation by both the ribotoxic stress response and alphavirus infection requires p38 activation. Both responses also require functional proteasomal degradation, likely degrading the N-terminus of NLRP1 and releasing the active NLRP1^UPA-CARD^. To find out whether degradation of NLRP1 itself relies on p38 activity, we sought to establish an experimental setup to specifically ubiquitinate human NLRP1 at will, similar to the activation of mNlrp1b by a ubiquitin ligase of the human pathogen *Shigella flexneri* (Sandstrom et al., 2019). We immunized alpacas with the PYD of NLRP1 and employed phage display to identify NLRP1^PYD^-specific variable domains of heavy chain-only antibodies (VHH), also described as nanobodies (Fig. 7 A). Specific binding of VHH_NLRP1 PYD 1_ (VHH_PYD__1_) and VHH_NLRP1 PYD__2_ (VHH_PYD__2_) to NLRP1^PYD^ was confirmed by ELISA (Fig. 7 B). Unlike antibodies, nanobodies often function in the reducing environment of the cytosol. They can thus be genetically fused to enzymes to recruit their activity to the nanobody target protein. To explore whether NLRP1^PYD^-specific nanobodies allow activation of NLRP1 by targeted ubiquitination, we next generated expression vectors for fusions of nanobodies to the human ubiquitin ligase receptor von Hippel Lindau (VHL; Fig. 7 C). Endogenous VHL serves as a ubiquitin ligase receptor for modular cullin-2 ubiquitin ligases and mediates the constitutive ubiquitination and proteasomal degradation of HIF1α under normoxic conditions (Haase, 2009), suggesting that overexpression as such will not alter cellular states at normal oxygen levels. Overexpressed VHL-VHH fusions have been successfully used to mediate proteasomal degradation of VHH targets (Fulcher et al., 2017).

Transient overexpression of FLAG-tagged VHL-VHH_PYD__1_ and VHL-VHH_PYD__2_ induced ASC speck formation in >40% of HEK^NLRP1+ASC^ cells (Fig. 7 D). When we gated for FLAG-positive cells, almost 80% of the cells exhibited ASC specks (Fig. 7 E), indicating near complete activation of NLRP1 in cells expressing the fusion protein, likely independent of the bottlenecks of endogenous NLRP1 activation yielding lower response rates. Importantly, NLRP1 was not activated by overexpression of the nanobodies alone, or by VHL fusions to a control nanobody targeting influenza A virus NP (Ashour et al., 2015). Likewise, NLRP3 was not activated by any of the constructs (Fig. 7 D). As expected, inhibition of neddylation blocked NLRP1 inflammasome activation by VHL-VHH_PYD_ in line with the critical role of NEDD8 for the activity of cullin ubiquitin ligases (Fig. 7 E). Importantly, inhibition of p38 activity did not affect NLRP1 activation, demonstrating that p38 activity was not required to execute N-terminal degradation and NLRP1^UPA-CARD^-nucleation of inflammasomes.

While the artificial destabilization of NLRP1 by inducible degradation had been realized in HEK 293T cells ectopically expressing dTAG-NLRP1 (Hollingsworth et al., 2021a), or AID-NLRP1 and TIR1 (Sandstrom et al., 2019), none of the systems allowed controlled ubiquitination of endogenous NLRP1, and thus analysis of the complete downstream response of NLRP1 activation. We thus generated N/TERT-1 keratinocyte cell lines expressing C1C-EGFP as well as VHHs or VHL-VHH fusions under the control of a bidirectional doxycycline (dox)-inducible promoter. Induced expression of VHL-VHH_PYD__1_ and VHL-VHH_PYD__2_ triggered inflammasome assembly detectable as early as 6 h after induction, with faster activation by VHH_PYD__1_ than by VHH_PYD__2_ (Fig. 7 F). Inflammasome responses were substantially higher than after Tal treatment. Neither nanobody expression alone, nor VHL fusions to the control nanobody VHH_NP-1_ induced inflammasome assembly. The rapid inflammasome assembly allowed us to quantify responses in the presence of inhibitors of E1 enzyme (MLN7), the proteasome (MG-132), as well as neddylation (MLN4; Fig. 7 G). We found that inflammasome assembly was completely blocked by either inhibitor, indicating that VHL fusions of NLRP1^PYD^ nanobodies indeed rely on the activation of ubiquitin, cullin ubiquitin ligases, as well the proteasome to mediate ubiquitination and N-terminal degradation of NLRP1. Again, inhibition of p38 did not affect VHL-VHH_PYD_–induced NLRP1 activation. Assembly of ASC specks was accompanied by caspase-1–dependent pyroptotic cell death and robust IL-1β secretion, which was likewise inhibited by neddylation inhibitors, but not p38 inhibitor Dora (Fig. 7, H and I). All generated cell lines exhibited comparable levels of pyroptosis and IL-1β release when treated with Tal in the absence of transgene expression (Fig. 7 G).

We thus demonstrate that ubiquitination of human NLRP1 is sufficient for its activation by N-terminal degradation. Since this well-controlled system allows the precise direct activation of NLRP1 in the absence of any upstream signals, we could prove that p38 activation is involved in a signaling step upstream of NLRP1 ubiquitination and is not required for activation by targeted ubiquitination.

### Strong p38 activity is sufficient for NLRP1 activation

**Figure 8.**
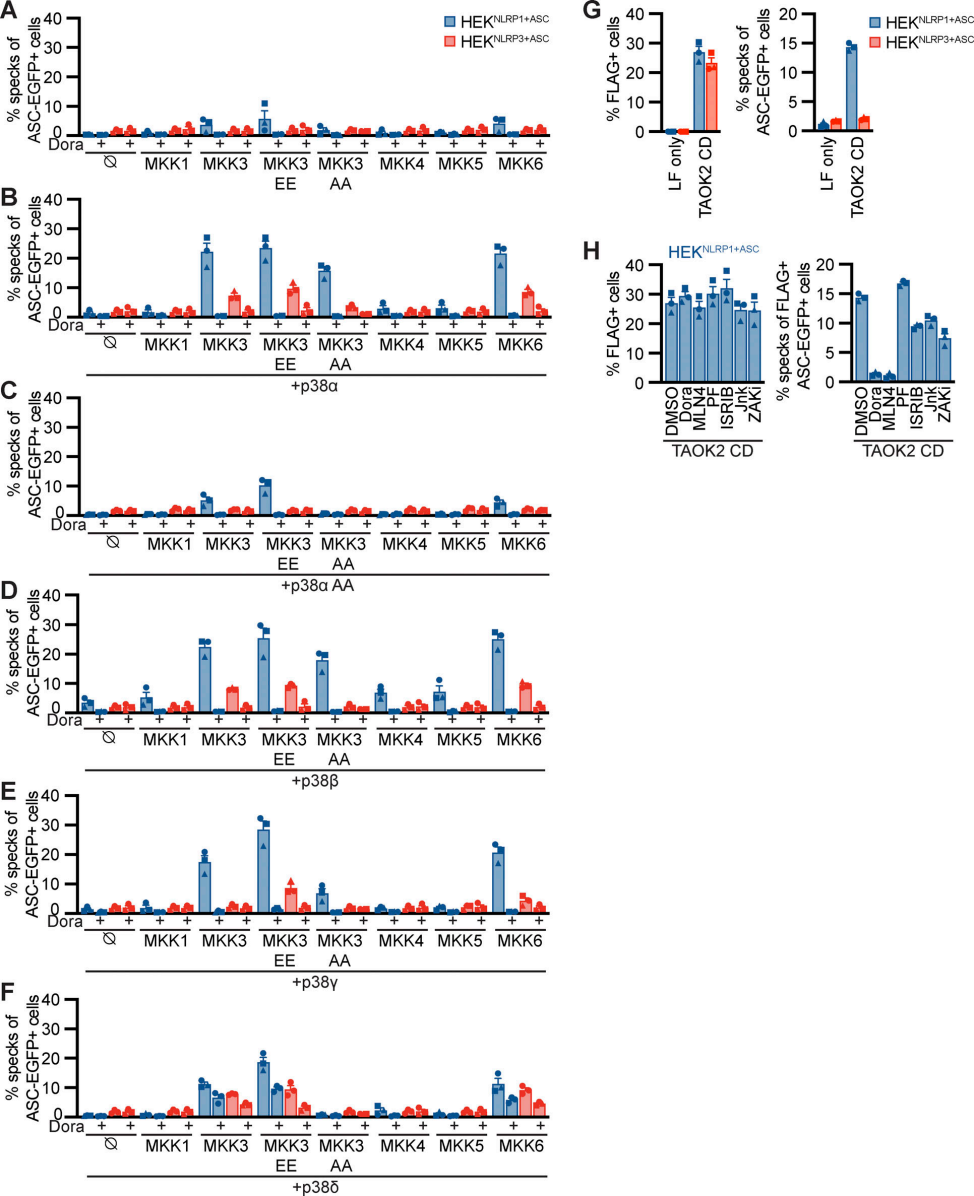
**P38 activation is sufficient for NLRP1 activation. (A–F)** HEK^NLRP1+ASC^ or HEK^NLRP3+ASC^ cells were transiently transfected with empty vector (A), expression vectors for p38α (B), p38α T180A Y182A (p38α AA; C), p38β (D), p38γ (E), or p38δ (F) in combination with empty vector (⦰) or the indicated expression vectors for MKK1, MKK3, MKK3 S218E T222E (MKK3 EE), MKK3 S218A T222A (MKK3 AA), MKK4, MKK5, or MKK6 for 20 h. Where indicated, cells were treated with 10 µM Dora. Cells with ASC-EGFP specks were quantified by flow cytometry as described in Fig. 1. **(G and H)** HEK^NLRP1+ASC^ or HEK^NLRP3+ASC^ cells were transiently transfected with expression vectors for FLAG-tagged catalytic domain of TAOK2 (TAOK2 CD), where indicated in the presence of 10 µM Dora, 1 μM MLN4, 1 μM PF, 200 nM ISRIB, 3 µM Jnk, 100 nM ZAKi, or DMSO, and analyzed for FLAG expression and ASC-EGFP specks in FLAG-positive cells 20 h after transfection. Specks of total ASC-EGFP–positive cells were quantified in case of LF only controls. Data represents average values (with individual data points) from three independent experiments ± SEM.

Having shown that p38 activity was required for NLRP1 activation by the ribotoxic stress response and alphavirus infection, we next assessed whether p38 activation itself was sufficient for NLRP1 activation. We transiently overexpressed the MAP2K MKK1, MKK3, MKK4, MKK5, and MKK6 in HEK^NLRP1+ASC^ and HEK^NLRP3+ASC^ cells. We also included phosphomimetic glutamate or inhibitory alanine mutants of S218 and T222 in the activation loop of MKK3, yielding MKK3 EE or MKK3 AA, respectively. Overexpression of MKK3, MKK3 EE, or MKK6, the upstream kinases that directly phosphorylate p38, indeed initiated weak NLRP1 inflammasome activation (Fig. 8 A). Activation was sensitive to the pan-p38 inhibitor Dora, confirming that the observed inflammasome assembly indeed relied on p38 activity. Importantly, the related MAP2K MKK1, MKK4, and MKK5, which phosphorylate other MAPK but not p38, did not initiate inflammasome assembly in HEK^NLRP1+ASC^. Overexpression of none of the MAP2K activated NLRP3 inflammasome assembly in HEK^NLRP3+ASC^. Experiments to artificially trigger p38 activity typically rely on the combined ectopic expression of p38 isoforms and their upstream kinases (Raingeaud et al., 1996). In line with these findings, overexpression of p38α, p38β, p38γ, or p38δ (Fig. 8, B and D–F) alone did not initiate NLRP1 inflammasome assembly. However, combined expression of p38 isoforms with MKK3, MKK3 EE, or MKK6 triggered robust NLRP1 inflammasome assembly. Interestingly, MKK3 AA bearing a mutated activation loop that cannot be phosphorylated still enhanced p38α and p38β inflammasome assembly. None of the other MAP2K stimulated p38-mediated NLRP1 activation. Dora treatment abrogated all observed NLRP1 inflammasome assembly, indicating that the observed ASC specks genuinely relied on p38 activity. In line with the higher IC_50_ values of this drug for p38δ (Kuma et al., 2005), inhibition was incomplete for this isoform. Co-expression of MAP2K with p38α bearing a mutated activation loop (p38α T180A Y182A) did not enhance NLRP1 activation beyond the levels observed upon overexpression of upstream kinases alone (Fig. 8 C). Remarkably, p38 activation by overexpression also induced some level of NLRP3 activation, albeit to substantially lower levels than observed for NLRP1.

NLRP1 activation by SFV infection involved ZAKα and p38, but perhaps other MAP3K as well, as residual inflammasome assembly was also observed in the absence of active ZAKα. We therefore tested whether alternative MAP3K than ZAKα can stimulate the NLRP1 inflammasome in HEK reporter cells. We transiently expressed the catalytic domain of TAOK2 (aa 1–349) in HEK^NLRP1+ASC^ and HEK^NLRP3+ASC^ and detected a robust inflammasome response in the NLRP1, but not NLRP3, reporter cells (Fig. 8 G). This response was completely blocked by inhibitors of p38 and neddylation, but not inhibitors of ZAKα, proving that p38 activation by different MAP3K can activate NLRP1 in the HEK cell model (Fig. 8 H).

### p38 directly phosphorylates NLRP1 in the N-terminal linker region

**Figure S5.**
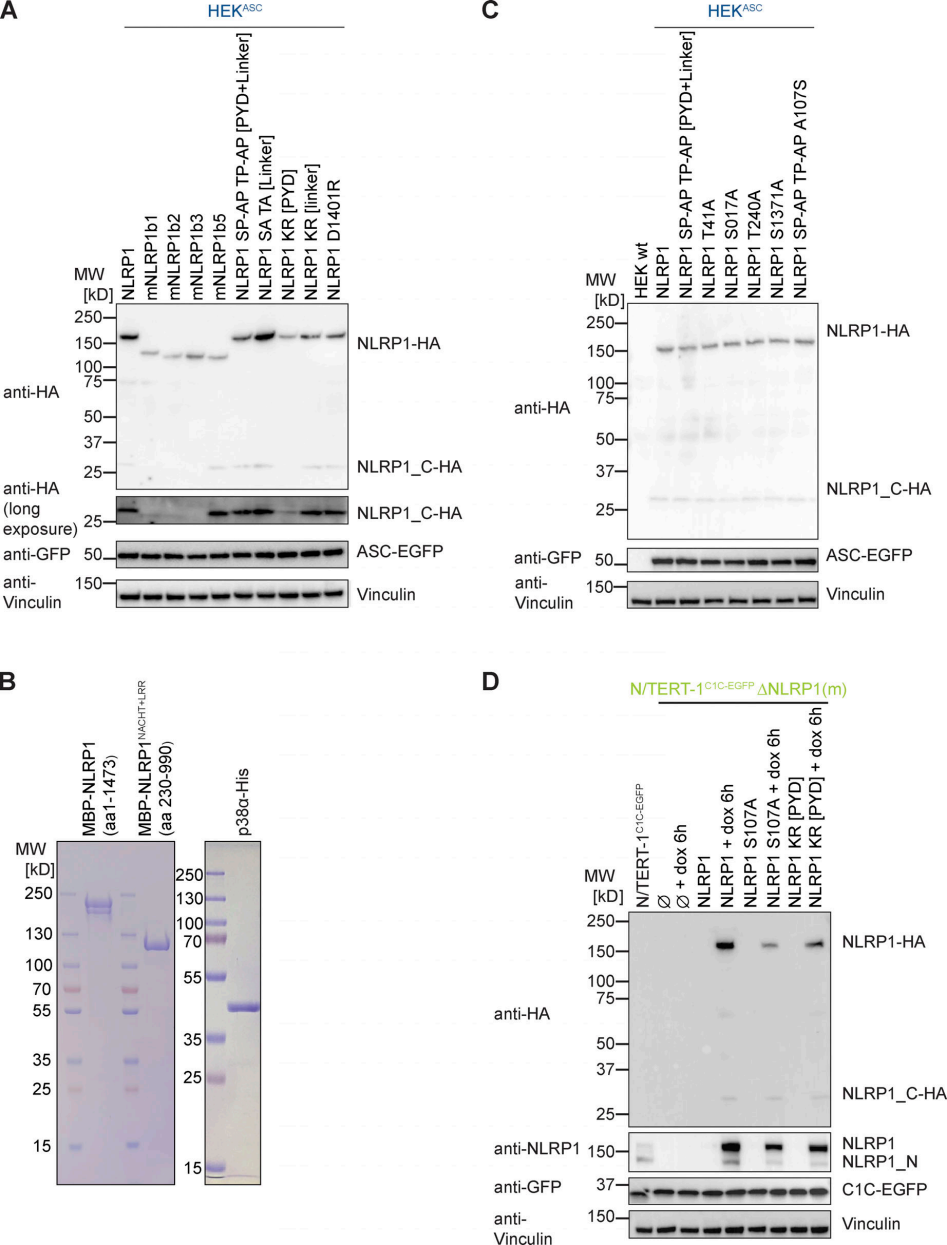
**Supplementary immunoblots and SDS-PAGE gels for Figs. 9 and 10. (A and C)** Lysates of HEK 293T (HEK) or HEK^ASC^-based cell lines constitutively expressing HA-tagged human NLRP1, the indicated murine Nlrp1b alleles (A), or the indicated human NLRP1 mutants (A and C) were analyzed by immunoblot with the indicated antibodies to verify expression of NLRP1-HA variants and ASC-EGFP. **(B)** Purified MBP-NLRP1 and MBP-NLRP1^NACHT+LRR^ (left), as well as purified, activated p38α-His (right) used in the in vitro kinase assay in Fig. 9 B were analyzed by SDS-PAGE and Coomassie staining. **(D)** Lysates of N/TERT-1^C1C-EGFP^, monoclonal N/TERT-1^C1C-EGFP^ ΔNLRP1, or N/TERT-1^C1C-EGFP^ ΔNLRP1 reconstituted with the dox-inducible expressed NLRP1 or the indicated NLRP1 mutants were analyzed by immunoblot with the indicated antibodies to verify constitutive expression of C1C-EGFP and dox-induced expression of NLRP1 after 6 h. Data displays experiment representatives of two independent experiments. MW, molecular weight. Source data are available for this figure: SourceData FS5.

**Figure 9.**
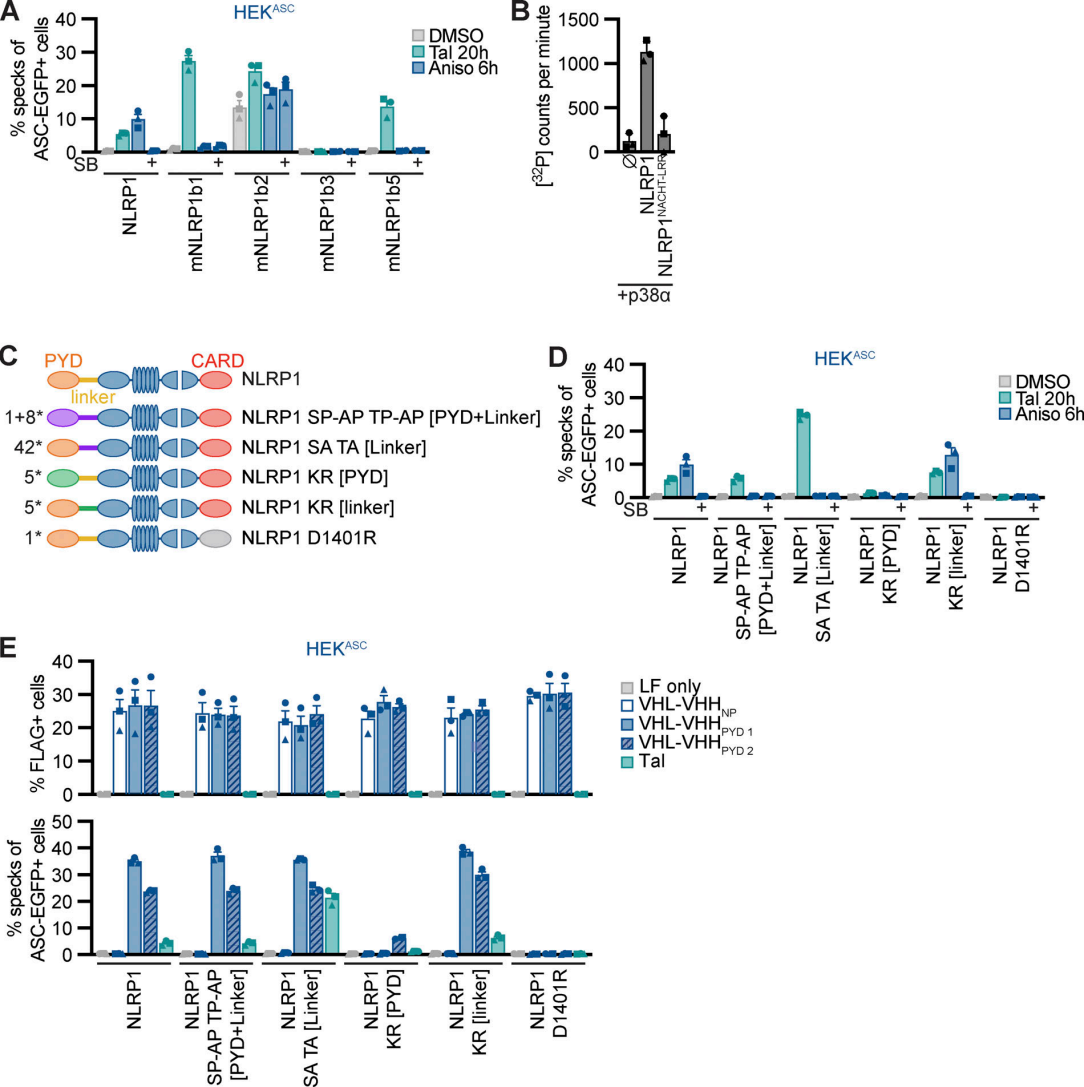
**P38 directly phosphorylates human NLRP1 in the linker region, which is critical for activation by the ribotoxic stress response. (A)** HEK^ASC^-based cell lines constitutively expressing human NLRP1 or the indicated murine Nlrp1b alleles were treated with DMSO, 30 µM Tal, or 15 µM Aniso for the indicated times. Cells with ASC-EGFP specks were quantified by flow cytometry as described in Fig. 1. **(B)** 0.2 µM activated p38α was incubated with 7 µM MBP-NLRP1, MBP-NLRP1^NACHT-LRR^ (aa 230–990), or no substrate (⦰) at 30°C for 30 min in kinase buffer containing [^32^P]-γ-ATP. Protein-associated ^32^P was quantified by liquid scintillation counting. **(C)** Scheme of generated HEK^ASC^-based cell lines constitutively expressing human NLRP1 mutants. The number of mutated amino acids in the PYD or linker is indicated to the left of the molecule. **(D and E)** HEK^ASC^ cell lines expressing the NLRP1 mutants described in C were stimulated and analyzed as in A (D), or transiently transfected with expression vectors for FLAG-tagged (VHL)-VHH for 20 h (E) and analyzed for FLAG expression and ASC-EGFP specks (in FLAG-positive cells, except for LF only controls). Data represents average values (with individual data points) from three independent experiments ± SEM (A, D, E) or SD (B).

Human NLRP1 substantially differs from murine Nlrp1b alleles in the N-terminal PYD and the subsequent linker, which are not found in mNRLP1b (Sastalla et al., 2013). To test if the ribotoxic stress response activates both human and murine NLRP1(b), we generated polyclonal cell lines expressing HA-tagged human NLRP1 or the different mNlrp1b alleles in the background of HEK 293T cells expressing ASC-EGFP (HEK^ASC^), exploiting that all variants can interact with and activate human ASC. Comparable expression of all murine Nlrp1 alleles was verified by immunoblot (Fig. S5 A). All variants except for mNlrp1b3 were activated by Tal treatment as described (Gai et al., 2019; Fig. 9 A). Of note, stable expression of NLRP1b2 led to substantial background activation. Cells expressing human NLRP1 assembled ASC specks in response to Aniso treatment, but none of the murine alleles initiated ASC specks beyond background levels (as defined by the ASC speck response in the presence of p38 inhibitor SB). Thus, murine NLRP1b is not activated by the ribotoxic stress response, indicating that the N-terminal PYD and linker of human NLRP1 may determine responsiveness to this mode of activation.

Human NLRP1 activation by targeted ubiquitination of the PYD did not require p38 activity, and neither did the activation of NLRP1 by Tal. This indicates that p38 is not required for NLRP1 function per se, but that p38 contributes to an upstream signaling cascade that integrates information from different stress signaling cascades to activate NLRP1. We could show that p38 activation was sufficient for NLRP1 inflammasome activation, and that activation was likely not a consequence of p38-dependent gene expression. To test whether p38 can directly phosphorylate NLRP1, we performed radiometric in vitro kinase assays with recombinant activated p38α and purified full-length NLRP1 expressed in insect cells as a fusion to maltose-binding protein (MBP-NLRP1, Fig. S5 B). We found that MBP-NLRP1 could be robustly phosphorylated by activated p38α, as detected by transfer of radioactive phosphate from [^32^P]-γ-ATP to purified NLRP1 protein (Fig. 9 B). Importantly, MBP-NLRP1^aa 230-990^, comprising part of the linker, NACHT, and LRR of NLRP1, could not be phosphorylated by p38α. This indicates that either the N-terminal 230 amino acids or the C-terminal FIIND-CARD of NLRP1 are the substrate of p38. Combined with the results from the mNlrp1b stimulation experiments, it is tempting to speculate that p38 phosphorylates human NLRP1 at the N-terminus, which is why murine NLRP1 is not responsive to Aniso.

To evaluate the role of NLRP1 phosphorylation and ubiquitination for activation, we generated polyclonal cell lines expressing HA-tagged wildtype or mutant NLRP1 in the background of HEK^ASC^ cells. As Aniso-responsive human NLRP1 differs from unresponsive murine NLRP1b primarily in the N-terminal PYD and linker region, we mutated serines, threonines, and lysines in the N-terminus of NLRP1 (Fig. 9 C). Robust expression of NLRP1 variants was verified by immunoblot (Fig. S5 A). Expression of wildtype NLRP1 allowed inflammasome activation by Tal, Aniso, and ectopic expression of VHL-VHH_PYD_ as before (Fig. 9, D and E). NLRP1 containing the point mutation D1401R that prevents oligomerization of the C-terminal CARD (Hollingsworth et al., 2021b) was not activated by any of these stimuli. As p38 preferentially phosphorylates the amino acid motifs SP and TP (Canovas and Nebreda, 2021), we mutated every serine and threonine followed by proline in the PYD (aa 1–92) and linker region (aa 93–327) of NLRP1 to alanine (1 + 8 mutations in PYD and linker, respectively). HEK^ASC^ cells expressing NLRP1 SP-AP TP-AP [PYD + linker] responded normally to Tal and ectopic VHL-VHH_PYD_, but were not activated by Aniso treatment (Fig. 9, D and E). A mutant of NLRP1 with all serines and threonines in the linker mutated to alanine, NLRP1 SA TA [linker] (42 mutations), was strongly activated by Tal, which may be explained by its higher expression (Fig. S5 A). Nevertheless, cells expressing NLRP1 SA TA [linker] were not activated by Aniso either. This suggests that phosphorylation of canonical p38 substrate motifs in the NLRP1 linker is critical for p38-mediated activation of NLRP1, but not for p38-independent activation. Our results indicate that all triggers of NLRP1 inflammasome activation rely on proteasome activity, neddylation, and the E1 enzyme. We thus generated HEK^ASC^ cell lines expressing NLRP1 with all five lysines in the PYD, or all five lysines in the linker region mutated to arginine to prevent ubiquitination. We found that mutation of lysines in the PYD completely abrogated responses to Tal, Aniso and VHL-VHH_PYD__1_ transfection (Fig. 9, D and E). Of note, NLRP1 KR [PYD] was expressed somewhat weaker than the other mutants (Fig. S5 A). Yet the strong VHL-VHH_PYD__1_–induced response was completely abolished in these cells, which is unlikely based on weak expression alone. Interestingly, transient expression of VHL-VHH_PYD__2_ in NLRP1 KR [PYD] cells still induced a weak inflammasome response. It is possible that binding of VHL-VHH_PYD__2_ enables ubiquitination of lysines outside the PYD, i.e., in the linker region. In contrast, mutation of lysines in the linker did not affect responses at all. This suggests that ubiquitination of lysines in the PYD of NLRP1 is crucial for activation by p38-dependent and p38-independent stimuli.

We next set out to identify phosphorylation sites on NLRP1 during activation. HEK^NLRP1+ASC^ cells were treated with Aniso for 1 h or left untreated, followed by immunoprecipitation of NLRP1-HA with anti-HA antibodies. Proteins on magnetic anti-HA beads were directly subjected to digestion with proteases to generate peptides for analysis by MS. As the resulting peptides after trypsin cleavage did not represent the linker region of NLRP1 well, we also used chymotrypsin as an alternative protease. We identified at least two amino acids in the linker region that were phosphorylated after Aniso treatment: serine 107 in chymotryptic peptides as well as threonine 240 in tryptic peptides. Notably, both phosphorylation sites were followed by prolines, suggesting they were canonical p38 substrates. As the coverage of the linker was incomplete, it is possible that our efforts did not identify all phosphorylation sites in the linker region. We further identified Aniso-induced phosphorylation sites in the C-terminal regions of NLRP1, including serines 1,017, 1,022 and 1,371 as well as threonine 993 in tryptic peptides. Serine 1,371 and threonine 993 were followed by prolines and thus were part of canonical p38 motifs.

**Figure 10.**
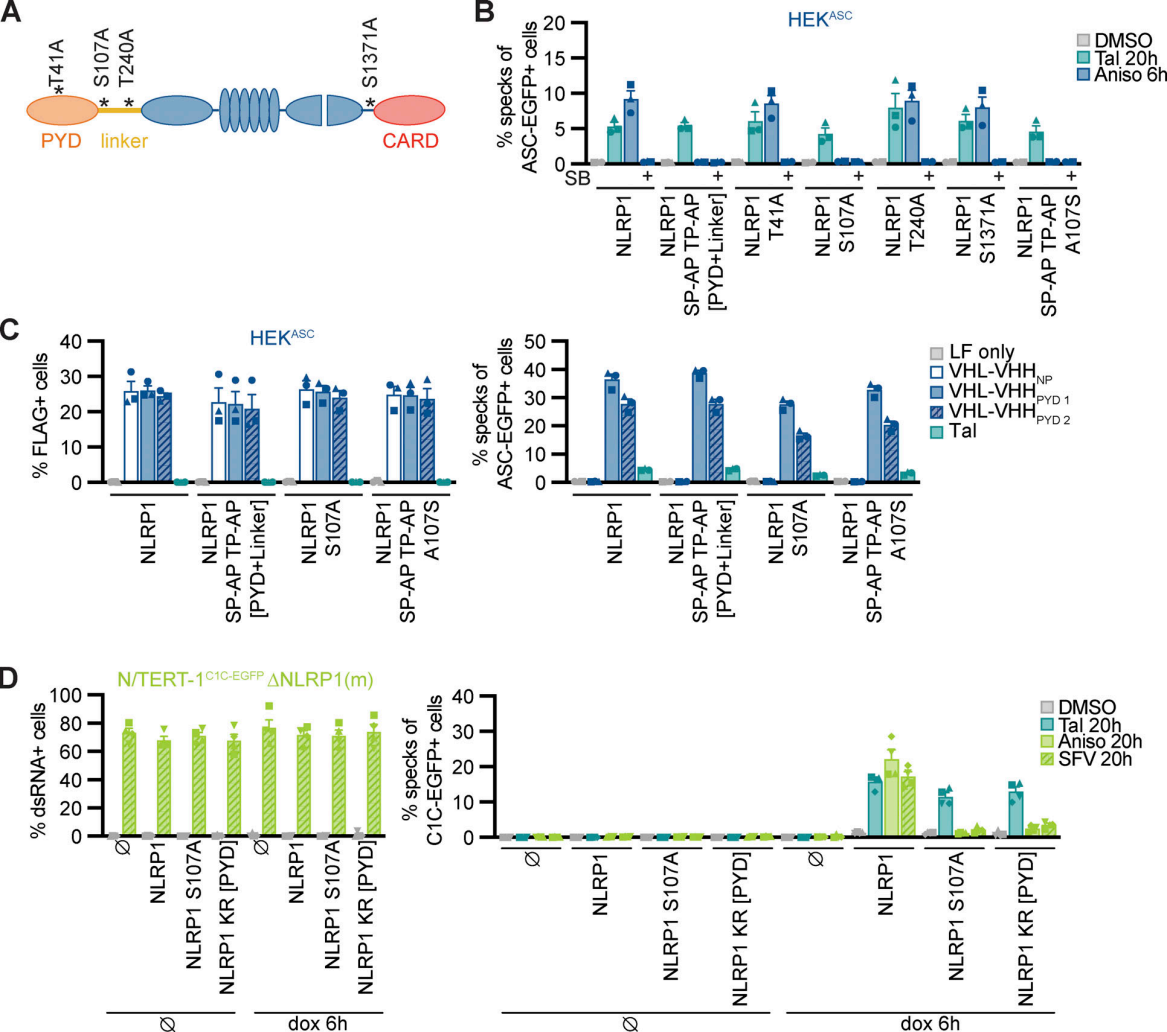
**Serine 107 in the linker region is critical for p38-dependent NLRP1 activation. (A)** Overview of mutants of NLRP1 phosphorylation sites. Phosphosite T41 was described previously, while all other phosphosites were detected in immunoprecipitated NLRP1-HA after stimulation with 15 μM Aniso for 1 h. **(B and C)** HEK^ASC^-based cell lines constitutively expressing human NLRP1 or the indicated mutants were treated with DMSO, 30 µM Tal, or 15 µM Aniso for the indicated times and analyzed for specks as described in Fig. 1 (B), or transiently transfected with expression vectors for FLAG-tagged VHL-VHH for 20 h (C) and analyzed for FLAG expression and ASC-EGFP specks (in FLAG-positive cells, except for LF only controls). **(D)** Monoclonal NLRP1 knockout N/TERT-1^C1C-EGFP^ cells reconstituted with dox-inducible NLRP1 or the indicated NLRP1 mutants were treated with dox for 6h. Cells were subsequently stimulated with DMSO, 30 µM Tal or 15 µM Aniso, or infected with SFV at an MOI of 5 for 20 h in the presence of 100 μM VX. Infected cells were stained with antibodies for dsRNA and infection and speck assembly (in infected cells in case of SFV) were quantified by flow cytometry. Data represents average values (with individual data points) from three or four independent experiments ± SEM.

To analyze the functional role of identified p38 phosphorylation sites in the linker region of NLRP1, we next generated cell lines expressing HA-tagged alanine mutants NLRP1 S107A and T240A. As controls, we also included NLRP1 mutants of the previously identified canonical p38 phosphorylation site T41 in the PYD (Casado et al., 2013), or S1371 in the UPA domain (Fig. 10 A). All NLRP1 mutants were expressed to equal levels (Fig. S5 C). All alanine mutants responded normally to Tal and Aniso, except NLRP1 S107A. The latter mutant was unresponsive to Aniso stimulation, and thus resembled the NLRP1 SP-AP TP-AP [PYD + linker] mutant (Fig. 10 B). The S107A mutation did not influence the NLRP1 response to Tal and VHL-VHH_PYD_ expression, confirming the functionality of this mutant in p38-independent activation (Fig. 10, B and C). Thus, phosphorylation of the SP motif at S107 is critical for Aniso-induced NLRP1 activation. Next, we tested the rescue mutant NLRP1 SP-AP TP-AP [PYD + linker] A107S, in which only the SP motif containing S107 was restored to wildtype, while all other p38 motifs in PYD and linker remained mutated. This NLRP1 variant was still not activated by Aniso (Fig. 10 B), suggesting that S107 is not the only important serine/threonine. Other serine or threonine residues may either be substrates of p38, or may be required for other functions of NLRP1, e.g., binding to p38 or ubiquitin ligases.

Due to the lower response rates in the polyclonal NLRP1 HEK cells, we could not determine the role of the SP motif at S107 in SFV-induced NLRP1 activation with this setup. Thus, we reconstituted NLRP1 knockout N/TERT-1^C1C-EGFP^ cells with dox-inducible HA-tagged NLRP1, NLRP1 S107A and NLRP1 KR [PYD]. We selected polyclonal cell lines and confirmed robust inducible expression of the NLRP1 mutants (Fig. S5 D). We treated these cells with Tal, Aniso, or SFV, in absence or presence of dox. N/TERT-1 cells reconstituted with wildtype NLRP1 responded to all stimuli, and all mutants responded to Tal treatment with similar levels of inflammasome assembly, despite the somewhat varying expression levels of NLRP1 (Fig. 10 D). Cells expressing NLRP1 S107A did not respond to Aniso and SFV, confirming that phosphorylation of the SP motif at S107 is critical for the p38-dependent activation of NLRP1 by the ribotoxic stress response and SFV. In line with the universal role of proteasome-dependent N-terminal degradation of NLRP1, cells reconstituted with the lysine mutant NLRP1 KR [PYD] were unresponsive to Aniso and SFV. Interestingly, NLRP1 lacking lysine residues in the PYD was still activated by Tal in N/TERT keratinocytes, which was not the case in the HEK NLRP1 reporter cells.

In sum, we find that p38 activation in reconstituted HEK^NLRP1+ASC^ cells is sufficient to initiate the assembly of NLRP1 inflammasomes and that p38 can directly phosphorylate NLRP1 in vitro. Serine and threonine residues in canonical p38 SP and TP motifs in the linker region of NLRP1 are required for Aniso-mediated NLRP1 activation. We identified serine 107 as critical for p38-mediated activation of NRLP1, indicating that this residue is phosphorylated by p38. Ubiquitination of the NLRP1^PYD^ is required for activation of NLRP1 by both Aniso and SFV, and was further found to be sufficient to initiate NLRP1 inflammasome assembly. It is well possible that phosphorylation of the linker of NLRP1 marks NLRP1 for ubiquitination by yet to be identified E3 ligases.

## Discussion

As the largest organ, the human skin is exposed to a variety of physico-chemical and biological insults, including UV irradiation and infection with arthropod-borne viruses. These can trigger the assembly of NLRP1 inflammasomes in keratinocytes (Fenini et al., 2020; Bauernfried et al., 2021). By using reporters for ASC speck assembly rather than cytokine measurements alone, we could robustly quantify inflammasome assembly in keratinocytes, even though the fraction for responding cells is lower than observed for myeloid cells. The lower response rate of keratinocytes may avoid massive pyroptotic cell death of this abundant cell type in response to threats that can be locally controlled.

In this study, we find that a number of different stress signals, including UVB irradiation, inhibition of translation by antibiotics, as well as infection with arthropod-borne alphaviruses converge on the activation of p38 MAP kinase signaling. P38 kinase activity lastly activates NLRP1 inflammasomes by direct phosphorylation. Importantly, we find that strong p38 activation is sufficient to activate NLRP1, suggesting that p38 signaling acts as a rheostat integrating different stress responses to ultimately trigger irreversible NLRP1 inflammasome assembly only if a critical threshold is overcome.

Earlier work by Fenini and colleagues (Fenini et al., 2018a) established that keratinocytes can assemble NLRP1 inflammasomes in response to UV. Yet, neither the molecular mechanism, nor the relationship to other NLRP1 activators was known. Our results link inflammation in keratinocytes to the UV-induced ribotoxic stress response (Wang et al., 2005; Iordanov et al., 1998) and the underlying MAPK signaling cascade (Vind et al., 2020a, 2020b). We conclude the following molecular model: UV-induced damage of ribosomal RNA or mRNA activates the ribotoxic stress response in keratinocytes. The same pathway is initiated by interfering with ribosome function, such as inhibition of peptide bond formation or peptide translocation by Aniso or Lacti, respectively. Other toxins that activate the ribotoxic stress response, such as ricin and diphtheria toxin, likely stimulate the same pathway. Ribotoxic stress responses trigger a phosphorylation cascade entailing the activation of ZAKα (MAP3K), MKK3/MKK6 (MAP2K), and finally p38 (MAPK). We find that p38α plays a predominant role in the activation of NLRP1 inflammasomes in keratinocytes. Yet, ectopic activation of any p38 isoform by co-expression of its upstream kinases MKK3 or MKK6 is sufficient to initiate NLRP1 inflammasome assembly. Further, other MAP3K than ZAKα, as shown for ectopically expressed TAOK2 catalytic domain, can initiate p38 and NLRP1 activation in the absence of ZAKα activity. P38 can directly phosphorylate the N-terminal linker region of NLRP1, with one critical SP motif at S107, and thereby triggers NLRP1 activation in a pathway that relies on neddylation, ubiquitin activation, and proteasome activity. Murine Nlrp1b alleles are not responsive to Aniso, which is in line with the finding that activation relies on critical serine 107 found in the linker region of human NLRP1, but not murine NLRP1b. Based on our data, we propose that phosphorylation of at least one canonical p38 motif in the NLRP1 linker allows the ubiquitination of the NLRP1 PYD by NEDD8-dependent cullin ligases, followed by N-terminal degradation, recruitment of ASC by NLRP1^UPA-CARD^, as well as assembly of ASC specks. ASC specks, in turn, are the sites of caspase-1 activation, resulting in IL-1β maturation and gasdermin D–dependent cell death by pyroptosis. Of note, p38 signaling cascades had previously been proposed to regulate inflammasomes in keratinocytes, although neither the mode of p38 activation, nor the substrates of p38 had been addressed or identified (Fenini et al., 2018b). In the course of the revision of this manuscript, a concurrent study was published that independently identified the ZAKα-mediated ribotoxic stress response as a mechanism of NLRP1 activation (Robinson et al., 2022).

Alphaviruses are transmitted by mosquito bites and likely first replicate in keratinocytes and/or fibroblasts in the skin before the infection is spread by migratory dendritic cells. A sensitive response system in the skin is therefore beneficial to detect and contain infecting alphaviruses before any systemic spread can occur. Even though full CHIKV replication was reported to be impaired in keratinocytes (Bernard et al., 2015), infection of this non-permissive cell type seems to be sufficient to initiate inflammatory responses. It is likely that alphaviruses, in turn, have evolved mechanisms to escape detection by NLRP1. Remarkably, we find that NLRP1 is exclusively activated by those alphaviruses that mediate strong p38 activation. The viral molecules exposed in the cytosol of cells infected with different alphaviruses are expected to be similar. The most parsimonious explanation for the differential activation of NLRP1 by different alphaviruses therefore seems to be that some alphaviruses can control p38 activation in the host and thus prevent NLRP1 activation.

Remarkably, viral NLRP1 activation does not seem to exclusively rely on ZAKα, suggesting that different MAP3K can feed into the same activation mechanism, highlighting the role of p38 as a central signaling hub that integrates information from various inputs. Cytosolic delivery of dsRNA is sufficient to activate NLRP1 in a p38-dependent manner in keratinocytes, but not in reporter HEK cells. This suggests that dsRNA contributes to p38 stimulation, but that other cues may be required to activate p38 and NLRP1. Such additional signals may be provided by infection, as SFV infection was able to activate NLRP1 in both keratinocytes and HEK cells with reconstituted NLRP inflammasomes. Future studies will address how alphavirus infection activates ZAKα and other MAP3K. Of note, alphaviruses rather shut-off host cell gene expression by interfering with transcription rather than translation (Fros and Pijlman, 2016). Interestingly, distinct mRNA secondary structures causing ribosome stalling allow alphaviruses to initiate mRNA translation despite eIF2 phosphorylation, which normally prevents translation in conditions of protein kinase R activation or other types of stress (Toribio et al., 2016).

As a first line of defense, the skin encounters a variety of biotic and abiotic stresses that have to be interpreted to elicit inflammation with highest sensitivity, while at the same time limiting unnecessary collateral damage. We find that p38 MAPK signaling integrates different stress inputs and can—as a result of sufficient activation—directly phosphorylate NLRP1 to trigger its activation. Phosphorylation of the NLRP1 linker is thus a novel mechanism to destabilize the NLRP1 N-terminus. In contrast, activation of NLRP1 by direct ubiquitination, cleavage of the N-terminus by viral proteases, or inhibition of DPP9 does not require p38. The contribution of p38-mediated NLRP1 activation to human pathology is difficult to assess in the absence of established in vivo models of human NLRP1. Many stress-related triggers of NLRP1 may also cause other forms of programmed cell death or necrosis, while inflammasome-mediated release of the potent pro-inflammatory cytokine IL-1β will uniquely alter the response in the tissue. While cytokine levels and immunoblot bands are highly dependent on the experimental setup, the fraction of cells that assemble ASC specks is an objective measure for inflammasome responses at endogenous NLRP1 levels. With up to 2% (UV), 50% (Aniso), and 10% (SFV) of all affected keratinocytes responding, the described pathways have the potential to contribute to strong inflammation in tissues that are mostly composed of abundant keratinocytes.

While p38 is ubiquitously expressed throughout the body, NLRP1 expression is limited to a few distinct cell types. In keratinocytes, p38 signaling can thus be co-opted to feed into a more drastic (and widespread) inflammatory response by activating NLRP1. Our work thus reveals a common mechanism for diverse triggers of NLRP1 inflammasome activation and provides a detailed molecular mechanism.

## Materials and methods

### Cell lines

HEK 293T cells (Cat# CRL-3216, RRID: CVCL_0063; ATCC), and Syrian baby hamster kidney (BHK)-21 cell clone BSR-T7/5 (*Mesocricetus auratus*, a kind gift of Sean Whelan, Harvard Medical School, Boston, MA) were cultivated in DMEM containing 10% FBS and GlutaMax; BHK-21/J cells (*Mesocricetus auratus*, a kind gift of Charles M. Rice, Rockefeller University, New York, NY) were cultivated in MEM containing 7.5% FBS, 1% nonessential amino acids, and 1% L-glutamine (all Thermo Fisher Scientific). Human N/TERT-1 keratinocytes (a kind gift of James Rheinwald, Harvard Medical School, Boston, MA) were cultivated in keratinocyte serum-free medium (Thermo Fisher Scientific) supplement with 0.5× bovine pituitary extract, 0.2 mg/ml EGF, 1× penicillin/streptomycin, and 0.3 mM CaCl_2_ (resulting in a final concentration of 0.4 mM CaCl_2_). Primary NHEK from a single donor were obtained from PromoCell and cultivated in Keratinocyte Growth Medium 2 (PromoCell). Unless otherwise mentioned, genetically modified cell lines were generated by lentiviral transduction using lentivirus produced with packaging vectors psPax2 and pMD2.G (kind gifts from Didier Trono, École polytechnique fédérale de Lausanne, Lausanne, Switzerland) and antibiotic selection. Lentiviral vectors for constitutive expression under the control of the pUbC were constructed by Gateway cloning (Thermo Fisher Scientific) using vectors modified from pRRL (a kind gift of Susan Lindquist, Whitehead Institute of Biomedical Research, Cambridge, MA). HEK 293T cells expressing ASC-EGFP under control of pUbC, HEK^ASC^, were generated by transduction with a dilution series of lentivirus to achieve single insertions. Individual clones were cultivated and tested for minimal background of ASC-EGFP specks. Derivatives of HEK^ASC^ expressing NLRP1-HA (HEK^NLRP1+ASC^; cell line H8-1), or NLRP3-HA (HEK^NLRP3+ASC^; cell line H98) under the control of pUbC from single insertions were generated using the same protocol and clones selected based on optimal signal-to-noise ratio and background. Polyclonal HEK^ASC^ cell lines expressing wildtype and mutant human NLRP1 as well as murine Nlrp1b alleles under the control of pUbC were generated by lentiviral transduction with virus multiplicities of infection (MOIs) that permit multiple insertions. N/TERT-1 expressing caspase-1^CARD^-EGFP (C1C-EGFP) controlled by pUbC (N/TERT-1^C1C-EGFP^, cell line K14), were similarly generated by lentiviral transduction. Likewise, NHEK^C1C-EGFP^ cells were generated by transduction of NHEK with the same C1C-EGFP lentiviral constructs but without subsequent antibiotic selection. Knockouts of N/TERT-1^C1C-EGFP^ were generated by lentiviral transductions using vectors modified based on pLenti CRISPR v2 (a kind gift of Feng Zhang, Broad Institute, Cambridge, MA; see Table S1 for sgRNA target sequences). Monoclonal knockouts were generated using limiting dilutions of polyclonal cell lines generated by lentiviral transduction, and selection with antibiotics; best clones were selected based on analysis with OutKnocker (Schmid-Burgk et al., 2014) and immunoblot. Polyclonal knockouts were generated by lentiviral transduction and antibiotic selection and verified by immunoblot. Reconstitution of NLRP1 expression in NLRP1 knockout N/TERT-1 keratinocytes was achieved by transduction with lentiviral vectors based on pInducer20 (Meerbrey et al., 2011) and antibiotic selection. Derivatives of N/TERT-1 expressing C1C-EGFP and VHHs or VHL-VHH fusions under a bidirectional dox-inducible promoter were generated using lentiviral vector pInducer20bi-NA, a derivative of pInducer20-NA (Schmidt et al., 2016a), using the promoter from pTRE3G-BI (Takara Bio).

### Viruses

All experiments involving viruses were conducted in respective Biosafety Level 2 or 3 laboratories. SFV 4 (a kind gift of Giuseppe Balistreri and Ari Helenius, ETH Zurich, Zurich, Switzerland), SINV strain Toto 1,101 (recovered from in vitro transcribed RNA [Rice et al., 1987]), and VSV strain Indiana (recovered from plasmid as described in Whelan et al. [1995]) were amplified in BSR-T7 cells. EEEV, VEEV, MAYV, ONNV, BFV, and RRV (kindly provided by Klaus Grywna [Grywna et al., 2010]) were amplified in BHK-J cells. CHIKV was derived from an infectious cDNA clone as described earlier (Kümmerer et al., 2012). Virus-containing supernatants clarified from cell debris were aliquoted and frozen at −80°C. Viral titers of SFV, SINV, and VSV were determined by flow cytometry using anti-dsRNA antibody J2 (Schonborn et al., 1991), or anti-VSV G I14 (clone 1E9F9 [Lefrancois and Lyles, 1982]) combined with fluorescent secondary antibodies. Viral titers of EEEV, VEEV, MAYV, ONNV, BFV, RRV, and CHIKV were determined by plaque assay using BHK-J cells (Karliuk et al., 2021).

### Proteins

#### Expression and purification of His-NLRP1^PYD^, GST-NLRP1^PYD^, and active p38α

Expression vectors for human His-NLRP1^PYD^ (aa 1–92) and GST-NLRP1^PYD^ were generated by Gateway cloning with destination vector pDEST17 (Thermo Fisher Scientific) and a customized destination vector based on pGEX-2T (a kind gift of Mikko Taipale, Whitehead Institute of Biomedical Research, Cambridge, MA). P38α-His as well as untagged, constitutively active MKK3 S218E S222E were cloned into pET-Duet by Gibson cloning. Proteins were expressed in *Escherichia coli* LOBSTR (Andersen et al., 2013; His-NLRP^PYD^, and p38α-His + MKK3 S218E S222E) or *E. coli* BL21 (GST-NLRP1^PYD^) cells in Terrific Broth induced with 0.2 mM IPTG at an OD_600_ of 0.6. Cells were cultivated for 20 h at 18°C, and lysed by French press or sonication. His-NLRP^PYD^ and MKK3-activated p38α-His were purified by Ni-NTA affinity chromatography and gel filtration with a HiLoad 16/600 Superdex 75 pg column in buffers containing 50 mM Tris, pH 8.0, and 500 mM NaCl (His-NLRP^PYD^), or 50 mM Hepes, pH 7.5, 150 mM NaCl, 0.5 mM TCEP (p38α-His). GST-NRLP1^PYD^ was purified by affinity chromatography with glutathione resin and gel filtration with a HiLoad 16/600 Superdex 75 pg column in buffers containing 50 mM Tris, pH 8.0, and 500 mM NaCl.

#### Expression and purification of MBP-NLRP1

Full-length human NLRP1 (aa 1–1,473) and NLRP1^NACHT-LRR^ (aa 230–990) constructs were expressed as N-terminal MBP fusion proteins in baculovirus-infected *Spodoptera frugiperda* (*Sf9*) insect cells. For expression, 0.5 liters of *Sf9* cells at a density of 2.0 × 10^6^ cells/ml were infected with 5% P2 virus and incubated at 28°C for 48 h before harvesting the cells by centrifugation at 1,125 × *g* for 20 min. Cells were washed once with cold PBS and then lysed by sonication in buffer A (50 mM Hepes, pH 7.5, 150 mM NaCl, 0.5 mM TCEP), freshly supplemented with 1 mM PMSF. The lysate was clarified by centrifugation at 75,000 × *g* for 30 min at 10°C. Proteins were affinity purified using an MBPTrap HP (GE Healthcare) affinity column and eluted in buffer A supplemented with 10 mM maltose.

#### Expression and purification of nanobodies

Nanobody coding sequences were cloned into pHEN6-based bacterial, periplasmic expression vectors with C-terminal HA-His_6_ tags using Gibson cloning (New England Biolabs). Nanobodies were produced in *E. coli* WK6 transformed with the respective expression vectors. Expression cultures were grown in Terrific Broth, and expression was induced with 1 mM IPTG at an OD_600_ of 0.6, followed by cultivation at 30°C for 16 h. Bacterial pellets were resuspended in TES buffer (200 mM Tris-HCl, pH 8.0, 0.65 mM EDTA, 0.5 M sucrose), and periplasmic extracts generated by osmotic shock in 0.25× TES, followed by Ni-NTA purification and desalting by PD MiniTrap G-25 columns (GE Healthcare Life Sciences).

### Antibodies

The following antibodies were used: rabbit polyclonal anti-ASC (Cat# AG-25B-0006, RRID:AB_2490440; AdipoGen), mouse anti-Caspase-1 clone Bally-1 (Cat# AG-20B-0048, RRID:AB_2490257; AdipoGen), rabbit anti-caspase-3 clone D3R6Y (Cat# 14220, RRID:AB_2798429; Cell Signaling Technology), rabbit polyclonal anti-E-tag-HRP (Cat# A190-133P, RRID:AB_345222; Bethyl), rabbit anti-FLAG clone D6W5B (Cat# 14793, RRID:AB_2572291; Cell Signaling Technology), mouse anti-GAPDH clone 0411 (Cat# sc-47724, RRID:AB_627678; Santa Cruz), mouse anti-GFP clone JL-8 (Cat# 632380, RRID:AB_10013427; Takara Bio), mouse anti-γH2A.X (phospho S139) clone 9F3 (Cat# ab26350, RRID:AB_470861; Abcam), mouse anti-HA-HRP clone 6E2 (Cat# 2999S, RRID:AB_1264166; Cell Signaling Technology), mouse anti-IL-1β clone 3A6 (Cat# 12242, RRID:AB_2715503; Cell Signaling Technology), rabbit polyclonal anti-MKK3b (Cat# 9238, RRID:AB_2140797; Cell Signaling Technology), rabbit anti-phospho-MKK3 (Ser189)/MKK6 (Ser207) clone 22A8 (Cat# 9236, RRID:AB_491009; Cell Signaling Technology), mouse anti-NLRP1 clone 9F9B12 (Cat# 679802, RRID:AB_2566263; BioLegend), rabbit polyclonal anti-p38 (Cat# 9212, RRID:AB_330713; Cell Signaling Technology), rabbit anti-phospho-p38 (T180/Y182) clone D3F9 (Cat# 4511, RRID:AB_2139682; Cell Signaling Technology), rabbit anti-PARP clone 46D11 (Cat# 9532, RRID:AB_659884; Cell Signaling Technology), mouse anti-dsRNA clone J2 (Cat# 10010200, RRID:AB_2651015; SCICONS), mouse anti-vinculin clone hVIN-1 (Cat# V9131, RRID:AB_477629; Sigma-Aldrich), mouse anti-VSV G I14 clone 1E9F9 (from D.S. Lyles, kindly provided by Ari Helenius, ETH Zurich, Zurich, Switzerland), rabbit polyclonal anti-ZAK (Cat# A301-993A, RRID:AB_1576612; Bethyl laboratories), goat polyclonal anti-rabbit IgG (H + L)-Alexa Flour Plus 647 (Cat#A32733, RRID:AB_2633282; Invitrogen), goat polyclonal anti-mouse IgG (H + L)-Alexa Flour Plus 647 (Cat#A32728, RRID:AB_2633277; Invitrogen), goat polyclonal anti-mouse IgG (H + L)-DyLight 405 (Cat#35500BID, RRID:AB_2533208; Invitrogen), goat polyclonal anti-rabbit IgG (H + L)-HRP (Cat#31460, RRID:AB_228341; Invitrogen), goat polyclonal anti-mouse IgG (H + L)-HRP (Cat#31430, RRID:AB_228307; Invitrogen).

### Small compound inhibitors and reagents

The following small compound inhibitors and reagents were used: 2-Deoxy-D-glucose (Sigma-Aldrich), anisomycin (Sigma-Aldrich), bafilomycin A1 (Sigma-Aldrich), berzosertib (Selleckchem), bestatin methyl ester  (Abcam), blasticidin (InvivoGen), bortezomib (Selleckchem), cycloheximide (Sigma-Aldrich), deoxyribonucleic acid sodium salt from herring testes (Sigma-Aldrich), doramapimod (Cayman), doxycycline (Thermo Fisher Scientific), doxorubicin (Selleckchem), etoposide (Calbiochem), H_2_O_2_ (Roth), ISRIB (Selleckchem), JNK-IN-8 (Selleckchem), KU-60019 (Selleckchem), lactimidomycin (Sigma-Aldrich), LPS-EK Ultrapure (Invivogen), MG-132 (Selleckchem), MLN4924 (MedChem Express), MLN7243 (ChemieTek), Nigericin sodium salt (Biomol), PF-3644022 (Sigma-Aldrich), SB202190 (Sigma-Aldrich), poly(dA:dT) (Invivogen), poly(I:C) low molecular weight and high molecular weight (Invivogen), sodium azide (Sigma-Aldrich), talabostat mesylate (MedChemExpress), Vx-765 (Selleckchem), and Z-VAD(Ome)-FMK (MedChemExpress). ZAKα inhibitor 6p (Yang et al., 2020) was kindly provided by IFM Therapeutics. 6p was synthesized and characterized by ^1^H NMR spectroscopy and LC-MS, with the data being fully consistent with literature values for this compound.

### Nanobody library generation

To raise VHHs against NLRP1^PYD^, two alpacas were five times immunized with 200 µg His-NLRP1^PYD^ using Imject Alum Adjuvant (Thermo Fisher Scientific) according to locally authorized protocols. VHH plasmid libraries in the M13 phagemid vector pD (pJSC) were generated as described before (Schmidt et al., 2016b). In brief, RNA from peripheral blood lymphocytes was extracted and used as a template to generate cDNA using three sets of primers (random hexamers, oligo[dT], and primers specific for the constant region of the alpaca heavy chain gene). VHH coding sequences were amplified by PCR using VHH-specific primers, cut with AscI and NotI, and ligated into pJSC linearized with the same restriction enzymes. *E. coli* TG1 cells (Agilent) were electroporated with the ligation reactions and the obtained ampicillin-resistant colonies were harvested, pooled, and stored as glycerol stocks.

### Nanobody identification

NLRP1^PYD^ VHHs were obtained by phage display and panning with a protocol modified from Schmidt et al. (2016b). *E. coli* TG1 cells containing the VHH library were infected with helper phage VCSM13 to produce phages displaying the encoded VHHs as pIII fusion proteins. Phages in the supernatant were purified and concentrated by precipitation. Phages presenting NLRP1PYD-specific VHHs were enriched using GST-NLRP1PYD immobilized to magnetic Pierce glutathione beads (Thermo Fisher Scientific). The retained phages were used to infect *E. coli* ER2738 and subjected to a second round of panning. 96 *E. coli* ER2837 colonies yielded in the second panning were grown in 96-well plates and VHH expression was induced with IPTG. VHHs leaked into the supernatant were tested for specificity using ELISA plates coated with control protein GST or GST-NRLP1PYD. Bound VHHs were detected with HRP-coupled rabbit anti-E-Tag antibodies (1:10,000), and the chromogenic substrate TMB. Reactions were stopped with 1 M HCl and absorption at 450 nm was recorded using a SpectraMax i3 instrument and the SoftMax Pro 6.3 Software (Molecular Devices). Positive candidates were sequenced and representative nanobodies were cloned into bacterial expression vectors for further analysis.

### Nanobody ELISA

To test nanobody candidates, GST-NLRP1^PYD^ or GST in PBS were immobilized on ELISA plates at a concentration of 1 μg/ml overnight. Serial tenfold dilutions of HA-tagged nanobodies in 10% FBS/PBS were incubated with the immobilized antigen, followed by incubation with HRP-coupled anti-HA (1:5,000), and the chromogenic substrate TMB. Reactions were stopped with 1 M HCl and absorption was measured at 450 nm using a SpectraMax i3 instrument and the SoftMax Pro 6.3 Software (Molecular Devices).

### Flow cytometry–based quantification of inflammasome assembly

To quantify the assembly of ASC-EGFP specks or recruitment of C1C-EGFP to ASC specks (“C1C specks”), cells were typically treated in 24-wells or 48-wells and analyzed by flow cytometry. 2.5 × 10^5^ HEK^NLRP1+ASC^ (or other derivatives of HEK 293T), or 10^5^ N/TERT-1^C1C-EGFP^ (or other derivatives of N/TERT-1) cells were seeded per well and cultivated overnight in the absence of antibiotics. Cells were stimulated for 6 or 20 h in DMEM (10% FBS; HEK 293T) or keratinocyte serum-free medium (bovine pituitary extract, EGF, CaCl_2_; N/TERT-1). Importantly, N/TERT-1^C1C-EGFP^ cells were stimulated in the presence of 100 µM VX for all flow cytometry experiments to prevent loss of responding cells by caspase-1–dependent pyroptosis.

For drug-induced inflammasome stimulation, cells were generally treated with 15 μM Aniso, 20 μg/ml Blasti, 1 mM CHX, 1.5 mM H_2_O_2_, 2 μM Lacti, 200 ng/ml LPS followed by 10 μM Nig, 10 mM sodium azide, and 50 mM 2-DG, or 30 μM Tal. For UV-induced inflammasome stimulation, cells were irradiated in tissue culture plates (without lid) in a Bio-Link UVB irradiation system equipped with 5 × 8 Watt T-8.M tubes emitting UVB at 312 nm for 3 min, followed by cultivation for 20 h. For nucleic acid-induced inflammasome stimulation, cells were transfected with 1 μg/ml poly(I:C), poly(dA:dT), or HT-DNA using Lipofectamine 2000 (LF; Thermo Fisher Scientific). Medium was replaced with full medium after 6 h, and cells were cultivated for another 14 h. For virus-induced inflammasome stimulation, cells were infected for 1 h in serum-free medium at the indicated MOI, ranging from 1 to 50. Medium was subsequently replaced with full medium and cells were cultivated for another 19 h. For inflammasome stimulation by transient overexpression of HA- or FLAG-tagged HRV14 3C protease, VHHs and VHL-VHH fusions, p38 isoforms, MAP2K MKK1/3/4/5/6, or the catalytic domain of MAP3K TAOK2 in HEK-based reporter cells, cells were transfected with 500 ng total of expression vectors based on pCAGGS (Hitoshi et al., 1991; HRV14 3C, VHHs, or VHL-VHH fusions) or Gateway-compatible derivatives of pcDNA3.1 (kinases) using LF (Thermo Fisher Scientific) or PEI Max (Polysciences). Medium was replaced with full medium after 4–6 h and cells were cultivated for another 14–16 h. For induction of dox-inducible expression in lentivirus-generated cell lines, cells were cultivated in medium containing 1 µg/ml dox for 6 or 20 h.

In case of additional inhibitor treatments, small compound inhibitors were added 30 min before and during the stimulation, with the following concentrations: 100 nM BafA, 20 µM BeMeEs, 1 µM Borte, 10 µM Dora, 200 nM ISRIB, 3 µM Jnk, 1 µM MG-132, 1 μM MLN4, 1 μM MLN7243, 1 μM PF, 20 μM SB, 100 µM VX, 50 µM Z-VAD, or 100 nM ZAKα inhibitor 6p.

After 6 or 20 h stimulation, cells were harvested by trypsinization, fixed in 4% formaldehyde, and analyzed using BD FACSCanto and BD LSRFortessa SORP flow cytometers, recording area, width, and height of the EGFP signal of single cells. Where indicated, fixed and permeabilized cells were stained with anti-FLAG (1:300), anti-phospho-p38 (1:500), anti-γH2A.X (phospho S139; 1:500), anti-dsRNA (1:500), or anti-VSV G (1:1,000) in Intracellular Staining Permeabilization Wash Buffer (Biolegend) combined with DyLight 405, or Alexa Flour Plus 647–coupled, highly cross-absorbed secondary antibodies (1:500). Flow Cytometry data was analyzed using FlowJo 10.8.1 software. To determine the frequency of cells with ASC specks, cell debris and doublets were excluded based on forward and sideward scatter signals. Only cells expressing the ASC speck reporter ASC-EGFP or C1C-EGFP were included in the analysis of the ASC speck response based on height and width of the reporter signals. For the quantification of specks after virus infection or transient transfection, only cells positive for dsRNA/VSV G, or FLAG were included in the analysis (with the exception of mock-infected or untreated controls).

### Cytokine quantification by homogeneous time resolved fluorescence (HTRF)

To quantify IL-1β secretion, N/TERT-1–derived cells were seeded (10^5^ cells per well in 24-well or 48-well plates) and stimulated as described for flow cytometry experiments in the absence and presence of VX. Supernatants for the quantification of IL-1β levels after inducible expression of VHL-VHH fusions were collected from 5 × 10^4^ cells per well in 96-well plates.

IL-1β was quantified using the Human IL1 β HTRF kit (Ciscbio) according to the manufacturer’s instructions. Emissions at 620 and 665 nm were measured using a SpectraMax i3 instrument and IL-1β levels were calculated by the SoftMax Pro 6.3 Software (Molecular Devices) based on the standard curve.

### Cell death quantification by LDH release

To quantify cell death by pyroptosis, N/TERT-1–derived cells were seeded (10^5^ cells per well in 48-well plates) and stimulated as described for flow cytometry experiments in the absence and presence of VX. Supernatants for the quantification of cell death after inducible expression of VHL-VHH fusions were collected from 5 × 10^4^ cells per well in 96-well plates.

Release of LDH was quantified using the LDH Cytotoxicity Detection Kit (TaKaRa) according to the manufacturer’s instructions. Absorption at 490 nm was measured using a SpectraMax i3 instrument and the SoftMax Pro 6.3 Software (Molecular Devices). Control samples, in which cells were lysed in 1% Triton X-100, were used to normalize LDH release, after subtraction of medium background signal.

### Cell death quantification by DRAQ7 uptake

To quantify cell death over time, the uptake of the non-cell permeable DNA dye DRAQ7 (Biolegend) was analyzed using the Incucyte Live-Cell Imaging system (Sartorius). 1.5 × 10^4^ N/TERT-1^C1C-EGFP^ (or other derivatives of N/TERT-1) were seeded per well in 96-well plates and cultivated overnight in the absence of antibiotics. The next day, the cells were stimulated as described for flow cytometry experiments using medium containing DRAQ7 (1:3,000). Stimulation was performed in the absence and presence of VX, Z-VAD, or Dora. The cells were imaged every hour for a total of 20 h using the Incucyte SX5 instrument, taking nine images per well. The number of DRAQ7-positive nuclei (cell death count) and the cell confluency were analyzed using the Incuycyte 2021C software. For every single image, the cell death count was corrected by subtraction of the value at the start of the experiment. The corrected cell death count was further normalized to the cell confluency by division, before the average value for all nine images was calculated and plotted over time.

### Microscopy

To generate microscopy samples, cells were seeded on 12 mm cover slips in 24-well plates and otherwise stimulated as described for flow cytometry experiments. Cells were fixed in 4% formaldehyde and stained for DNA using Hoechst 33342 (1:5,000; Thermo Fisher Scientific). Images were recorded with a Zeiss Observer.Z1 wide field microscope, or a Leica SP8 Lightning confocal microscope. For live cell imaging experiments, N/TERT-1^C1C-EGFP^ cells were seeded in tissue culture-treated CellCarrier-96 Ultra Microplates (Perkin Elmer) and treated with the indicated stimuli in the presence of 3.3 µg/ml propidium iodide (PI; Biolegend). Cells were cultivated at 37°C, 5% CO_2_, and images were recorded every 20 min for 20 h using a Zeiss Observer Z1 wide-field microscope with 20X PlanApochromat objective (NA = 0.8). Images were processed using ImageJ 2.3.0 software.

### Immunoblot

To confirm expression or knockout of proteins in cell lines, 10^6^ cells were lysed in 200 μl 1× SDS-PAGE buffer (50 mM Tris, pH 6.8; 0.01% Bromophenol blue, 10% glycerol, 2% SDS, 100 mM dithiothreitol) to generate immunoblot samples. To confirm the dox-inducible reconstitution of NLRP1 in NLRP1 knockout N/TERT-1^C1C-EGFP^ cells, 8 × 10^5^ cells per well were seeded in a 6-well plate, followed by dox stimulation and lysis in 150 μl 1× SDS-PAGE buffer the next day. The same setup was used to analyze the phosphorylation of p38 and MKK3 in N/TERT-1^C1C-EGFP^ cells after Aniso or Tal stimulation in presence of ZAKα and p38 inhibitors. To analyze p38 phosphorylation in HEK 293T and keratinocyte reporter cells after UV and Aniso stimulation, 1.5 × 10^6^ HEK cells and 6 × 10^5^ N/TERT-1 cells per well were seeded in a 6-well plate, followed by stimulation and lysis in 500 μl 1× SDS-PAGE buffer the next day. To analyze the processing of caspase-1, caspase-3, PARP, and IL-1β, N/TERT-1^C1C-EGFP^ cells were seeded into 10 cm dishes and grown until they were around 70% confluent. The stimulation was performed in 6 ml medium. After stimulation, the cells were lysed in 400 μl 1× SDS-PAGE buffer to generate lysate immunoblot samples. The supernatant was harvested, cleared by centrifugation at 1,000 × *g* for 10 min, and secreted proteins were precipitated using methanol and chloroform (Jakobs et al., 2013). Precipitated protein was resuspended in 50 μl 1× SDS-PAGE buffer to generate supernatant immunoblot samples. Proteins were separated by SDS-PAGE using 4–15% Criterion TGX Precast Midi protein gels (Bio-Rad). Separated proteins were transferred to polyvinylidene difluoride membranes (0.45 μm; Merck) by semi-dry transfer, except for immunoblots of MKK3 and P-MKK3 which were detected on nitrocellulose membranes (0.45 μm; GVS). All immunoblots were blocked in 5% non-fat dry milk (NFDM) solution in TBS-T (0.05% Tween-20) and probed with the following primary antibody dilutions: anti-ASC 1:1,000, anti-caspase-1 1:1,000, anti-caspase-3 1:500, anti-GAPDH 1:2,000, anti-GFP 1:1,000, anti-HA-HRP 1:1,000, anti-IL-1β 1:1,000, anti-MKK3 1:1,000, anti-P-MKK3 1:1,000, anti-NLRP1 1:1,000, anti-p38 1:1,000, anti-P-p38 1:1,000, anti-PARP 1:500, anti-vinculin 1:1,000, and anti-ZAK 1:1,000. All primary antibodies were added in NFDM solution, except anti-IL-1β, anti-MKK3, anti-P-MKK3, anti-p38, and anti-P-p38, which were added in TBS-T containing 3% BSA. After overnight incubation at 4°C, the immunoblots were probed with HRP-coupled secondary antibodies in NFDM solution (1:5,000) for 2 h. Chemiluminescent signal was induced by Western Lightning Plus-ECL (Perkin Elmer), except for immunoblots of ASC, caspase-1, caspase-3, IL-1β, MKK3, P-MKK3, NLRP1, PARP, and ZAKα, which required Western Lightning Ultra-ECL (Perkin Elmer). The signal was detected using a Fusion Advancer imaging system (Vilber) and images were taken using the EvolutionCapt SL6 software (Vilber).

### In vitro kinase assay

For in vitro kinase assays, 0.2 µM kinase was incubated with 7 µM substrate and 0.2 mM ATP containing 0.45 mCi [32P]-γ-ATP/ml (Perkin Elmer) in kinase buffer (50 mM Hepes, pH 7.6, 34 mM KCl, 7 mM MgCl2, 2.5 mM dithiothreitol, 5 mM β-glycerol phosphate). Reactions were incubated for 30 min at 30°C and 300 rpm and stopped by addition of EDTA to a final concentration of 50 mM. Samples were spotted onto Amersham Protran nitrocellulose membrane (GE Healthcare), followed by three washing steps for 5 min each with PBS. Counts per minute were determined in a Beckman Liquid Scintillation Counter (Beckman-Coulter) for 1 min. Measurements were performed in quadruplicate and represented as mean with SD.

### Identification of phosphosites by mass spectrometry (MS)

To reveal phosphorylation sites on NLRP1, HEK^NLRP1+ASC^ cells in 15 cm dishes (three dishes per immunoprecipitation) were stimulated with or without 15 uM Aniso for 1 h. Cells were lysed in low salt lysis buffer (1% IGEPAL CA-630, 50 mM Tris, pH 7.5, 150 mM NaCl, 1 mM EDTA) supplemented with HALT protease and phosphatase inhibitor cocktail (Thermo Fisher Scientific). After sedimentation of nuclei and debris, lysates were supplemented with NaCl to reach a final concentration of 500 mM. 50 μl Pierce magnetic anti-HA beads (Thermo Fisher Scientific) were subsequently incubated with the lysates to immunoprecipitate NLRP1-HA. Beads were washed five times with high salt lysis buffer (1% IGEPAL CA-630, 50 mM Tris, pH 7.5, 500 mM NaCl, 1 mM EDTA), once with detergent-free wash buffer (50 mM Tris, pH 7.5, 500 mM NaCl, 1 mM EDTA), and twice with 50 mM Tris. Proteins were then denatured in 6 M Urea, 50 mM Tris, pH 8.0, for 20 min on ice. Samples were reduced with 10 mM TCEP, alkylated with 40 mM chloroacetamide, and digested on beads with 1 µg trypsin and Lys-C (Promega) or chymotrypsin (Sigma-Aldrich) overnight. Peptides were desalted with SDB-RPS stage tips and resolubilized in buffer A (0.1% formic acid) before loading onto 50-cm columns packed in-house with C18 1.9 μm ReproSil particles (Dr. Maisch GmbH), with an EASY-nLC 1,200 system (Thermo Fisher Scientific) coupled to the Orbitrap Exploris 480 (Thermo Fisher Scientific). Temperature was maintained at 50°C and peptides were eluted with a 70-min gradient starting at 5% buffer B (80% ACN, 0.1% formic acid), followed by a stepwise increase to 30% in 55 min, 65% in 5 min, 95% in 5 min, and maintained at 95% for 5 min, at a flow rate of 300 nl/min. A data-dependent (TopN) acquisition MS method was used for proteome analysis in which one full scan (300–1,650 m/z, R = 60,000, ACG target 300%) was first performed, followed by 10 data-dependent MS/MS scans with higher-energy collisional dissociation (ACG target 100%, maximum injection time at 28 ms, isolation window 1.4 m/z, HCD collision energy 30%, R = 15,000). Dynamic exclusion of 30 s was enabled. Data was acquired with the Xcalibur software (Thermo Fisher Scientific) and MS raw files were processed with MaxQuant version 2.0.3. Fragment lists were searched against the human UniProt FASTA database (November 2021) with cysteine carbamidomethylation as a fixed modification and N-terminal acetylation, and serine/threonine/tyrosine phosphorylation and methionine oxidations as variable modifications. False discovery rate was set to <1% at the peptide and protein levels and a minimum ratio count of 1 was required for valid quantification events using MaxQuant’s LFQ algorithm (MaxLFQ). All bioinformatics analyses were done with Perseus (version 1.6.15.0). Data were filtered for common contaminants and hits to the reverse data database. For phosphosite analysis, the localization probability cutoff was set to 0.75.

### Online supplemental material

Fig. S1 shows data validating and characterizing the HEK 293T and N/TERT-1–based NLRP1 reporter cell lines. Figs. S2 and S3 provide additional data on the activation of NLRP1 by the ribotoxic stress response and respective controls. Fig. S4 represents immunoblots of lysates and supernatants of N/TERT-1 cells after NLRP1 activation. Fig. S5 shows immunoblots and SDS-PAGE gels validating HEK 293T and N/TERT-1 cell lines expressing NLRP1 mutants, as well as recombinant proteins used in the in vitro kinase assay. Table S1 shows sgRNA target sequences used for the generation of knockout cell lines. Videos 1, 2, 3, and 4 represent N/TERT-1^C1C-EGFP^ cells after activation of the ribotoxic stress response, visualizing inflammasome assembly and plasma membrane permeabilization.

## Supplementary Material

Table S1 shows sgRNA target sequences used for the generation of knockout cell lines.

SourceData F1 contains original blots for Fig. 1.

SourceData F2 contains original blots for Fig. 2.

SourceData F4 contains original blots for Fig. 4.

SourceData F5 contains original blots for Fig. 5.

SourceData FS1 contains original blots for Fig. S1.

SourceData FS2 contains original blots for Fig. S2.

SourceData FS4 contains original blots for Fig. S4.

SourceData FS5 contains original blots for Fig. S5.
