# Supplementary material for: P38 kinases mediate NLRP1 inflammasome activation after ribotoxic stress response and virus infection
Source: J Exp Med. 2022 Oct 31;220(1):e20220837. doi: 10.1084/jem.20220837 (PMC9623368; doi:10.1084/jem.20220837)
Supplement: Table S1 — shows sgRNA target sequences used for the generation of knockout cell lines. [file JEM_20220837_TableS1.docx]

Table S1. sgRNA target sequences used for the generation of knockout cell lines.

| Target gene | sgRNA name | Target sequence |
| --- | --- | --- |
| ASC | ASC sg5 | GCTGGATGCTCTGTACGGGA |
| NLRP1 | NLRP1 sg2 | CAGGCCCAATAGGAAACGTG |
| P38α | P38α sg1 (KO 1) | TGATGAAATGACAGGCTACG |
|  | P38α sg2 (KO 2) | CACAAAAACGGGGTTACGTG |
| P38β | P38β sg2 (KO 1) | GCTTCTGGACGTCTTCACGC |
|  | P38β sg3 (KO 2) | GGTGGATGATCCCGGCCGAG |
| TAOK2 | TAOK2 sg1 | TCAAGACAGACCAACCTCAG |
|  | TAOK2 sg2 | CCCAACACCATTCAGTACCG |
|  | TAOK2 sg3 | GCACTGAGTGGCTACTCTCG |
| ZAKα | ZAKα sg1 | TGTATGGTTATGGAACCGAG |
|  | ZAKα sg2 | GTGACAATGCCATAGTTGGG |
|  | ZAKα sg3 | TCCTACACAACAAGGCGGAG |
