# Supplementary figures and images for: P38 kinases mediate NLRP1 inflammasome activation after ribotoxic stress response and virus infection
Source: J Exp Med. 2022 Oct 31;220(1):e20220837. doi: 10.1084/jem.20220837 (PMC9623368; doi:10.1084/jem.20220837)

Figure 1G

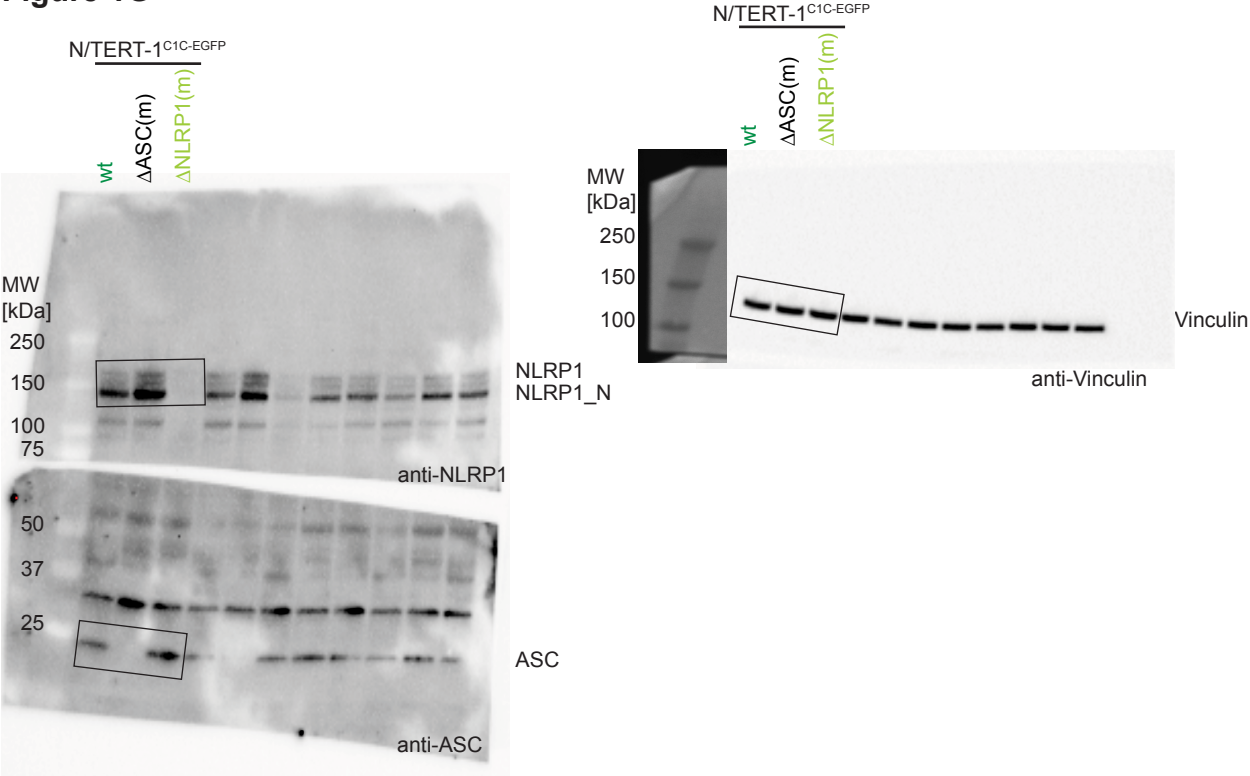

Supplement: SourceData F1 — contains original blots for Fig. 1. [file JEM_20220837_SourceDataF1.pdf]

Figure 2C

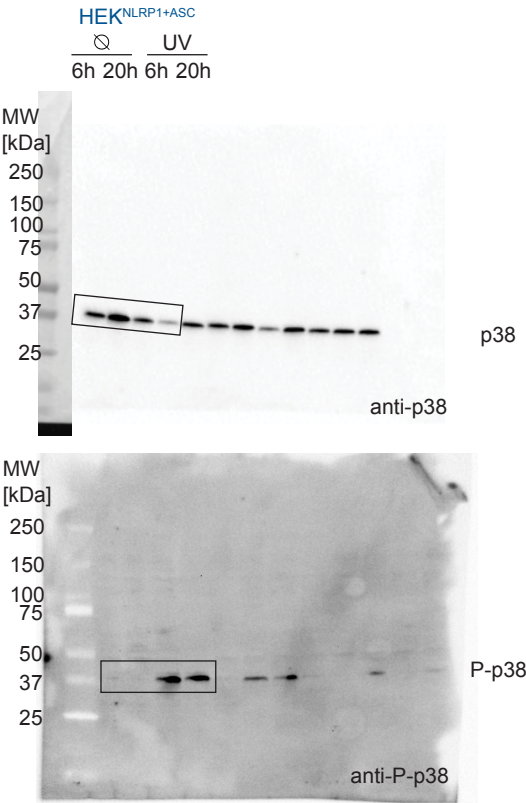

Figure 2D+E

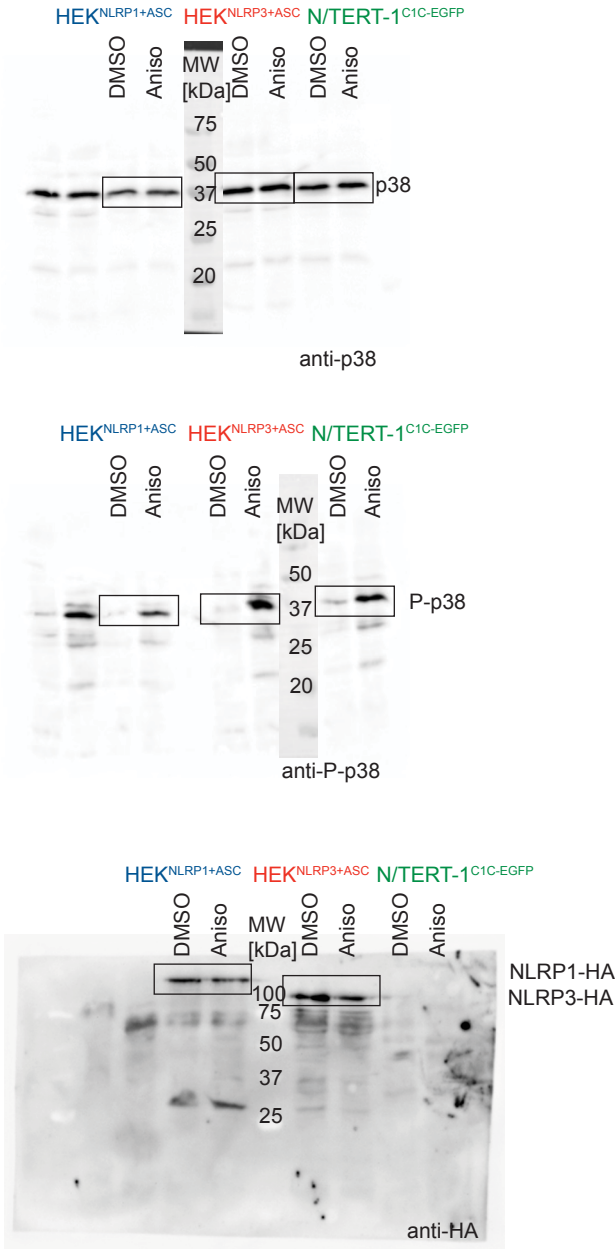

Figure 2D+E

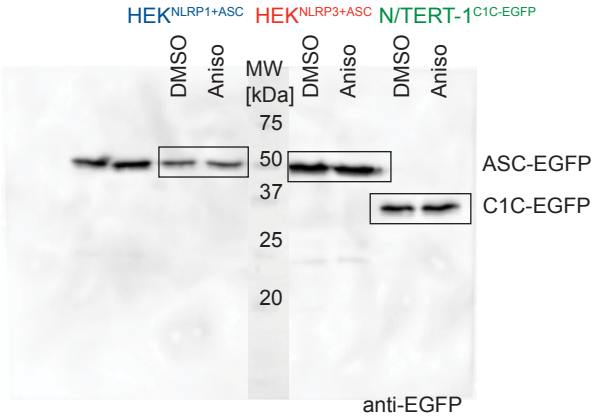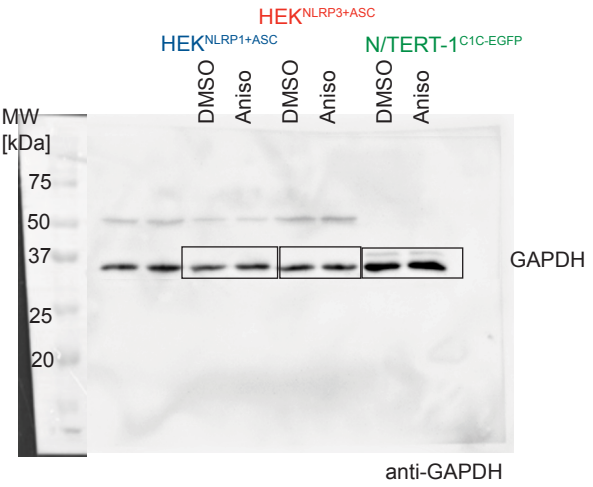

Figure 2L

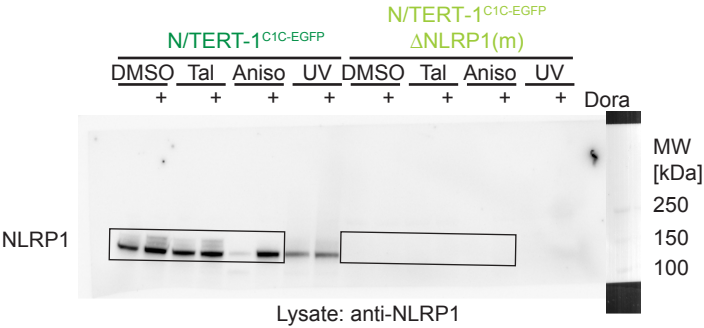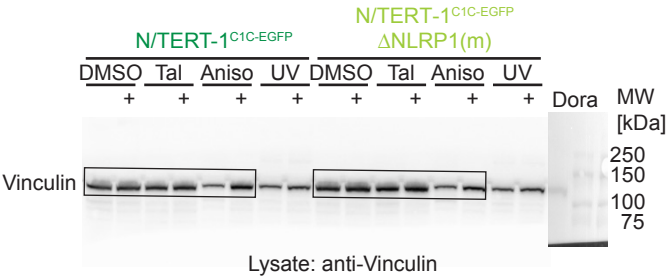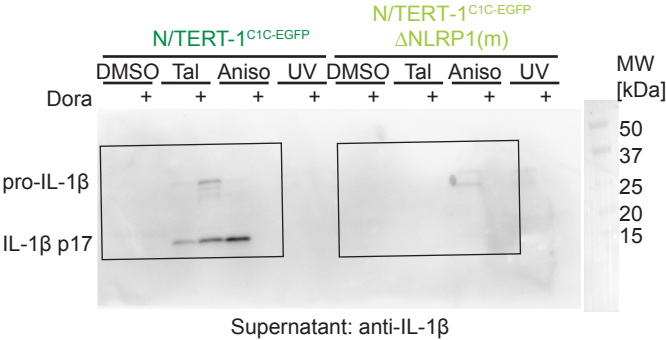

Figure 2L

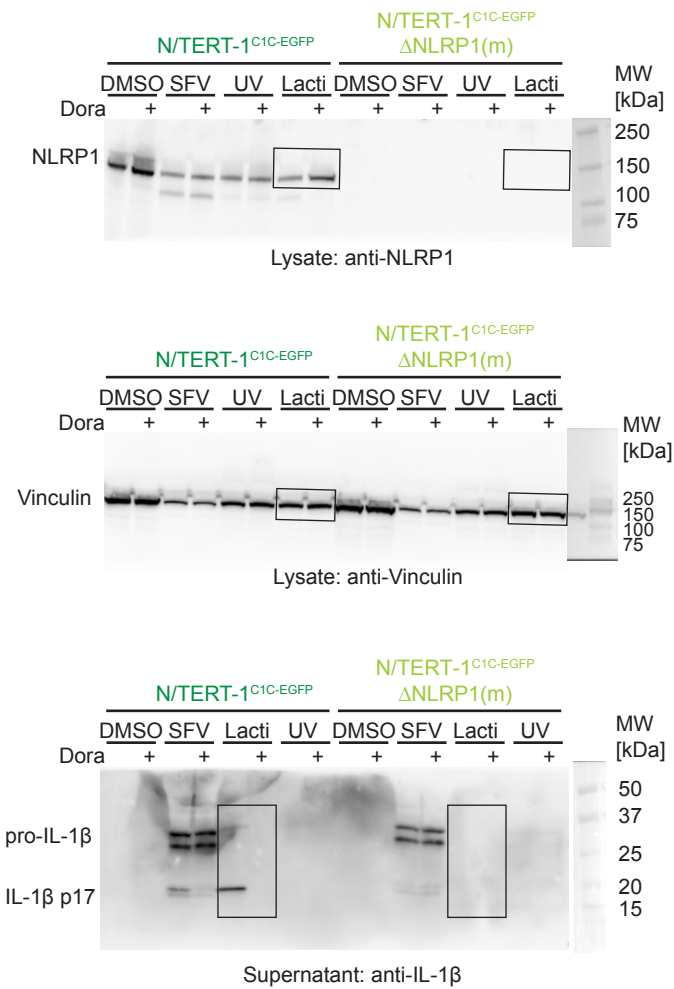

Supplement: SourceData F2 — contains original blots for Fig. 2. [file JEM_20220837_SourceDataF2.pdf]

Figure 4G

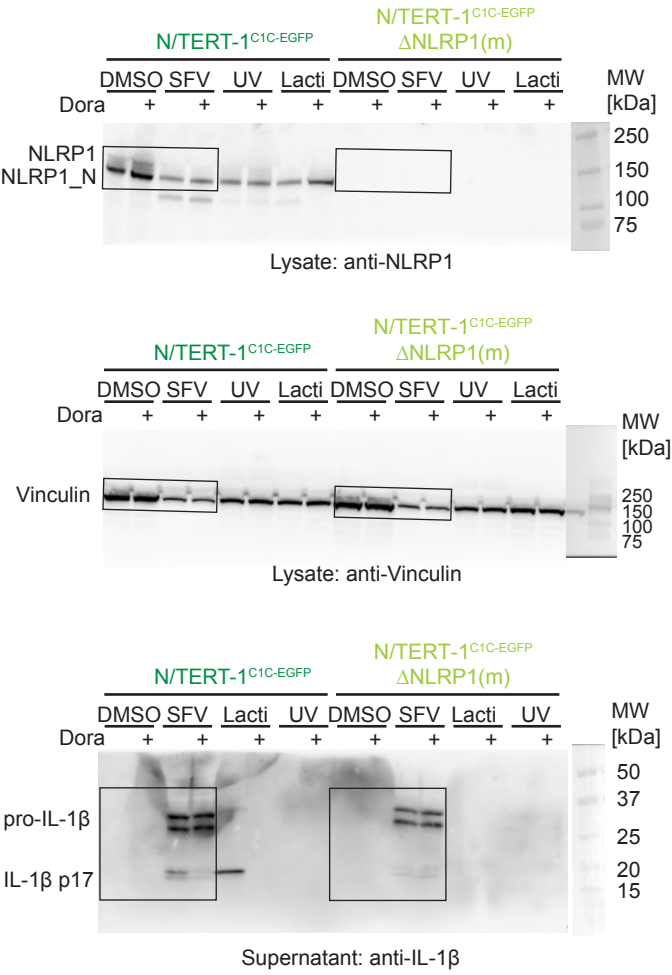

Supplement: SourceData F4 — contains original blots for Fig. 4. [file JEM_20220837_SourceDataF4.pdf]

Figure 5D

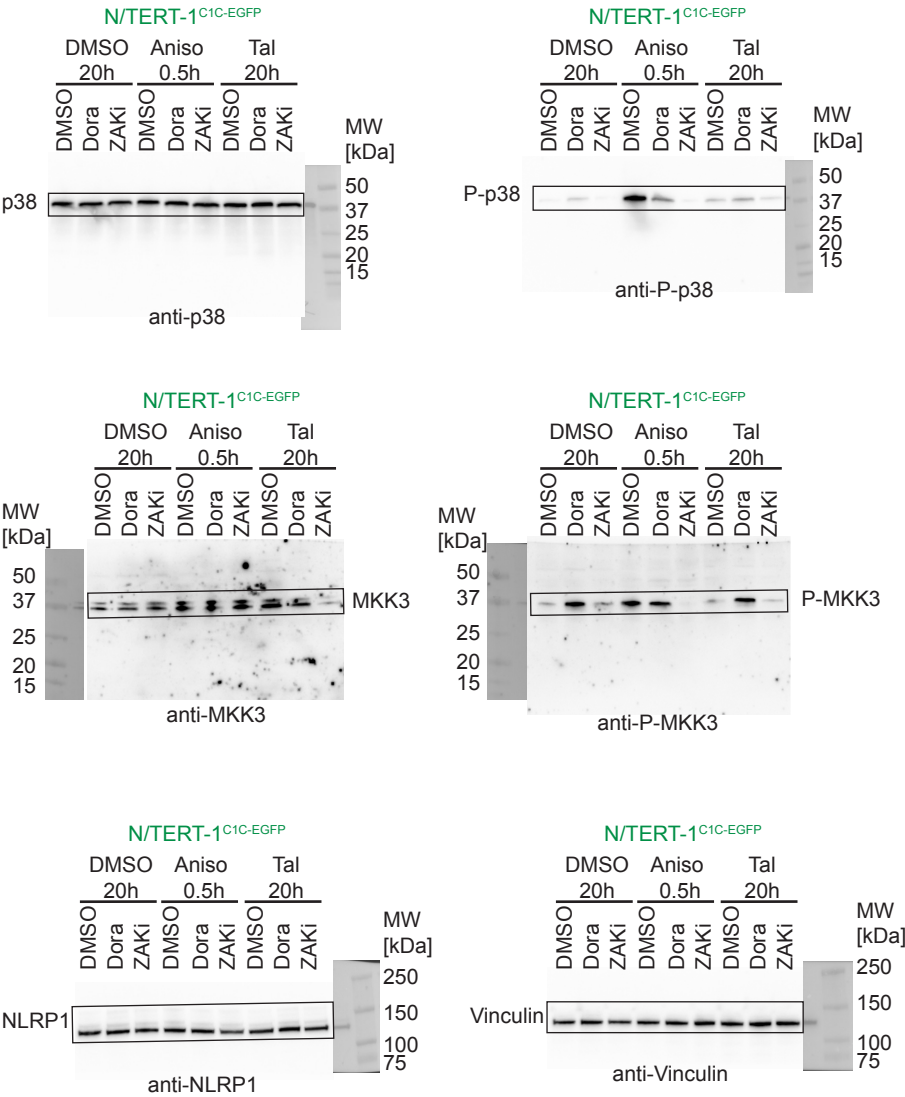

Figure 5F

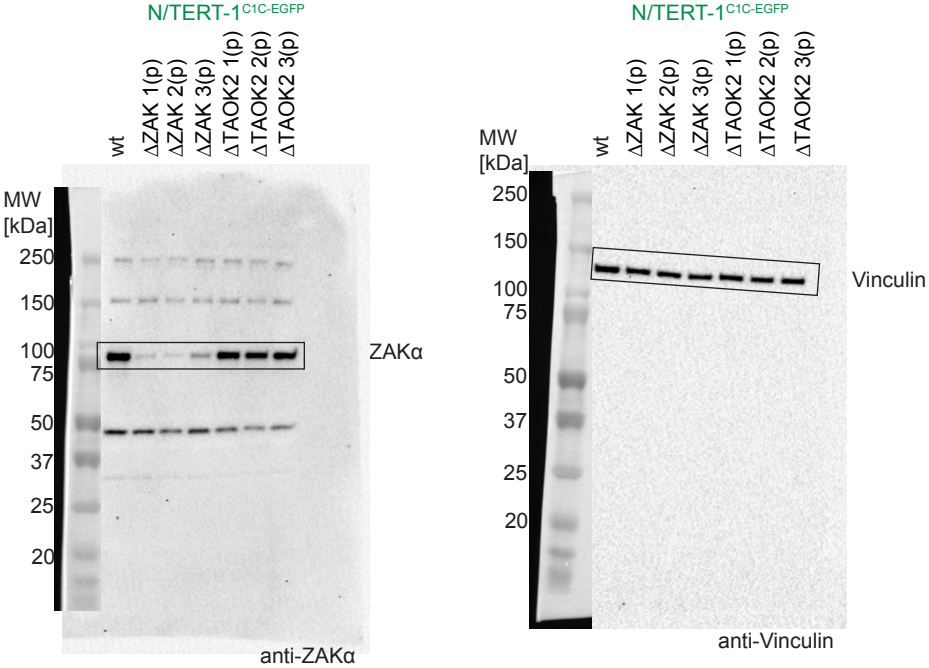

Supplement: SourceData F5 — contains original blots for Fig. 5. [file JEM_20220837_SourceDataF5.pdf]

Figure S1A + S1E

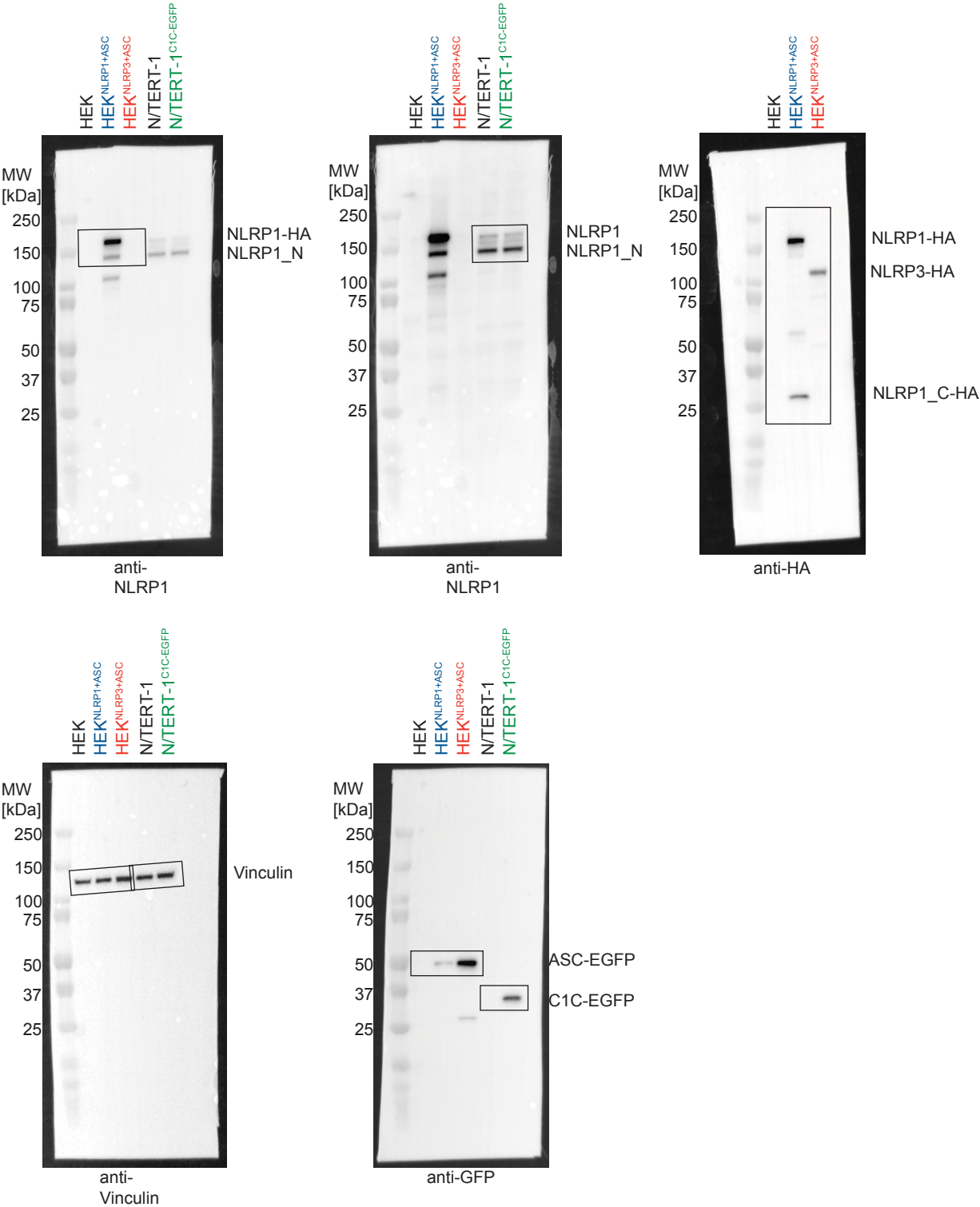

Supplement: SourceData FS1 — contains original blots for Fig. S1. [file JEM_20220837_SourceDataFS1.pdf]

Figure S2N

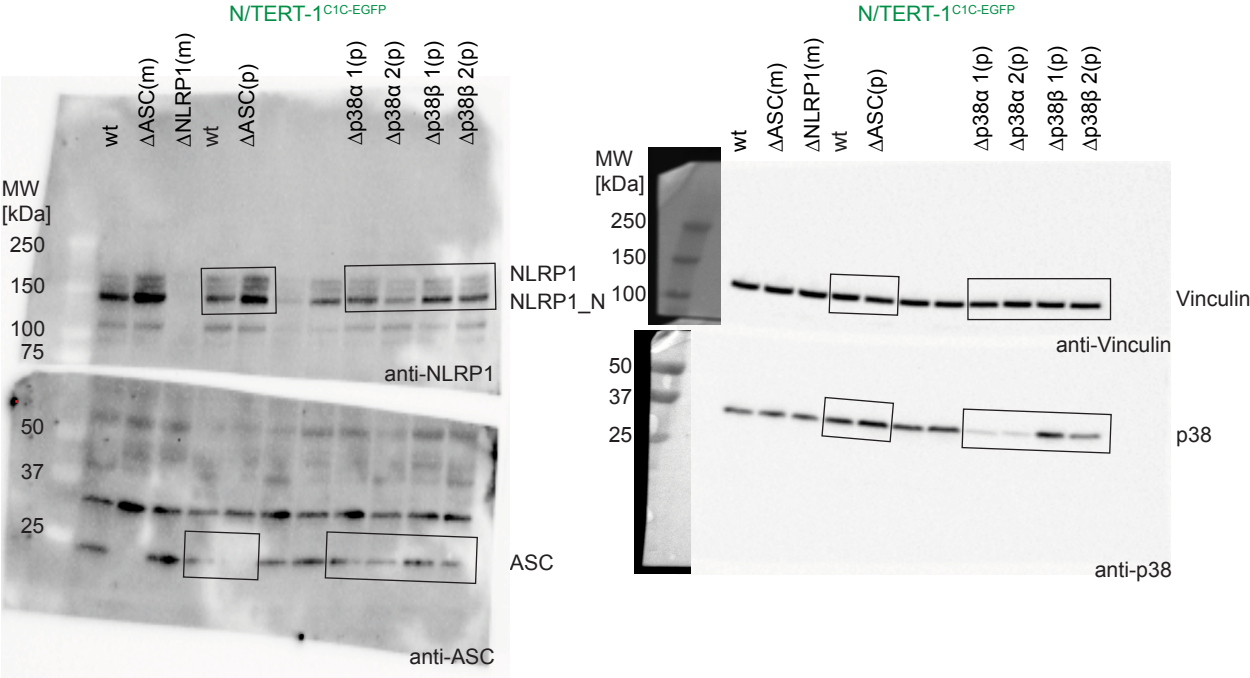

Supplement: SourceData FS2 — contains original blots for Fig. S2. [file JEM_20220837_SourceDataFS2.pdf]

Figure S4A

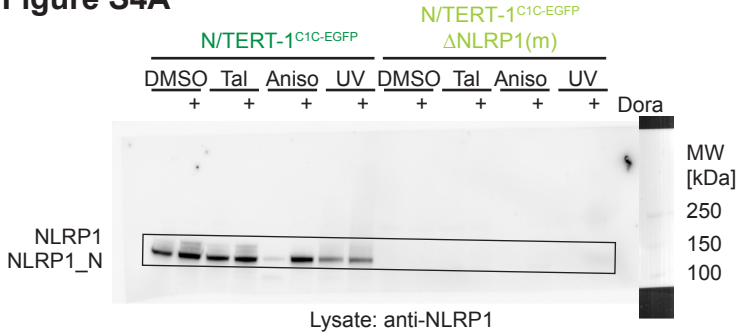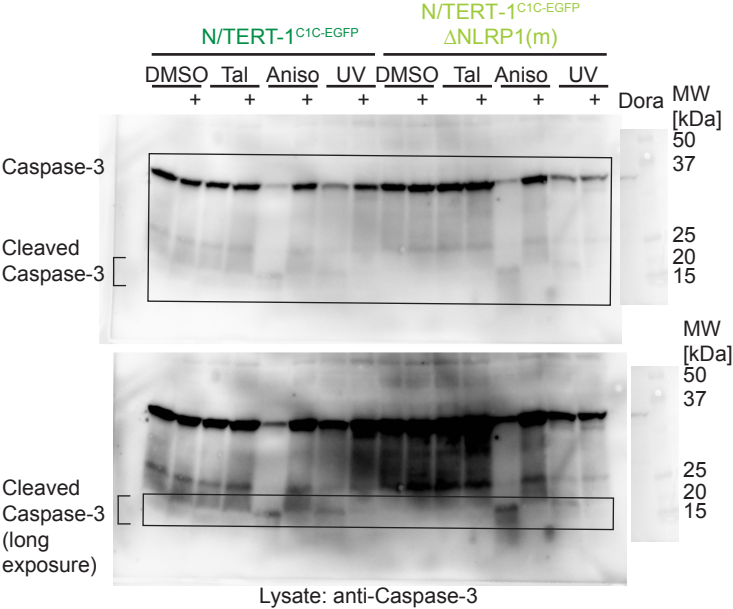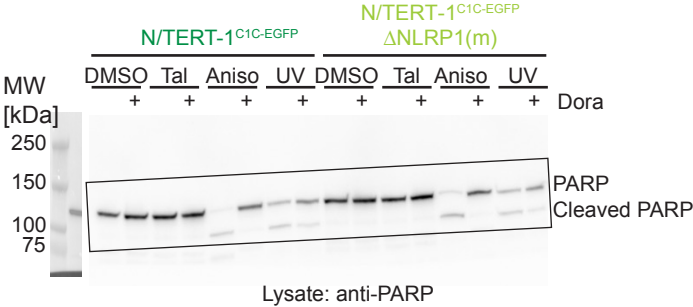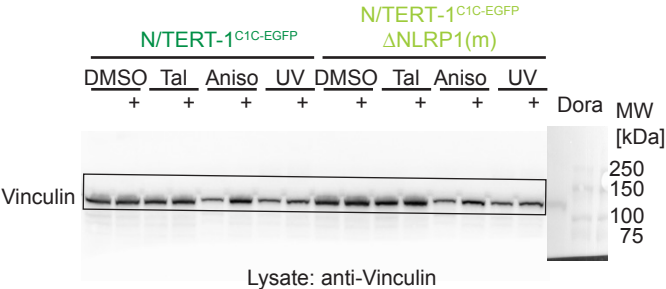

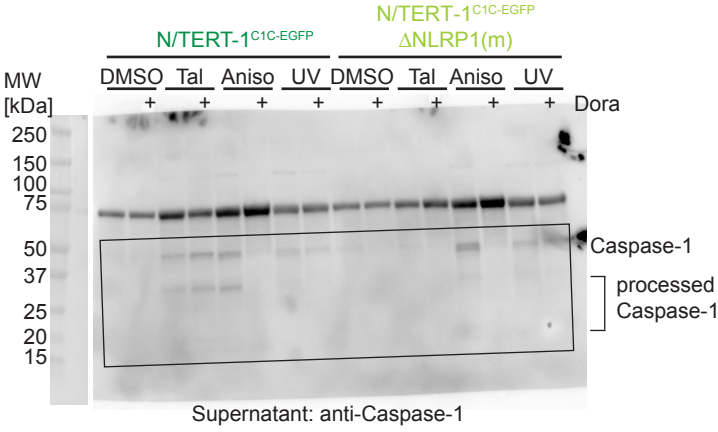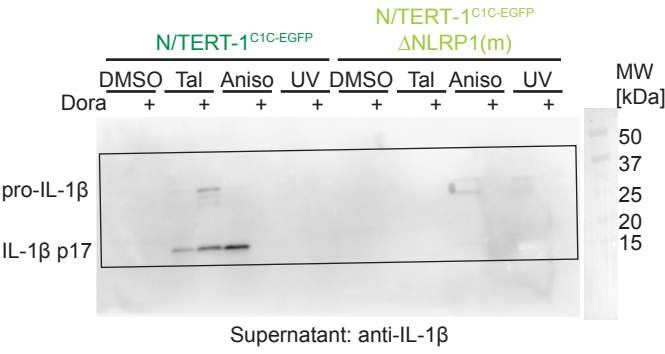

### Figure S4B

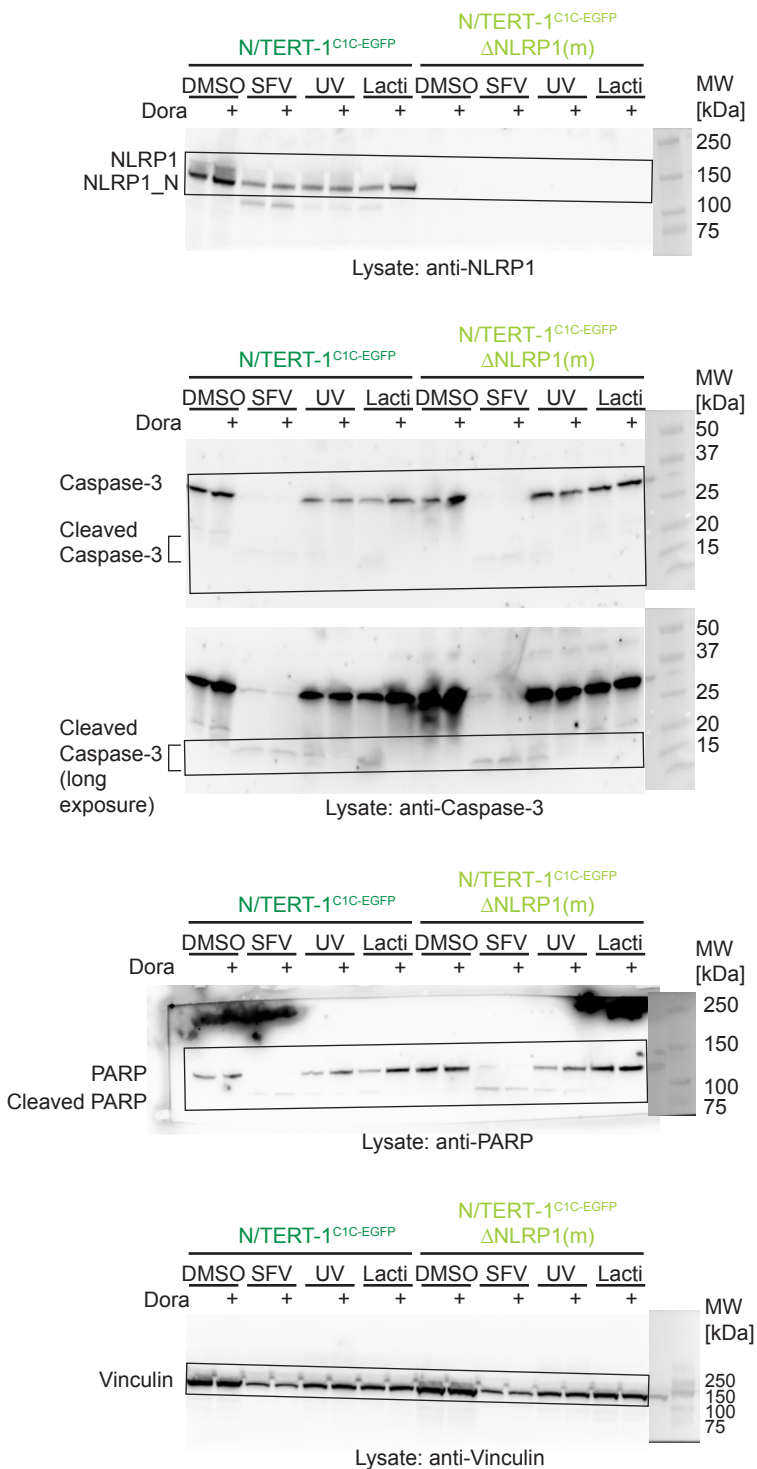

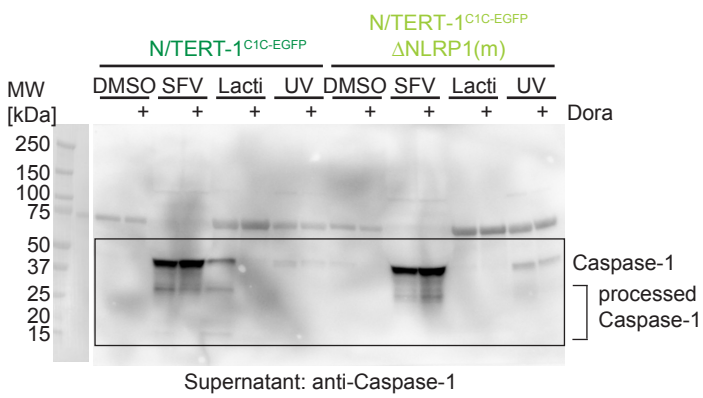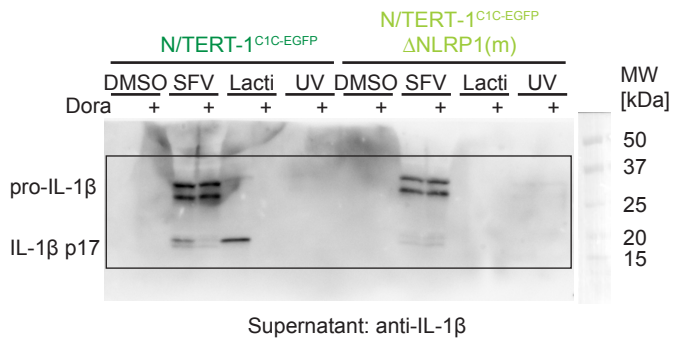

Supplement: SourceData FS4 — contains original blots for Fig. S4. [file JEM_20220837_SourceDataFS4.pdf]

Figure S5A

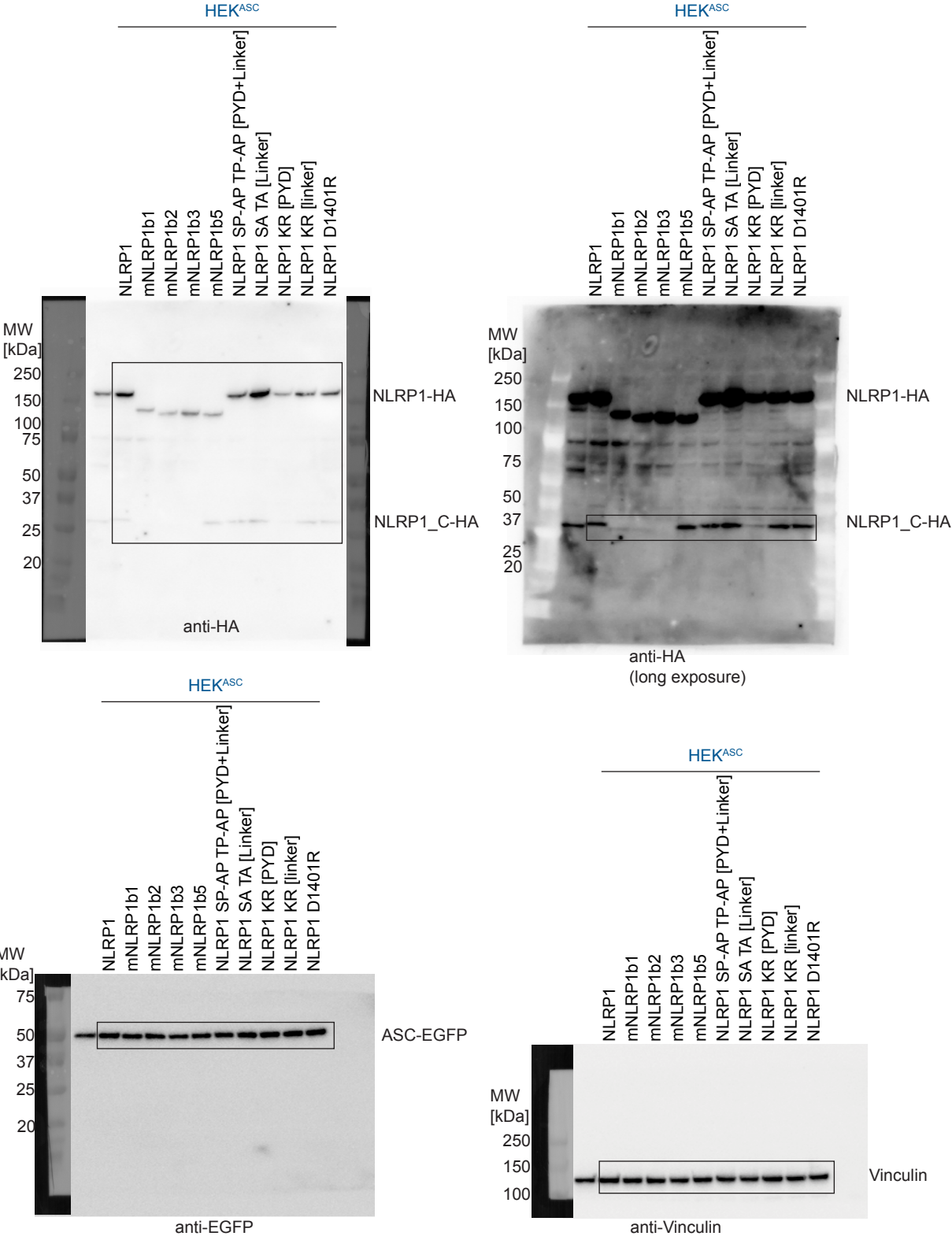

Figure S5C

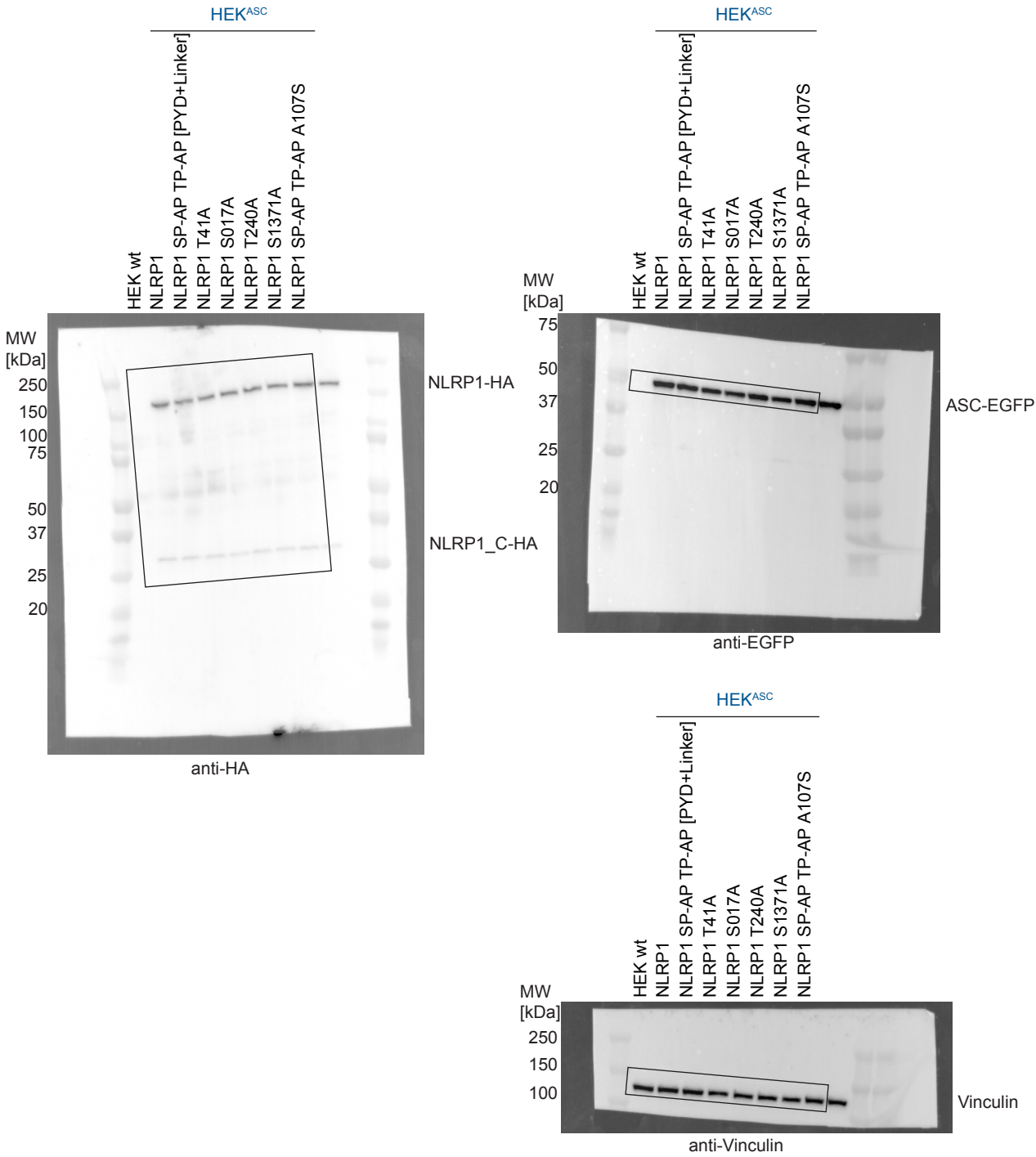

Figure S5D

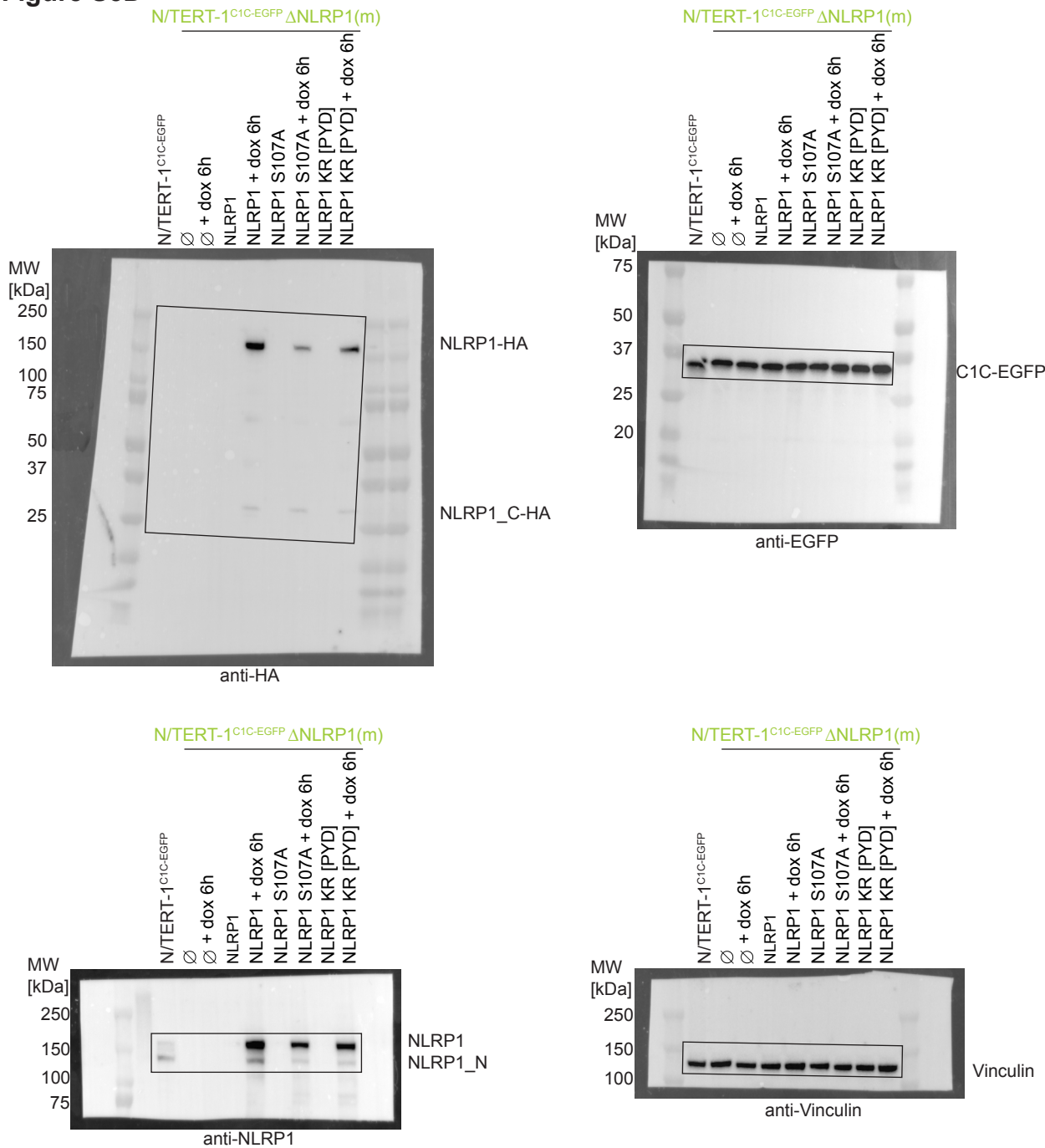

Supplement: SourceData FS5 — contains original blots for Fig. S5. [file JEM_20220837_SourceDataFS5.pdf]
